# Cycloazulenylene

**DOI:** 10.1002/anie.2204400

**Published:** 2026-07-16

**Authors:** Clara Douglas, Josefine Sprachmann, David Dunlop, Joël Schlecht, Jörg Neudörfl, Tommy Wachsmuth, Julian Frost, Tomáš Slanina, Oliver Dumele

**Affiliations:** ^1^ Department of Chemistry and Biochemistry University of Cologne Cologne Germany; ^2^ Department of Chemistry Humboldt Universität zu Berlin Berlin Germany; ^3^ Institute of Organic Chemistry and Biochemistry of the Czech Academy of Sciences Prague Czech Republic; ^4^ Department of Inorganic Chemistry Faculty of Science Charles University in Prague Prague Czech Republic

**Keywords:** azulene, cycloparaphenylenes, strained macrocycles

## Abstract

Nonalternant hydrocarbons differ significantly from their alternant analogs due to their nonsymmetric electronic structure, which manifests in distinct (anti)aromaticity, optical behavior, and redox properties. Subjecting such systems to strain and macrocyclic conjugation further changes these characteristics, leading to materials with fundamentally new properties. Azulene, a nonalternant hydrocarbon known for its anti‐Kasha fluorescence and intense blue coloration, is herein strained into a macrocycle along its 2,6‐positions. Alternating 2,2′ and 6,6′ linkages of the subunits form the symmetric all‐azulene analogue of [6]cycloparaphenylene, termed [6]cycloparaazulenylene ([6]CPA). Its structure was elucidated via single‐crystal X‐ray diffraction, revealing pronounced dihedral torsion between adjacent azulene subunits and an unusual tubular packing motif. The macrocycle exhibits electronic delocalization along the macrocyclic perimeter, a remarkably narrow HOMO–LUMO gap, and intense absorption in the visible range. Computational analyses of aromaticity show that, despite the 60 *π*‐electron (4*n*) macrocyclic perimeter, the overall electronic structure is dominated by the local aromaticity of the 10 *π*‐electron (4*n*+2) azulene subunits, while a weak paratropic ring current is induced upon application of an external magnetic field.

## Graphical abstract

A strained macrocycle consisting solely of azulene subunits, named [6]cycloparaazulenylene ([6]CPA), is presented. The macrocycle exhibits a notably narrow HOMO–LUMO gap for a pristine azulene species, strain‐induced red‐shifted absorption, and an unusual tubular packing motif elucidated via single‐crystal XRD. Aromaticity analysis shows that its electronic structure is dominated by the individual 10 *π*‐electron azulene subunits, while a weak paratropic current is induced within the 60 *π*‐electron macrocycle when an external magnetic field is applied.

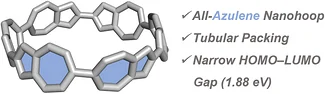

## 1 Introduction

**FIGURE 1:**
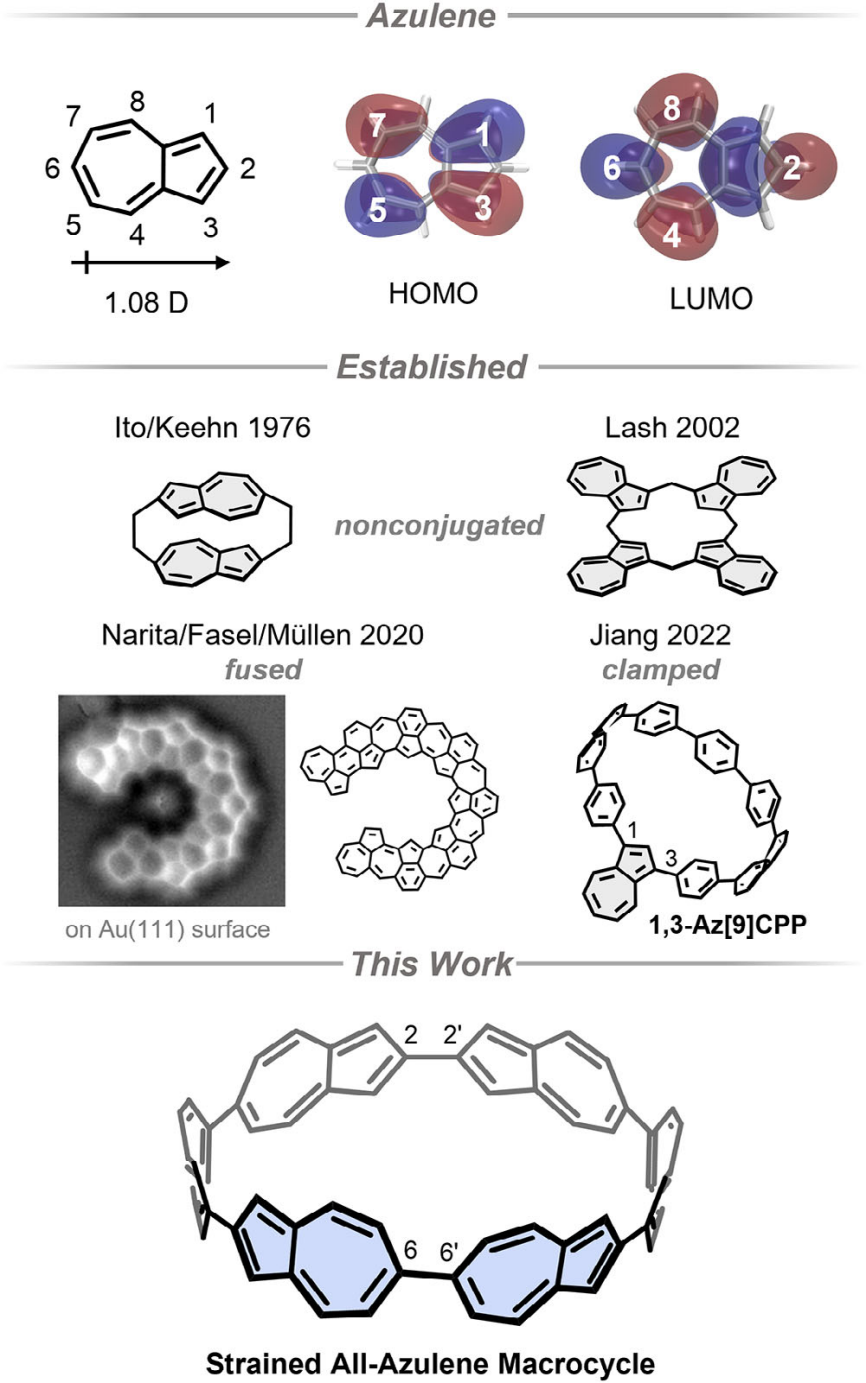
Resonance structure and FMOs of azulene, examples of azulenes incorporated in (semi)cyclic structures, and all‐azulene strained macrocycle, [6]cycloparaazulenylene **1**.

Azulene, a nonalternant constitutional isomer of naphthalene, can be viewed as the fusion of an electron‐rich five‐membered ring and an electron‐deficient seven‐membered ring. This inherent electronic asymmetry gives rise to a large molecular dipole moment (1.08 D) and unique optical properties, most notably anti‐Kasha emission from the *S*_2_ excited state [1, 2, 3]. The HOMO of pristine azulene is mainly located on its uneven‐numbered carbons, whereas the LUMO is mainly located on its even‐numbered carbons (Figure 1). This poor spatial overlap causes a small HOMO–LUMO gap that can be tuned through additional substitution [4]. The characteristic blue color of parent azulene stems from the weak *S*_0_→*S*_1_ (HOMO→LUMO) transition, absorbing at 580 nm [2]. As a result, azulenes have been implemented in a range of optical devices, such as solar cells and OFETs [5, 6], conductive polymers [7, 8, 9], as well as photoswitches [10, 11].

In more fundamental investigations, azulene has been incorporated into several nonconjugated cyclic systems that enable unique intra‐ and intermolecular interactions involving multiple azulene subunits. For example, in *anti*‐[2.2](2,6)azulenophane [12, 13], the antiparallel orientation of two azulene subunits is forced by symmetric methylene bridges, and in calixazulenes [14, 15, 16], a bowl‐shaped structure functions as a supramolecular host that can encapsulate fullerenes at its central cavity (Figure 1, middle).

More recently, the incorporation of azulenes into polyaromatic hydrocarbons (PAHs) has drawn attention, with the resulting fully *sp* ^2^‐carbon structures adopting their own unique optical and electronic characteristics [17, 18]. Thus, a number of ring‐fused azulene structures, including acenes [19], perylenes [20, 21], and larger nanographenes [22, 23, 24, 25, 26], have been reported, many of which show an open‐shell character in the ground state and small optical energy gaps. On‐surface synthetic approaches have achieved the lateral dehydrogenative coupling of 2,6‐polyazulene chains to yield nanoribbons containing various fused 5–7 ring pairs [27, 28]. In 2020, Narita, Fasel, Müllen, and co‐workers reported such a fused nanostructure resulting from 1,3‐substituted azulene. Notably, AFM imaging on an Au(111) surface revealed an angular fusion pattern of multiple azulenes resembling an incomplete cyclic fused, flat structure (Figure 1, middle) [29].

In 2022, Jiang and co‐workers reported an azulene‐embedded carbon nanohoop **1,3**‐**Az[9]CPP** [30], which features a azulene subunit clamped at the 1,3‐positions (Figure 1, middle). The 1,3‐substitution pattern causes it to twist into the plane of the macrocycle, thereby limiting electronic delocalization through the extended *π*‐system. Nevertheless, the HOMO–LUMO gap of **1,3**‐**Az[9]CPP** (2.84 eV) is significantly narrowed in comparison to pristine azulene (3.37 eV), demonstrating the potential effects of incorporating azulene into a strained aromatic system. Several other benzenoid nonalternant hydrocarbons [31], such as fluoranthrene [32], rubicene [33, 34], and dibenzopentalene [35, 36, 37], have been incorporated into CPP backbones, either through reductive aromatization of cyclohexadiene clamps or reductive elimination of Pt‐ or Au‐complexes formed with linear aromatic linkers [38, 39, 40].

Despite these advances, to date, no macrocycle composed exclusively of azulene subunits—regardless of topology or connectivity—has been reported. Cycloazulenylenes therefore remain entirely elusive. In this work, we report a fully conjugated strained macrocycle (nanohoop) constructed from six azulene subunits linked in alternating fashion at the 2,2′‐ and 6,6′‐positions to resemble the azulenylene‐analogue of [6]cycloparaphenylene ([6]CPP), named [6]cycloparaazulenylene **1** ([6]CPA).

## 2 Results and Discussion

**SCHEME 1:**
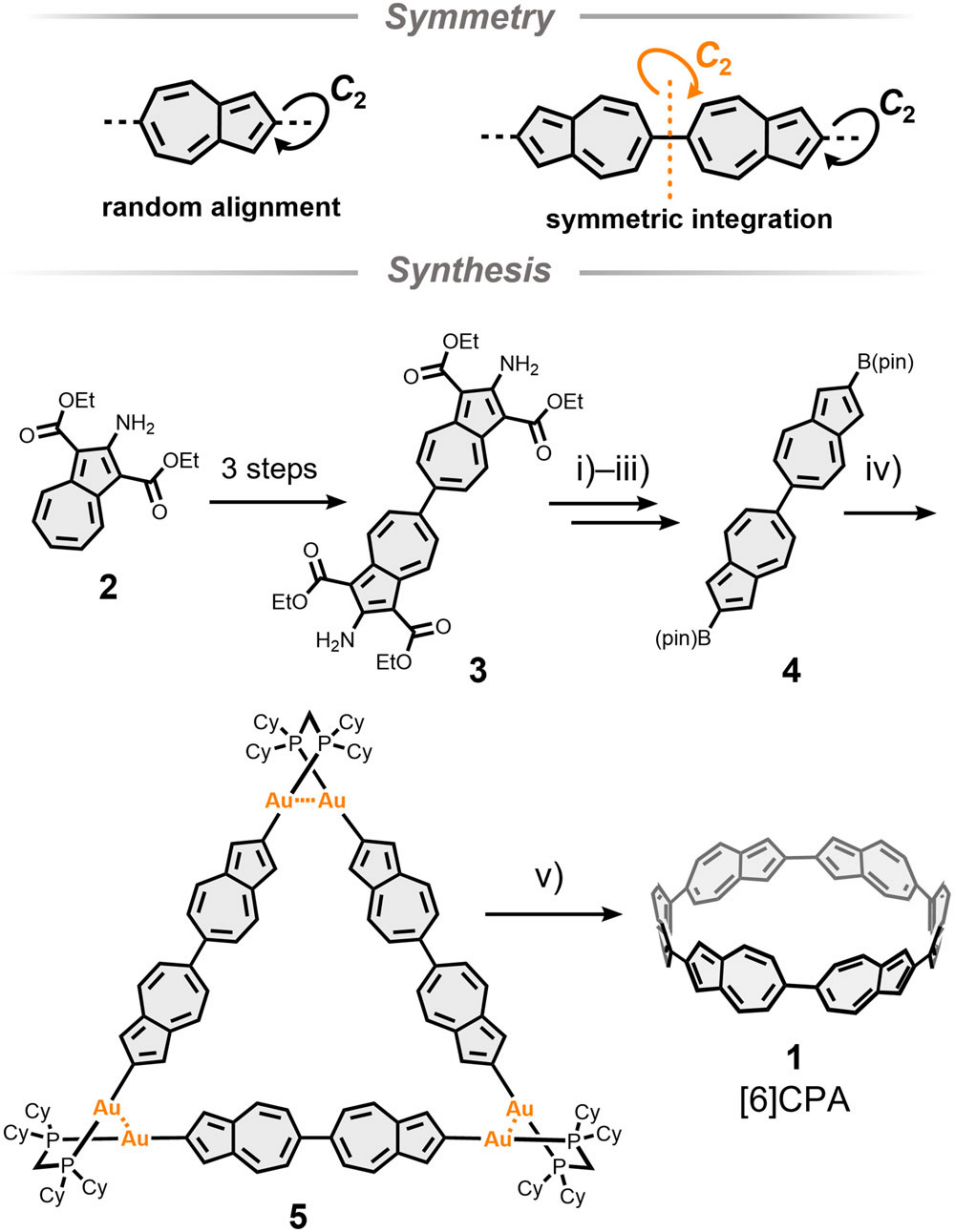
Symmetry considerations and synthesis of [6]CPA **1**. Reagents and conditions: (i) HCl (g), isoamyl nitrite, toluene, 50°C, 16 h, 87%; (ii) H_3_PO_4_, 135°C, 50 min, 99%; (iii) KOAc, bis(pinacolato)diboron, [Pd(dppf)Cl_2_], 1,4‐dioxane, 85°C, 66%; (iv) Cs_2_CO_3_, [Au_2_Cl_2_(dcmp)], toluene/ethanol/water 4:1:1, 50°C, 20 h, 84%; (v) PhICl_2_, DMF, −60°C to 25°C, 40%; dcpm = bis(dicyclohexylphosphino)methane, B(pin)  =  pinacolatoboron, Cy = cyclohexyl. Only one possible stereoisomer of the Au‐macrocycle **5** is depicted. The reaction steps to **3** are described in the Supporting Information: Section S2.

We first set out to identify a suitable linearly substituted monomer for the construction of an all‐azulene macrocycle. Several constitutional isomers are feasible for a six‐membered 2,6‐connected all‐azulene macrocycle; their symmetry and a suggested nomenclature is further discussed in the Supporting Information (Figure S1). Linear symmetry is present in both 2,2′‐ and 6,6′‐biazulene, leading to the formation of a single isomer during macrocyclization (Scheme 1, top). Thus, a 6,6′‐connected azulene dimer borylated at the 2,2′‐positions (**4**) was chosen as the synthetic target [9]. For the construction of the nanohoop, we chose Tsuchido and Osakada's Au‐mediated route, as it has high size‐selectivity and good yields (Scheme 1) [40, 41, 42]. The synthesis of dimeric azulene precursor **3** proceeds from commercial tropolone in seven steps following Nozoe's route (Scheme 1 and see the Supporting Information: Section S2) [43, 44]. The reaction of borylated dimer **4** with [Au_2_Cl_2_(dcmp)] and Cs_2_CO_3_ forms macrocyclic gold complex **5** in 84% yield. Metallacycle **5** is unstable and cannot be stored longer than 2 days at room temperature. The subsequent oxidative chlorination of **5** with PhICl_2_ induces reductive elimination to give [6]cycloparaazulenylene **1** in 40% yield. The most prominent side product of the reductive elimination is 6,6′‐biazulene, which can likely be attributed to protodeauration of macrocycle **5** [45]. Depending on subtle changes of the reaction conditions, 6,6′‐biazulene was frequently the main or only product of the reductive elimination, and the exact reaction and purification conditions required careful fine‐tuning. (for details see Supporting Information: Table S1).

**FIGURE 2:**
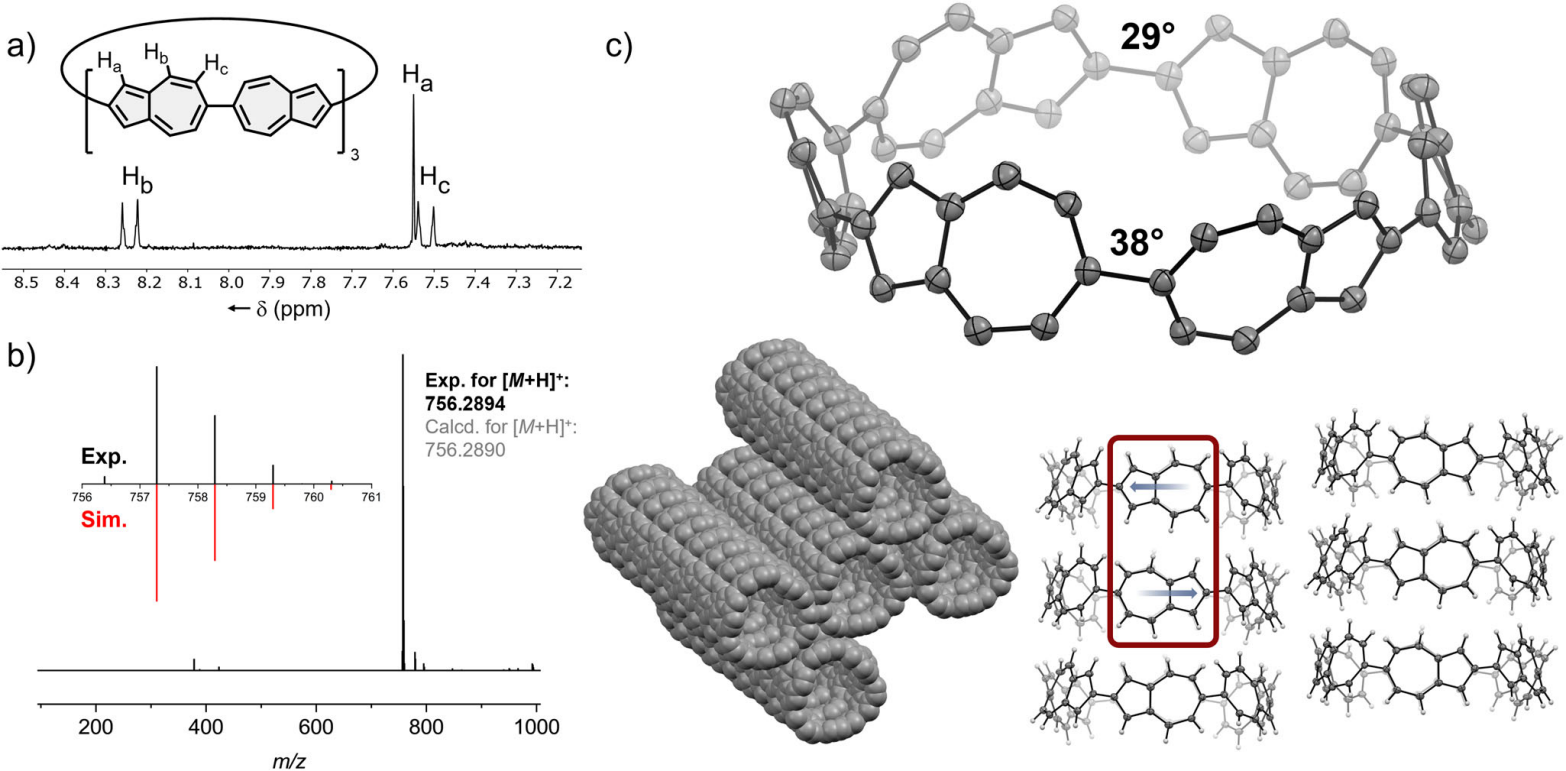
(a) ^1^H NMR spectrum, (b) ESI‐TOF high‐resolution mass spectrum (black) and simulated mass spectrum (red), (c) single‐crystal X‐ray structure (100 K) and packing of **1**, ellipsoids shown at 50% probability; hydrogens are partially omitted for clarity.

Finally, careful purification of the crude mixture on triethylamine‐treated silica gel gave [6]CPA **1** as a dark red solid. A signal of *m*/*z*  =  756.2894 was detected in the high‐resolution ESI‐TOF mass spectrum, corresponding to the [**1**+H]^+^ ion (Figure 2b). The ^1^H NMR spectrum of **1** (Figure 2a) shows a singlet at 7.55 ppm corresponding to the C(1,3) proton of the five‐membered ring (H_a_) and two doublets with *J* = 11.1 Hz at 8.24 and 7.52 ppm, corresponding to the C(4,8) and C(5,7) protons of the seven‐membered ring, respectively (H_b,c_). After purification, **1** can be stored under argon at –20°C for approximately one week, although slow degradation to an NMR‐inactive species begins almost immediately (an unidentifiable brown precipitate forms). Stability varies greatly between the amorphous and crystalline solids (see below). The decomposition of **1** in solution under ambient conditions can be tracked via ^1^H NMR spectroscopy and UV–Vis spectroscopy (see Supporting Information: Section S4.3). Irradiation with 350 nm UV light or treatment with TFA accelerates this decomposition drastically. The absence of new signals in the aromatic region of the ^1^H NMR spectrum indicates a complete degradation of the scaffold, likely initiated by protonation at the 1,3‐position of an azulene subunit. Although [6]CPA contains 60 carbon atoms, we did not detect any trace of C_60_ fullerene in any of its decomposition mixtures. As a guest, C_60_ binds to the central cavity of **1** with an association constant *K*_a_ = 89 M^−1^ in CS_2_ at 25°C, determined by ^1^H NMR isothermal titration (see Supporting Information: Section S6).

The solid‐state structure of macrocycle **1** was determined via single‐crystal X‐ray crystallography (Figure 2c). Slow diffusion of dry, degassed cyclohexane into a dry, degassed dichloromethane solution of **1** in the dark resulted in the growth of dark purple needle‐shaped crystals with a metallic blue sheen. Notably, the optical anisotropy of the single‐crystals could not be determined due to their low transmittance, rendering crystal picking challenging. [6]CPA **1** crystallizes in the highly symmetric trigonal *R* $\bar{3}$ space group, with two molecules being included in the unit cell. The macrocycle has a diameter of 12.8 Å, comparable to that of [9]CPP (12.3 Å) [46], with dihedral angles between the azulene subunits of 28.5° for the 2,2′‐connection and 38.3° for the 6,6′‐connection. The packing structure of **1** is unusual for a pure hydrocarbon nanohoop, forming straight columns along the *c*‐axis of the unit cell. Most CPPs (including similar‐sized [9]CPP), with the exception of [6]CPP and perfluorinated PFCPPs [47, 48], favor herringbone‐like packing to maximize π–π‐interactions [46, 49]. In the solid‐state structure of **1**, each macrocycle in the column is rotated by 60° relative to the next neighbor. This causes very dense packing between the subunits of the columns and rationalizes the unusual tubular packing motif. Additionally, the molecular dipole vectors of the individual azulene subunits are aligned in an antiparallel fashion along the column axes. These columns are offset towards each other, and show weak inter‐column C—H···π edge‐to‐face interactions. The macrocycle cavities contain residual electron density, which can likely be attributed to disordered and partially missing solvent molecules. Unlike the amorphous solid, crystals of **1** were found to be stable for at least two weeks after initial X‐ray diffraction measurement.

**FIGURE 3:**
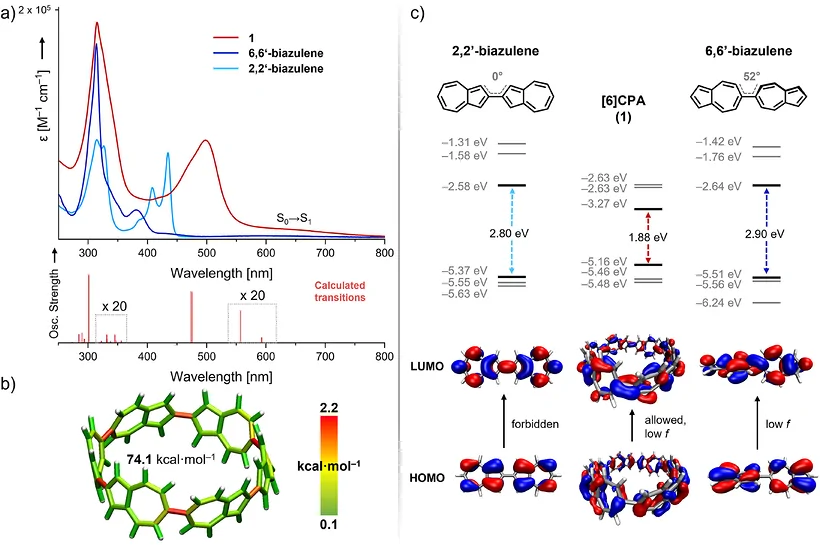
(a) UV–Vis Spectra of 6,6′‐biazulene, 2,2′‐biazulene [50], and **1** in CHCl_3_ and calculated transitions of **1** (CAM‐B3LYP/def2‐TZVP, scrf(CPCM) = chloroform), (b) StrainViz visualization of **1** at the B3LYP/6‐31G(d) level of theory with total and local strain energies (c) calculated orbital energies (B3LYP/def2‐TZVP) and visualized frontier molecular orbitals of 2,2′‐biazulene, 6,6′‐biazulene, and **1** at 0.025 iso value.

To establish a basis for the interpretation of the UV–Vis spectrum of **1**, we analyzed the transitions visible in the absorption spectra of 2,2′‐ and 6,6′‐biazulene in CHCl_3_. These two azulene dimers are building blocks of **1**, in which both the 2,2′‐ and 6,6′‐connectivities are present. Both dimers feature distinct absorption bands of two types: (i) strong, well‐resolved bands in the UV‐ and blue edge of the visible region from 250–450 nm and (ii) a noticeably weaker absorption band in the visible region ranging from 500 to 700 nm (Figure 3a). The conformations of the respective biazulenes must be considered in explaining their absorption spectra; 2,2′‐biazulene has its single minimum energy geometry in a coplanar conformation, while 6,6′biazulene is skewed with a dihedral angle of 52° between the azulene units. The lowest‐energy transition in both cases is a HOMO→LUMO transition, which, as in azulene, has a low oscillator strength due to low spatial overlap of the HOMO and LUMO. In 2,2′‐biazulene, this transition is symmetry‐forbidden. In the higher energy region, further transitions with high MO spatial overlap cause intense absorption bands.

Major differences in the intensities and positions of the absorption bands of the biazulenes can be explained by crossings of the potential energy surfaces of the *S*_3_, *S*_4_, and *S*_5_ states (see Supporting Information: Section S8.4.2, which visualizes the plots of their TD‐DFT energies against the dihedral angle between the azulene units). It is the dihedral angle of the minimum‐energy conformation, rather than the connectivity itself, that determines the structure of the biazulenes' UV–Vis bands. The skewed conformation of 6,6′‐biazulene lies near an intersection of the *S*_3_ and *S*_5_ states. This causes a re‐ordering [51] of orbital contribution and spatial overlap, ultimately resulting in an intensity depletion in the first strong absorption band (420–470 nm) in 6,6′‐biazulene, as compared to the coplanar conformation of 2,2′‐biazulene.

Analysis of the absorption spectrum of [6]CPA **1** in CHCl_3_ is complicated by a lack of robust orbital assignment, as only the very first transition around 650 nm can be confidently assigned to a single pair of MOs, namely a HOMO→LUMO transition. A group of weak transitions corresponds to the shoulder band centered around 600 nm and is tailing past 800 nm. Commonly, the first transition in even‐numbered CPPs is symmetry‐forbidden [52]. However, unlike other even‐numbered CPPs, [6]CPA is not centrosymmetric, and the low intensity of its first band does not originate from symmetry‐forbidden transitions. The same is true for the next six transitions, rationalizing the broad structure of the absorption band of **1**. The intense absorption band at 500 nm is the first transition with efficient orbital overlap and is analogous to the third transition in 2,2′‐biazulene, with a red‐shift being caused by extended *π*‐conjugation in the macrocycle. Since the dihedral angles of the biazulenylene subunits of macrocycle **1** do not lie near the geometry of the crossing point of *S*_3_ and *S*_5_ states in 6,6′‐biazulene, no intensity depletion is observed in this first high orbital overlap band, explaining the relative similarity in the absorption spectra of **1** and 2,2′‐biazulene (see Supporting Information: Section S8.4.2). Unlike azulene, which fluoresces from the *S*_2_ state (*Φ*_f_  =  0.20 in cyclohexane) [53], **1** is non‐emissive in CHCl_3_, with no indication of anti‐Kasha emission, due to an efficient internal conversion of **1**.

The photophysical features of **1** were modeled via DFT (structure calculated at the B3LYP/def2‐TZVP level of theory, TD‐DFT: CAM‐B3LYP/def2‐TZVP, C‐PCM using CHCl_3_ as implicit solvent, Figure 3a). The HOMO–LUMO gap of **1** is extremely narrow at 1.88 eV, compared to 6,6′‐ and 2,2′‐biazulene (2.90 and 2.80 eV, respectively), pristine azulene (3.36 eV), and even 2,6‐azulene homopolymers (2.27 eV) [9]. The HOMO+1 and HOMO+2 (as well as the LUMO–1 and LUMO–2) are nearly degenerate, as is common for [*n*]CPPs [52]. The FMOs are distributed evenly along the perimeter of the macrocycle. Further, the strain of **1** was determined via StrainViz (Figure 3b) [54]. The total calculated strain energy is 74.1 kcal mol^−1^, which is higher than that of similar‐sized [9]CPP at 64.2 kcal mol^−1^ [54]. The contributions of bond and angle strain are minor, while the dihedral strain is the major contribution at 62.3 kcal mol^−1^ in **1**. The StrainViz calculation confirmed that the 6,6′‐connectivity of the azulenes is more strained than the 2,2′‐connectivity.

[6]CPA is a 60 *π*‐electron (4*n*) conjugated macrocycle comprising six individual 10 *π*‐electron conjugated (4*n*+2) azulene subunits, thereby suggesting global Hückel antiaromaticity of **1**, but local Hückel aromaticity of its azulene subunits [55]. Thus, we investigated the electronic structure of **1** in the context of its Hückel (anti)aromaticity, using a combination of magnetic (NICS [56], AICD) [57] and delocalization (MCI [58], FLU [59], EDDB) [60, 61] indices, as well as resonance/isomerization stabilization energies (ISE) [62], calculated by Kohn–Sham density functional theory (DFT) approach. Calculations were performed using the global minimum‐energy geometry, determined by a CREST conformer search and subsequently optimized at B3LYP/def2‐TZVP level of theory [63, 64]. Full method description and computational results are included in the Supporting Information: Section S8.

**FIGURE 4:**
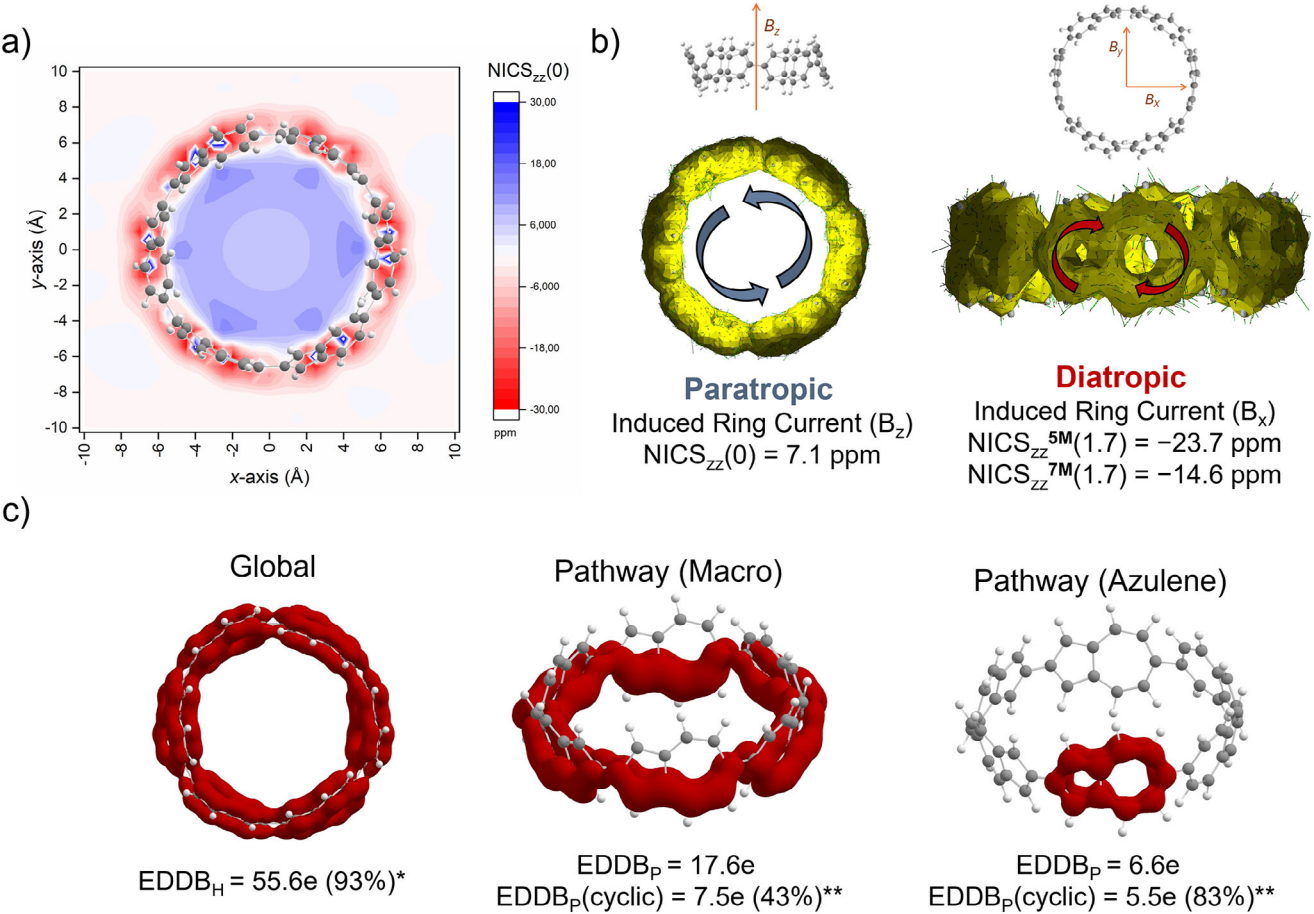
(a) NICS_zz_(0) heatmap, calculated at B3LYP/6‐311+G** level of theory, using a 10 × 10 Å grid with 0.5 Å steps. (b) AICD plots, calculated by applying an external magnetic field in the *z*‐axis (B_z_) and the *x*‐axis (B_x_), alongside the NICS_zz_(0) at the center of the macrocycle, and NICS_zz_(1.7) of 5‐membered (5 M) and 7‐membered rings (7 M) rings of an azulene subunit, calculated at B3LYP/6‐311+G** level of theory. (c) EDDB plots, showing the global electron delocalization through heavy atoms of [6]CPA (EDDB_H_, iso = 0.0265), electron delocalization along the perimeter (EDDB_P_) of the [6]CPA macrocycle (iso = 0.0125), and along the perimeter of a single azulene subunit (iso = 0.0225), calculated at B3LYP/def2‐TZVP level of theory; *EDDB_H_ delocalization efficiency is referenced to 60 *π*‐electrons; **Fraction of circularly delocalized electrons is referenced to the calculated EDDB_P_ electron count.

As revealed by NICS and AICD calculations, under the influence of an external magnetic field, **1** exhibits a weak globally induced paratropic current (positive NICS_zz_ values, 7.1 ppm) originating from the macrocycle's perimeter, and strong local diatropic induced currents (negative NICS values), originating from the individual azulene subunits (Figure 4a,b). One might assume that the magnetic indices qualify the entirety of the macrocycle's 60 *π*‐electron perimeter as antiaromatic and its individual 10 *π*‐electron azulene subunits as aromatic, in line with Hückel's rule. Yet, while these extrinsic (anti)aromaticity criteria reflect the *π*‐electron counts of the delocalization circuits in **1**, they fail to ascribe their influence on net delocalization and net resonance stabilization [65].

Indeed, **1** is efficiently delocalized in the absence of an external magnetic field, as revealed by EDDB calculations (Figure 4c). Closer examination of individual delocalization circuits reveals that the azulene subunits are responsible for most of the delocalized electron density. Along the global perimeter, the delocalization is comparatively less efficient. Although delocalization is not uniform, the delocalization efficiency in the macrocycle's perimeter circuit is similarly large to other macrocyclic molecules, which are considered aromatic [66, 67]. Thus, contrary to the magnetic indices, the delocalization indices show that **1** is globally well delocalized, with no hints of antiaromaticity.

To resolve the discrepancy between the values of the magnetic indices and the delocalization indices, we quantified the resonance stabilization in **1** by ISE. The calculated ISE values, except for those at the bridging positions, were negative, ranging from −11.4 to −21.7 kcal mol^−1^, thereby indicating resonance stabilization in **1**. In the bridging positions (i.e., positions 2 and 6 of the parent azulene), where isomerization caused the most drastic geometry change and, consequently, was responsible for comparatively larger strain relief, the calculated ISE values were slightly less negative (shorter 2,2′ bridging bond) or null (longer 6,6′ bridge). These negative ISE values convincingly demonstrate that the 60 (4*n*) *π*‐electrons of **1** do not lead to net resonance destabilization, which would indicate antiaromaticity.

Owing to its conjugated 60 *π*‐electrons, applying an external magnetic field to **1** induces a paratropic ring current, suggesting that the molecule should be considered Hückel antiaromatic based on extrinsic (anti)aromaticity criteria [68]. In contrast, intrinsic (anti)aromaticity criteria, electron delocalization, and ISE indicate net‐stabilization by the aromatic 10 *π*‐electron azulene subunits and global *π*‐delocalization. Thus, the extrinsically indicated global antiaromaticity of **1**, observed only under the influence of an external magnetic field, is vanishingly small and does not significantly influence the intrinsic properties of **1**.

## 3 Conclusion

In conclusion, we present a conjugated macrocycle consisting entirely of 2,6‐connected azulene subunits, featuring alternating 2,2′‐ and 6,6′‐connected azulenes in the backbone. Macrocycle **1** represents the first cyclic architecture consisting solely of azulene subunits [69]. It exhibits an unusual tubular packing motif in the crystalline state. Due to strain and delocalization through the backbone, a remarkably low‐lying LUMO causes a very narrow HOMO–LUMO gap compared to other azulene derivatives. The overall electronic structure of **1** can best be described by the inherent aromaticity of its 10 π‐electron subunits, though a weak paratropic current is induced along the 60 *π*‐electron macrocyclic perimeter upon application of an external magnetic field. The unique characteristics of the reported all‐azulene nanohoop establish a new basis for the development of optically and electronically tunable carbon nanostructures.

## Author Contributions

**Clara Douglas**: conceptualization, methodology, investigation, validation, formal analysis, visualization, writing – original draft, writing – review and editing. **Josefine Sprachmann**: conceptualization, methodology, investigation, validation, formal analysis, visualization, writing – review and editing. **David Dunlop**: methodology, software, data curation, investigation, formal analysis, visualization, writing – original draft, writing – review and editing. **Joël Schlecht**: methodology, validation, formal analysis. **Jörg Neudörfl**: methodology, formal analysis. **Tommy Wachsmuth**: methodology, investigation, validation, formal analysis. **Julian Frost**: validation, investigation. **Tomáš Slanina**: investigation, validation, formal analysis, supervision, funding acquisition, project administration, writing – review and editing. **Oliver Dumele**: conceptualization, methodology, data curation, investigation, validation, formal analysis, supervision, funding acquisition, project administration, resources, writing – review and editing.

## Funding

This work was supported by Fonds der Chemischen Industrie and BMFTR.

## Conflicts of Interest

The authors declare no conflicts of interest.

## Supporting information



The authors have cited additional references within the Supporting Information [70, 71, 72, 73, 74, 75, 76, 77, 78, 79, 80, 81, 82, 83, 84, 85, 86, 87, 88, 89, 90, 91].
**Supporting File 1**: anie73394‐sup‐0001‐Data1.zip

**Supporting File 2**: anie73394‐sup‐0002‐SuppMat.pdf.

## Data Availability

The data that support the findings of this study are available in the Supporting Information of this article.
