# Supplementary material for: Cycloazulenylene
Source: Angew Chem Int Ed Engl. 2026 Jul 16;65(37):e2204400. doi: 10.1002/anie.2204400 (PMC13548887; doi:10.1002/anie.2204400)
Supplement: Supplementary file 1 [file ANIE-65-e2204400-s001.pdf]

Supporting Information

Cycloazulenylene

Clara Douglas,[a] Josefine Sprachmann,[b] David Dunlop,[c],[d] Joel Schlecht,[a] Jörg Neudörfl,[a] Tommy Wachsmuth,[b] Julian Frost,[b] Tomáš Slanina,[c],* Oliver Dumele[a],*

[a]Department of Chemistry, University of Cologne, Greinstrasse 4, 50939 Cologne, Germany

[b]Department of Chemistry, Humboldt Universität zu Berlin, Brook-Taylor-Strasse 2, 12489 Berlin, Germany

[c]Institute of Organic Chemistry and Biochemistry of the Czech Academy of Sciences, Flemingovo náměstí 542/2, Prague 6, 160 00, Czech Republic

[d]Department of Inorganic Chemistry, Faculty of Science, Charles University in Prague, Hlavova 2030, Prague 2, 128 40, Czech Republic

*Corresponding author,

e-mail: O.D.: odumele@uni-koeln.de, T.S.: tomas.slanina@uochb.cas.cz

Table of Contents

S1 General Remarks 3
S2 Synthetic Procedures 5
S3 NMR Spectra...15
S4 Purification and Degradation Studies of [6]CPA...23
S4.1 Condition Screening Reductive Elimination 23
S4.2 Experimental UV-Vis spectra of [6]CPA..24
S4.3 Degradation experiments on [6]CPA 26
S5 Spectroelectrochemistry 31
S6 NMR Titration Studies...33
S7 Selected mass spectra 35
S8 Computations 37
S8.1 Computational Methods 37
S8.2 Computational Results [6]CPA 38
S8.2.1 Geometry of [6]CPA 38
S8.2.2 MOs and TD-DFT...41
S8.2.3 Strain Calculations 44
S8.2.4 Aromaticity Analysis of [6]CPA 45
S8.2.5 Geometric and Electronic Delocalization Indices (HOMA, FLU, MCI)..............45
S8.2.6 Nucleus-Independent Chemical Shifts (NICS & NICS Heatmaps)...................46
S8.2.7 Anisotropy of Induced Current Density (AICD Plots) 49
S8.2.8 Isomerization Stabilization Energy (ISE)..50
S8.2.9 Electron Delocalized Density in Bonds (EDDB) 52
S8.3 Computational Results on 2,2’-Biazulene and 6,6’-Biazulene 56
S8.3.1 Geometry of 2,2’- and 6,6’-Biazulene..56
S8.3.2 TD-DFT Results 59
S8.3.3 Geometric and Electronic Delocalization Indices (HOMA, FLU, MCI)..............62
S8.3.4 Magnetic (Shielding) Indices (NICS)..63
S8.3.5 Anisotropic Induced Current Density (AICD Plots) 64
S8.3.6 Isomerization stabilization energy (ISE)...64
S8.4 Extended Discussion TD-DFT 67
S8.4.1 Azulene 67
S8.4.2 2,2’-Biazulene and 6,6’-Biazulene 68
S8.4.3 [6]CPA..74
S8.5 Atomic Coordinates of Calculated Structures 74
S9 Crystallographic Data...80
S10 References..87

S1 General Remarks

Reagents (Acros, AlfaAesar, BLDpharm, Sigma-Aldrich, TCI, and Thermo Scientific) were purchased as reagent grade and used without further purification, unless otherwise specified. Polyphosphoric acid (ca. 84% as phosphorus pentoxide) and N,N'-dimethylformamide (anhydrous, 99.8%, packaged under Argon in AcroSeal bottles, used in the reductive elimination of **1**) were purchased from Thermo Scientific. Au-complex Au_2(dcpm)Cl_2 (dcpm = bis(dicyclohexylphosphino)methane) was synthesized according to the literature.[1]

Solvents for synthesis were dried using a Pure Solv Micro Solvent Purification System from Innovative Technology and stored over molecular sieves 3–4 Å.

Glassware All non-aqueous reactions were performed in oven-dried glassware and under an Argon atmosphere.

Automated medium pressure column chromatography (MPLC) was performed on a Teledyne ISCO CombiFlashRf 300 system with 200 mL/min max flow, 200 psi, equipped with integrated ELSD and 200–800 nm UV-vis variable wavelength detector.

High-performance liquid chromatography (HPLC) was run on a Waters 600 HPLC System equipped with a waters 600 solvent pump, a Waters 600 controller, and a waters 996 photodiode array detector.

UPLC–HR–ESI–MS was performed using a Waters UPLC ACQUITY H-Class PLUS with a Waters Alliance System (Waters Separations Module 2695, Waters Diode Array Detector 996) coupled to a Xevo G3 QTof mass analyzer with ESI or API ion source.

Mass spectrometry (MS) and **high-resolution mass spectrometry (HRMS)** were recorded on an Agilent 6546 quadrupole/time-of-flight (LC/Q-TOF) instrument, equipped with electrospray ionization (ESI) and atmospheric-pressure chemical ionization (APCI) and a 1290 Infinity II (UPLC) direct infusion inlet system or a MALDI-TOF Autoflex Max (Bruker Daltonik, Bremen), 355 nm faser, 4000 shot accumulated on 2 different spots, reflector modus, Matrix DCTB (*trans*-2-[3-(4-*tert*-butylphenyl)-2-methyl-2-propenylidene]) 20 mg mL^{-1} in $CHCl_3$ + 1 mg potassium trifluoroacetate in 350 µL matrix solution pre-mixed with 20 µL sample solution (approximately 0.1 mg mL^{-1} in $CHCl_3$), 1 µL droplet deposited of premixed solution on stainless steel target recording using FlexControl software (Bruker).

Thin layer chromatography (TLC) was conducted on aluminum sheets coated with SiO_2–60 F_{254} obtained from Merck; visualization with a UV lamp (254 or 366 nm).

Nuclear magnetic resonance (NMR) spectra were recorded using a Bruker Avance III HD 300 (300 MHz for ^1H and 75 MHz for ^{13}C), Bruker Avance II 400 (400 MHz for ^1H and 101 MHz for ^{13}C), Bruker Avance III HD 500 (500 MHz for ^1H and 126 MHz for ^{13}C), and a Bruker Avance III HD 800 (800 MHz for ^1H and 201 MHz for ^{13}C) with Cryo TXO at 25 °C and are reported as follows: chemical shift (δ) in ppm (multiplicity, coupling constant J in

Hz, number of protons; assignment). The residual deuterated solvent was used as the internal reference ($CDCl_3$: δ_H = 7.26 ppm); ($CDCl_3$: δ_C = 77.16 ppm); (CD_2Cl_2: δ_H = 5.32 ppm); (CD_2Cl_2: δ_C = 53.84 ppm); The resonance multiplicity is described as s (singlet), d (doublet), t (triplet), q (quartet), m (multiplet), and br. (broad).

Single-crystal X-ray data were measured with a BRUKER D8 VENTURE area detector with Mo-Kα (λ = 0.71 Å) or Cu-Kα (λ = 1.54 Å) radiation. Multi-scan Absorbance corrections implemented in SADABS were applied to the data. The structures were solved by intrinsic phasing method (SHELXT-2013)[2] and refined by full matrix least square procedures based on F2 with all measured reflections (SHELXL-2014) in the graphical user interface (OLEX2)[3] with anisotropic temperature factors for all non-hydrogen atoms. All hydrogen atoms were added geometrically and refined by using a riding model.

Ultraviolet–Visible (UV-vis) absorbance spectroscopy was performed on Agilent Cary 60 instruments connected to a cryostat from Unisoku Scientific Instruments (temperature accuracy ±0.1 K) in 10 × 10 mm quartz cuvettes with 3 mL volume. Weighing of small quantities was performed on a Sartorius ME5 analytical microbalance.

Steady state fluorescence and phosphorescence were measured with a Jasco FP-8300 Fluorescence Spectrometer equipped with a 150 W xenon lamp or an Edinburgh Instruments FS5 Spectrofluorometer.

Spectroelectrochemistry was performed in a SEC-C Thin Layer Quartz Glass Spectroelectrochemical cell with a 1.0 mm optical path length, with platinum mesh electrode as a working electrode, counter electrode: platinum wire, reference electrode: non aqueous reference electrode Ag/Ag^+ (0.01 M $AgNO_3$ in 0.1 M nBu_4ClO_4 in acetonitrile), ALS Co., Ltd (Tokyo, Japan) spectrometer: Metrohm ASM80120 with ALS80112 light source Potentiostat: PGSTAT 204, Deutsche Metrohm GmbH & Co. KG (Filderstadt, Germany) and software: NOVA 2.1. Scan rate for all measurements dE/dt = 10–100 mV s^{-1}.

S2 Synthetic Procedures

Diethyl 6-bromo-2-aminoazulene-1,3-dicarboxylate (2)[4]

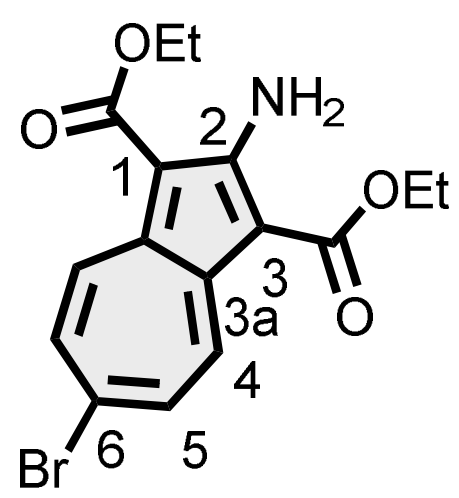

Adapted from a published procedure,[5] a solution of diethyl-2-aminoazulene-1,3-dicarboxylate (3.00 g, 10.4 mmol, 1.0 equiv.) in dichloromethane (80 mL) was cooled to 0 °C. To this, a solution of bromine (0.59 mL, 11.5 mmol, 1.1 equiv.) in dichloromethane (20 mL) was added dropwise over 30 min. The reaction mixture was allowed to return to room temperature and was stirred at 25 °C for 3 d. The reaction was quenched with saturated aqueous Na_2SO_3 solution (40 mL) and extracted with dichloromethane (3 × 50 mL). The combined organic phases were dried over $MgSO_4$, filtered, and the solvent was removed in vacuo. The crude product was recrystallized from toluene/ethanol 1:1 (100 mL), washed with methanol, and dried under high vacuum to obtain **2** (3.36 g, 88%) as a crystalline brown solid.

The analytics are in accordance with the literature.[5]

^{1}H NMR (500 MHz, $CDCl_3$, 25 °C): δ = 8.85 (dd, J = 10.4, 1.2 Hz, 2H, H–C(4,8)), 7.85–7.76 (m, 4H, H–C(5,7), NH_2), 4.46 (q, J = 7.1 Hz, 4H, CH_2), 1.47 ppm (t, J = 7.1 Hz, 6H, CH_3);

^{13}C NMR (126 MHz, $CDCl_3$, 25 °C): δ = 166.4 (C=O), 162.5 (C(2)), 144.2 (C(3a,84)), 135.5 (C(5,7)), 129.7 (C(4,8)), 128.6 (C(6)), 101.1 (C(1,3)), 60.2 (CH_2), 14.8 ppm (CH_3).

Note: Recrystallization is essential to remove an impurity that leads to side product formation (**S1**, see section 8 for crystal structure) in the next step. The synthesis of azulene-precursor of **2** via the Nozoe-route proceeds from 2-tosyl tropolone,[4] and we propose that an NMR-silent sulfur-containing impurity causes the formation of **S1** when **2** is not purified before Pd-catalyzed transformation.

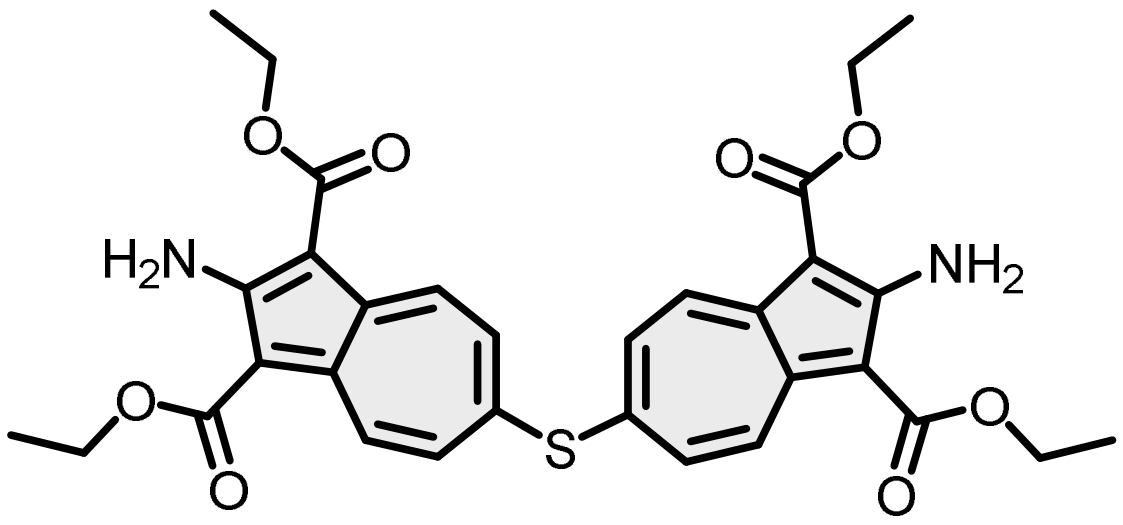

Figure S1. Side product **S1**, which is observed during Pd-catalyzed transformations of **2** which has not been recrystallized.

Diethyl 2-amino-6-(4,4,5,5-tetramethyl-1,3,2-dioxaborolan-2-yl)azulene-1,3-dicarboxylate (2a)[6]

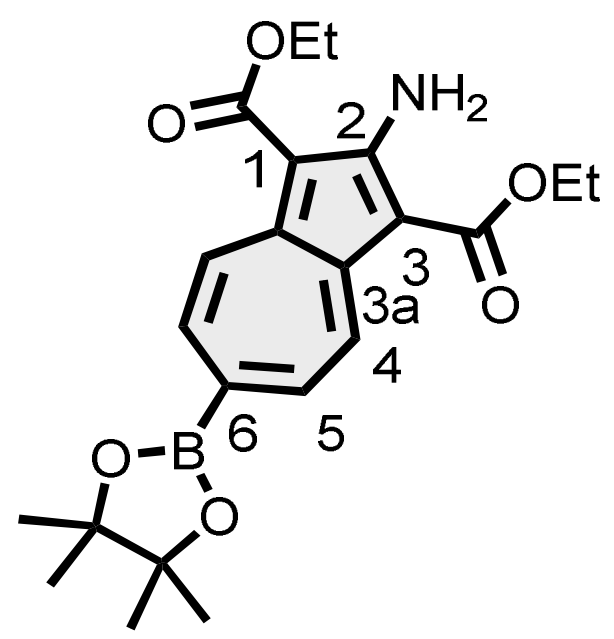

Adapted from a published procedure,[5] KOAc (3.52 mg, 35.9 mmol, 3.0 equiv.) was flame-dried in a Schlenk flask under vacuum. 6-Bromo diethyl 2-aminoazulene-1,3-dicarboxylate (**2**, 3.52 g, 12.0 mmol, 1.0 equiv.), B_2pin_2 (3.34 g, 13.2 mmol, 1.1 equiv.), and [Pd(1,1'-bis(diphenylphosphino)ferrocene)Cl_2·2 CH_2Cl_2] (977 mg, 1.20 mmol, 10 mol%) were added and dissolved in DMSO (40 mL). The mixture was degassed by bubbling Ar for 15 min and then heated to 85 °C for 18 h. The mixture was diluted with water (50 mL) and extracted with dichloromethane (3 x 200 mL). The combined organic phases were washed with water, dried over Mg_2SO_4, filtered, and the solvent was removed in vacuo. Column chromatography (SiO_2, cyclohexane/ethyl acetate 4:1) yielded **2a** (3.80 g, 83%) as an orange solid. The analytics are in accordance with the literature.[5]

$\boldsymbol{R}_\mathbf{f}$ = 0.30 (SiO_2, cyclohexane/ethyl acetate 4:1)

^{1}H NMR (400 MHz, $CDCl_3$, 25 °C): δ = 9.08 (d, J = 10.9 Hz, 2H, H–C(4,8)), 8.05 (d, J = 10.9 Hz, 2H, H–C(5,7)), 7.93 (s, 2H, NH_2), 4.45 (q, J = 7.1 Hz, 4H, CH_2), 1.47 (t, J = 7.1 Hz, 6H, CH_3 of ethyl), 1.38 ppm (s, 12H, CH_3 of dioxaborolanyl);

^{13}C NMR (100 MHz, $CDCl_3$, 25 °C): δ = 166.8 (C=O), 163.7 (C(2)), 147.5 (C(3a,8a)), 138.8 (C(4,8)), 130.4 (C(5,7)), 99.9 (C(1,3)), 84.7 (C(4',5') of dioxaborolanyl), 59.9 (CH_2), 25.0 (C–OB), 14.7 ppm (CH_3); C(6) is hidden by the noise due to quadrupolar coupling.

Tetraethyl 2,2'-diamino-6,6'-biazulenyl-1,1',3,3'-tetracarboxylate (3)[6]

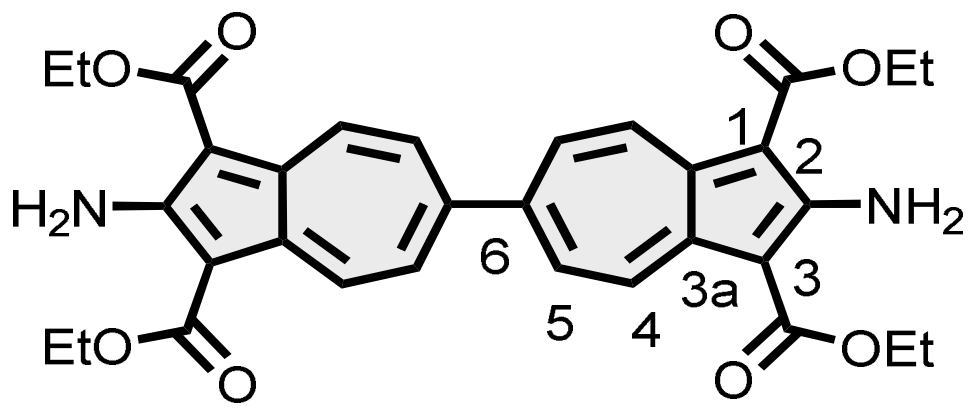

A solution of diethyl 6-bromo-2-aminoazulene-1,3-dicarboxylate (**2**, 3.08 g, 8.42 mmol, 1.0 equiv.), **2a** (3.48 g, 8.42 mmol, 1.0 equiv.), and potassium carbonate (3.49 g, 25.3 mmol, 3.0 equiv.) in toluene/water/ethanol 10:1:1 (280 mL) was degassed by bubbling Ar for 20 min. Palladium tetrakis(triphenylphosphine) (681 mg, 589 µmol, 7 mol%) was added and the reaction mixture was heated to 105 °C for 4 h. After cooling to 25 °C, the solid product was filtered off, washed with ethanol (100 mL), and dried under high vacuum to obtain **3** (3.61 g, 75%) as a brick red solid. The analytical characterization is in accordance with the literature.[7]

^{1}H NMR (500 MHz, $CDCl_3$, 25 °C): δ = 9.17 (d, J = 11.5 Hz, 4H, H–C(4,4',8,8')), 7.87 (s, 4H, NH_2), 7.78 (dd, J = 11.1, 1.1 Hz, 4H, H–C(5,5',7,7')), 4.49 (q, J = 7.1 Hz, 8H, CH_2), 1.50 ppm (t, J = 7.1 Hz, 12H, CH_3);

^{13}C NMR (126 MHz, $CDCl_3$, 25 °C): δ = 166.6 (C=O), 162.9 (C(2,2')), 148.5 (C(6,6') or C(3a,3a',8a,8a')), 145.0 (C(6,6') or C(3a,3a',8a,8a')), 133.5 (C(5,5',7,7')), 130.6 (C(4,4',8,8')), 100.5 (C(1,1',3,3')), 60.1 (CH_2), 14.8 ppm (CH_3).

Tetraethyl 2,2'-dichloro-6,6'-biazulenyl-1,1',3,3'-tetracarboxylate (3a)[8]

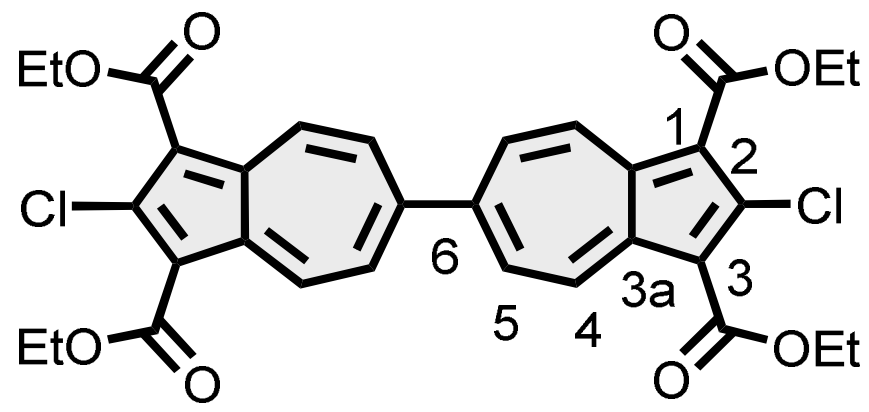

HCl gas was bubbled through a solution of 2,2'-amino-1,1',3,3'-tetraethoxycarbonyl-6,6'-biazulenyl (**3**, 1.50 g, 2.62 mmol, 1.0 equiv.) in dry toluene (40 mL) at 25° for 1 h. The red suspension was treated dropwise with isoamyl nitrite (1.55 mL, 11.5 mmol, 4.4 equiv.) and the mixture was stirred at 50 °C for 16 h. The mixture was diluted with CH_2Cl_2 (100 mL), extracted with sat. aq. $NaHCO_3$ (100 mL) and water (100 mL), dried over $MgSO_4$, and concentrated under vacuum. The crude product was suspended in cyclohexane (100 mL) and filtered off to obtain **3a** (1.44 g, 90%) as a purple solid. The analytics are in accordance with the literature.[8]

^{1}H NMR (500 MHz, $CDCl_3$, 25 °C): δ = 9.60 (d, J = 11.1 Hz, 2H, H–C(4,4',8,8')), 7.93 (d, J = 11.1 Hz, 2H, H–C(5,5',7,7')), 4.52 (q, J = 7.1 Hz, 4H, CH_2), 1.50 ppm (t, J = 7.1 Hz, 6H, CH_3);

^{13}C NMR (125 MHz, $CDCl_3$, 25 °C): δ = 164.2 (C=O), 154.9 (C(6,6') or C(3a,3a',8a,8a')), 144.7 (C(2,2')), 140.8 (C(6,6') or C(3a,3a',8a,8a')), 137.2 (C(4,4',8,8')), 131.7 (C(5,5',7,7')), 116.4 (C(1,1',3,3')), 60.9 (CH_2), 14.4 ppm (CH_3).

2,2’-Dichloro-6,6’-biazulenyl (3b)[8]

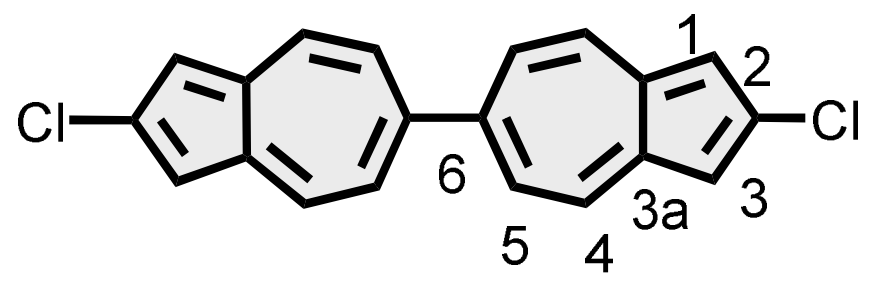

Adapted with changes from a published procedure,[8] polyphosphoric acid (30 mL) was preheated to 135 °C and tetracarboxylate **3a** (300 mg, 0.49 mmol) was added in one portion. The mixture was stirred for 50 min and subsequently poured onto ice (100 mL). After the ice melted, the mixture was extracted with $CHCl_3$ (8 x 150 mL), dried over $MgSO_4$, and concentrated under vacuum to yield **3b** (156 mg, 99%) as a dark brown solid. The analytics are in accordance with the literature.[8]

^{1}H NMR (500 MHz, $CDCl_3$, 25 °C): δ = 8.29 (d, J = 10.6 Hz, 4H, H–C(4,4’,8,8’)), 7.47 (d, J = 10.5 Hz, 4H, H–C(5,5’,7,7’)), 7.30 ppm (s, 4H, H–C(1;1’,3,3’));

^{13}C NMR (125 MHz, $CDCl_3$, 25 °C): δ = 154.0 (C(6,6’)), 141.0 (C(2,2’)), 138.7 (C(3a,3a’,8a,8a’)), 134.6 (C(4,4’,8,8’)), 126.0 (C(5,5’,7,7’)), 116.9 ppm (C(1,1’,3,3’)).

2,2'-Bis(4,4,5,5-tetramethyl-1,3,2-dioxaborolan-2-yl)-6,6'-biazulenyl (4)[8]

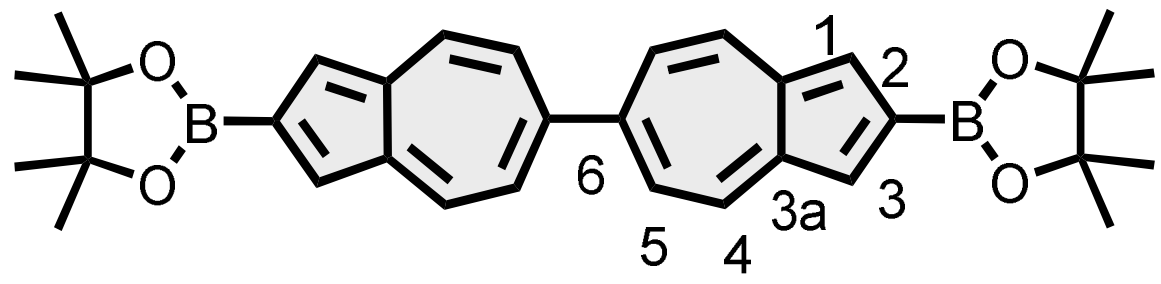

KOAc (641 mg, 11.50 mmol, 12 equiv.) was flame-dried in a Schlenk flask under vacuum. 2,2'-Dichloro-6,6'-biazulenyl (**3b**, 340 mg, 0.974 mmol, 1.0 equiv.), B_2pin_2 (641 mg, 2.339 mmol, 2.4 equiv.) and [Pd(1,1'-bis(diphenylphosphino)ferrocene)Cl_2·2 CH_2Cl_2] (172 mg, 0.195 mmol, 20 mol%), were added and dissolved in 1,4-dioxane (26 mL). The mixture was degassed by bubbling Ar for 10 min and then heated to 100 °C for 20 h. After cooling to room temperature, the mixture was diluted with CH_2Cl_2 (50 mL) and water (50 mL) and extracted using CH_2Cl_2 (3 x 40 mL). The combined organic phases were dried using $MgSO_4$ and concentrated under vacuum. The crude mixture was purified by a silica plug (using $B(OH)_3$ impregnated silica,[9] diameter 4 cm, length 5 cm) and CH_2Cl_2 as eluent giving **4** (350 mg, 66%) as a green solid. The analytics are in accordance with the literature.[8]

^{1}H NMR (4z00 MHz, $CDCl_3$, 25 °C): δ = 8.39 (d, J = 10.6 Hz, 4H, H–C(4,4',8,8')), 7.80 (s, 4H, H–C(1,1',3,3')), 7.33 (d, J = 10.5 Hz, 4H, H–C(5,5',7,7')), 1.42 ppm (s, 24H, CH_3);

^{13}C NMR (126 MHz, $CDCl_3$, 25 °C): δ = 156.0 (C(3a,3a',8a,8a')), 139.8 (C(6,6')), 137.3 (C(4,4',8,8')), 126.1 (C(1,1',3,3')), 124.1 (C(5,5',7,7')), 84.0 (C–O), 25.1 (CH_3) ppm, C–B is hidden by the noise due to quadrupolar coupling;

HR-EI-MS m/z (%): 506.2804 (100, $[M+H]^+$, calcd. for $C_{32}H_{36}O_4B_2^+$: 506.2794).

Three-fold Au-biazulenyl-macrocycle ((±)-5)

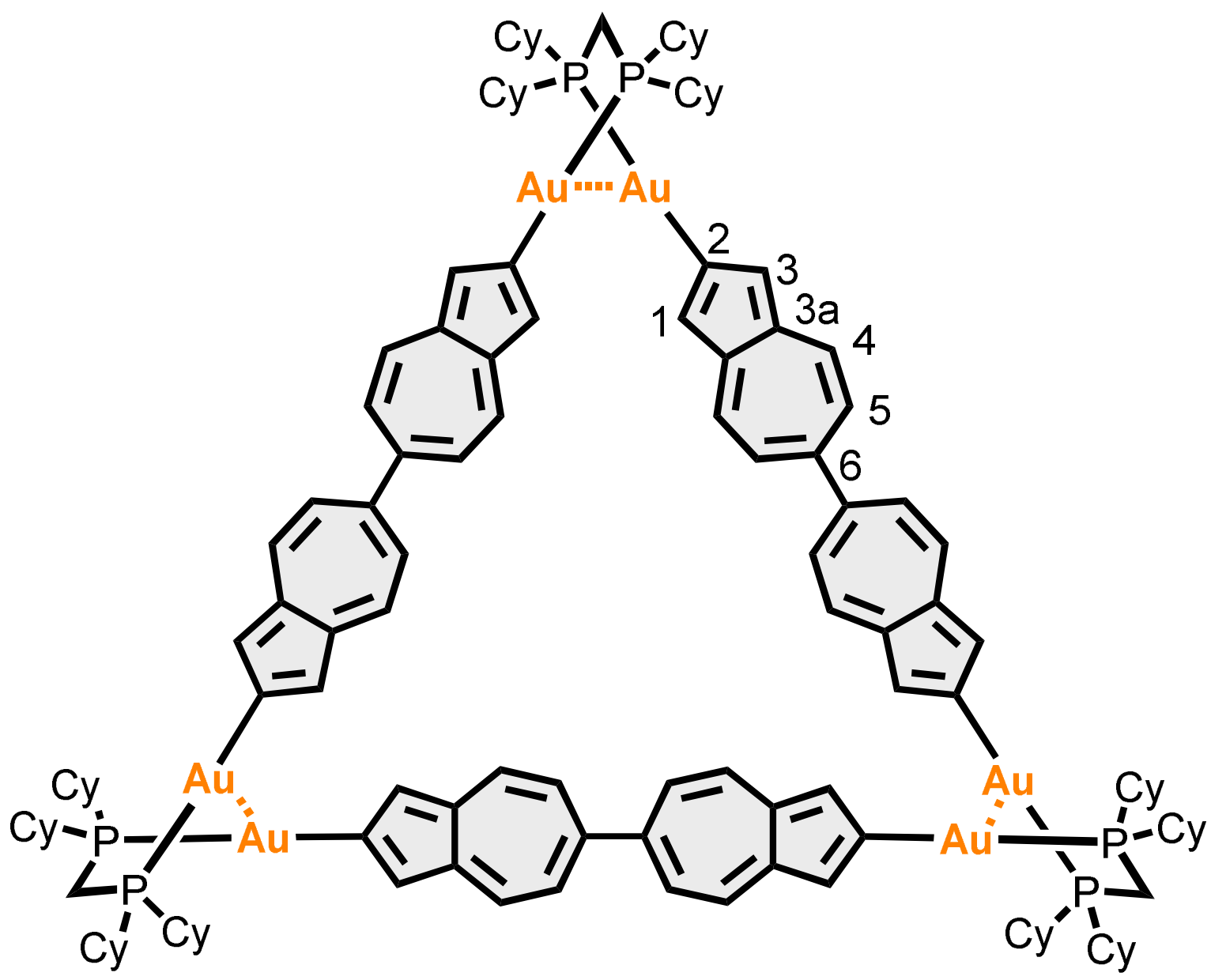

(only one stereoisomer is depicted above; Cy = cyclohexyl)

A 10 mL-pressure tube was filled with [Au_2(dcpm)Cl_2] (55.2 mg, 63.2 µmol, 1.0 equiv.), bis(boronic ester) **4** (32.0 mg, 63.2 µmol, 1.0 equiv.), Cs_2CO_3 (124 mg, 379 µmol, 6.0 equiv.), and toluene/EtOH/H_2O 4:1:1 (3.36 mL). The mixture was degassed using a stream of Ar and stirred at 50 °C for 21 h. After cooling to 25 °C, the reaction mixture was diluted with CH_2Cl_2 (10 mL) and water (10 mL), and extracted with CH_2Cl_2 (3 x 10 mL). The organic phase was dried over $MgSO_4$ and the solvent was evaporated yielding crude **5** (56 mg, 84%) as a brown solid. The crude mixture was used in the next reaction step on the same day and stored under Ar.

^{1}H NMR (300 MHz, $CDCl_3$, 25 °C): δ = 8.11 (d, J = 10.2 Hz, 12H, (H–C(4,4',8,8' or 5,5',7,7')), 7.59 (d, J = 10.2 Hz, 12H, H–C(4,4',8,8' or 5,5',7,7')), 7.40 (s, 12H, H–C(1,1',3,3')), 3.32–1.30 ppm (br. m, 126H, cyclohexyl);

^{31}P NMR (121 MHz, $CDCl_3$, 25 °C): δ = 48.18 ppm;

^{13}C NMR was not obtained due to limited solubility and stability. Despite multiple attempts, MALDI-TOF MS and ESI-TOF MS were unsuccessful.

[3]Cyclo(6,6’-biazulene-2,2’-diyl) (1)

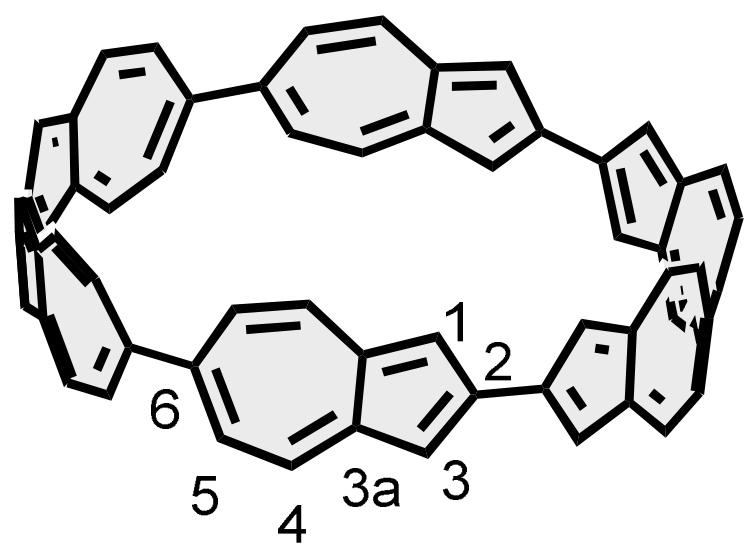

A solution of gold-macrocycle **5** (28 mg, 8.8 μmol) in dry DMF (9 mL) was degassed using a stream of Ar and then cooled to –65 °C using a bath of acetone and dry ice. A degassed solution of freshly synthesized $PhICl_2$ (7.3 mg, 27 μmol, 3.0 equiv.) in dry DMF (3 mL) was added dropwise over the course of 30 min, causing a color change from brown to dark red. The reaction mixture was stirred for another 30 min at –65 °C, after which the cooling bath was removed and the mixture was allowed to warm to 25 °C and stirred at that temperature for 24 h. Afterwards, the solvent was evaporated at 30 °C using high vacuum (ca. 10^{-2} bar) and a cooling trap. The resulting dark red solid was stored strictly under Ar. The crude mixture was purified using column chromatography (SiO_2, cyclohexane/CH_2Cl_2 100:0→80:20 with 1% NEt_3), yielding **1** (3.0 mg, 40%) as a dark red solid.

***R*f** = 0.25 (SiO_2, cyclohexane/CH_2Cl_2 1:4 with 1% NEt_3);

^{1}H NMR (300 MHz, CD_2Cl_2, 25 °C): δ = 8.24 (d, *J* = 11.1 Hz, 12H, H–C(4,8)), 7.52 (d, *J* = 11.1 Hz, 12H, H–C(5,7)), 7.55 ppm (s, 12H, H–C(1,3));

^{13}C NMR (201 MHz, CD_2Cl_2, 25 °C): δ = 149.8 (C(6)), 145.8 (C(2)), 141.7 (C(3a,8a)), 135.2 (C(4,8)), 125.3 (C(5,7)), 118.0 ppm (C(1,3));

HR-ESI-TOF-MS *m*/*z* (%): 756.2894 (100, $[M+H]^+$, calcd. for $C_{60}H_{37}^+$: 756.2890);

MALDI-MS *m*/*z* (%): 755.95 (100, $[M]^+$, calcd. for $C_{60}H_{36}^+$: 756.2812);

SC-XRD was obtained, see the according section below.

Note on the nomenclature and symmetry of **1**

Considering all possible constitutional isomers of a six-membered azulenylene macrocycle connected along its 2,6 positions ([6]CPA), there are seven symmetry classes to consider (if rotation, reflection and global inversion of the macrocycle are allowed). In this case, every azulenyl subunit can be described simply as one of two orientations, visualized here as an arrow and named "a" or "b". Only few of these isomers are synthetically feasible using a threefold Au-macrocyclization, two of which are shown below: The hypothetical dipole-aligned "head-to-tail" [6]CPA, *ht*[6]CPA, and the "alternating" (head-to-head and tail-to-tail connected) [6]CPA, *a*[6]CPA, presented in this work and herein referred to simply as [6]CPA.

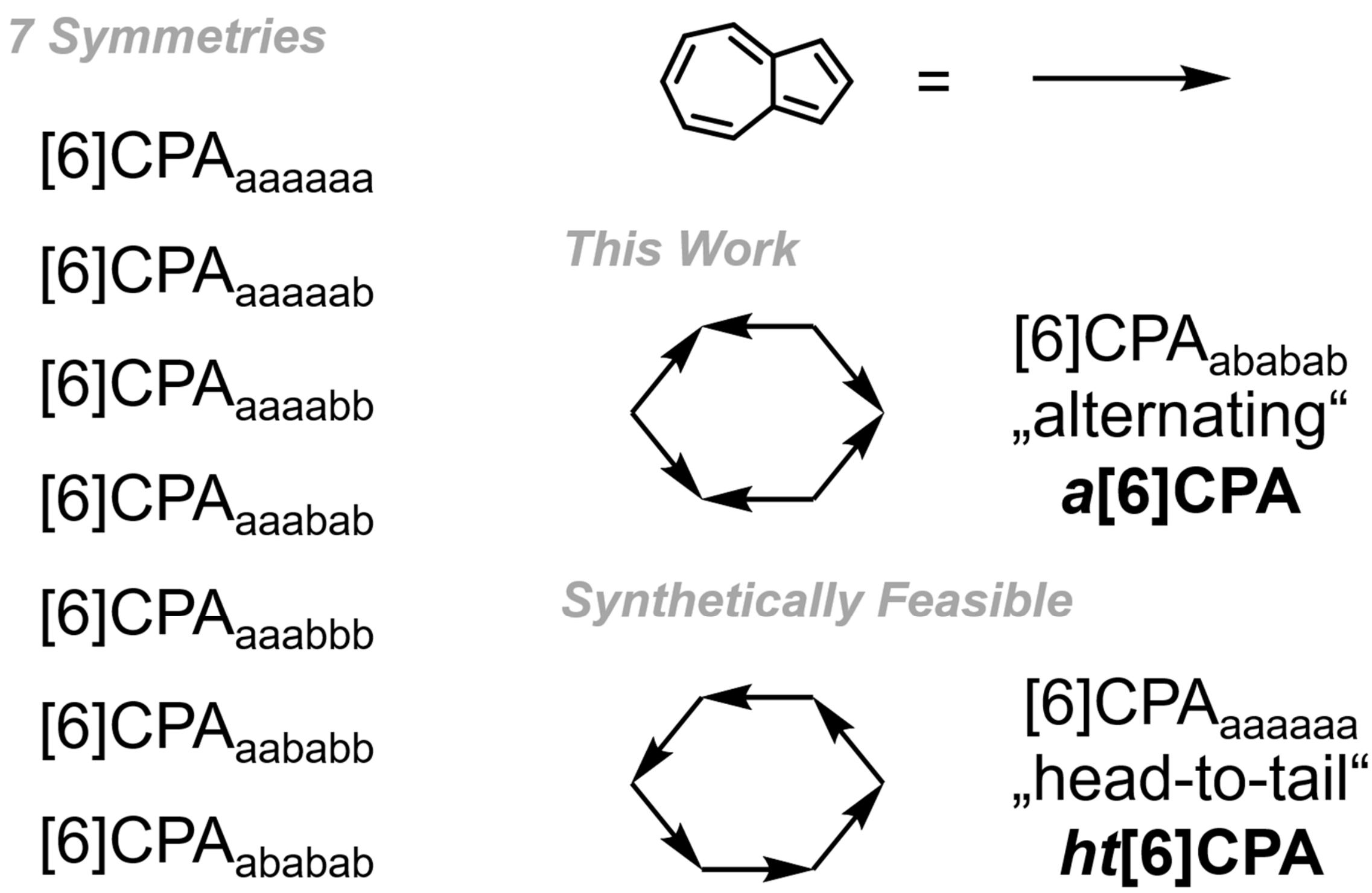

Figure S2. Symmetry overview of possible [6]CPA isomers.

S3 NMR Spectra

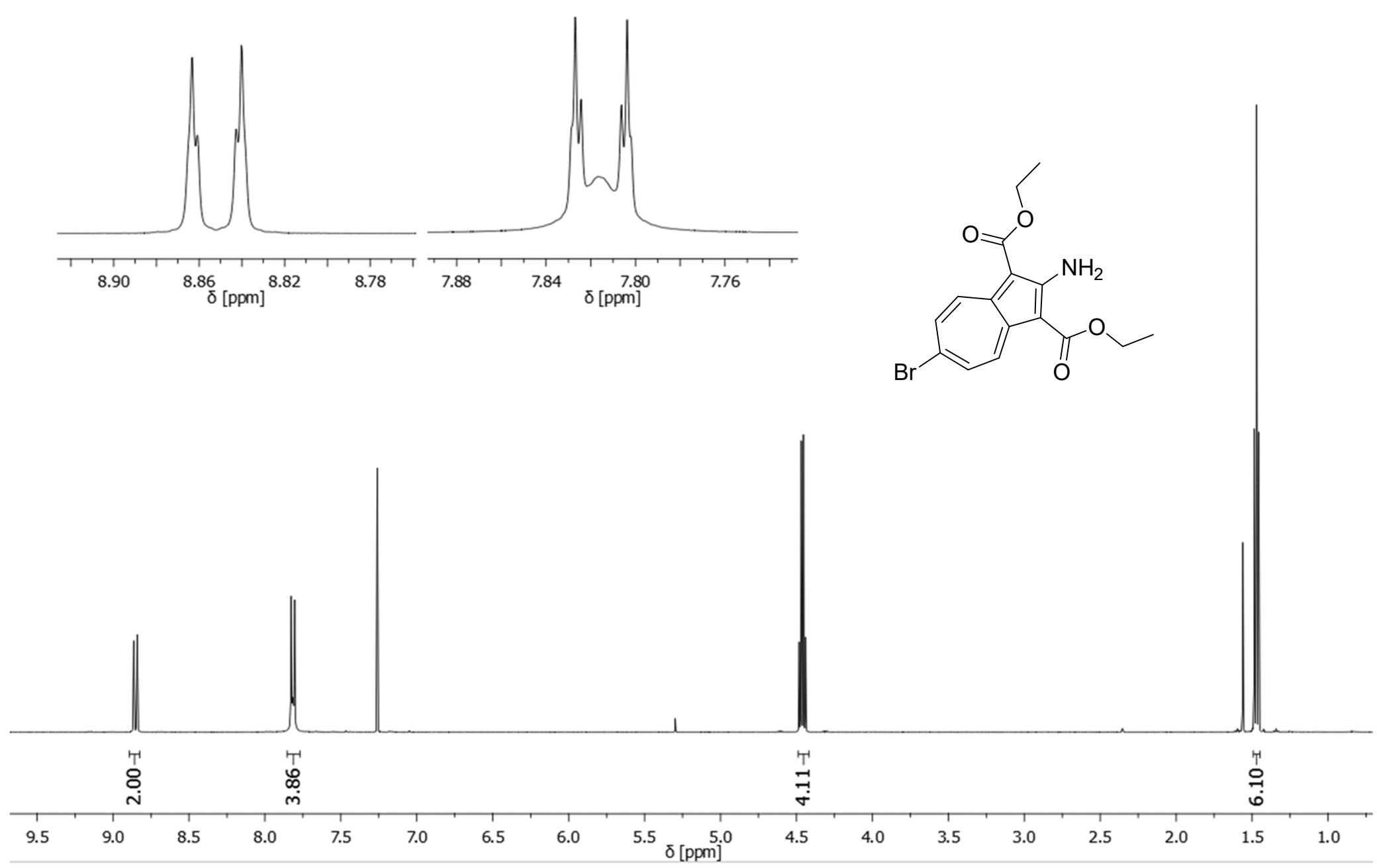

Figure S3. ^{1}H NMR spectrum (500 MHz, $CDCl_3$, 25 °C) of **2**.

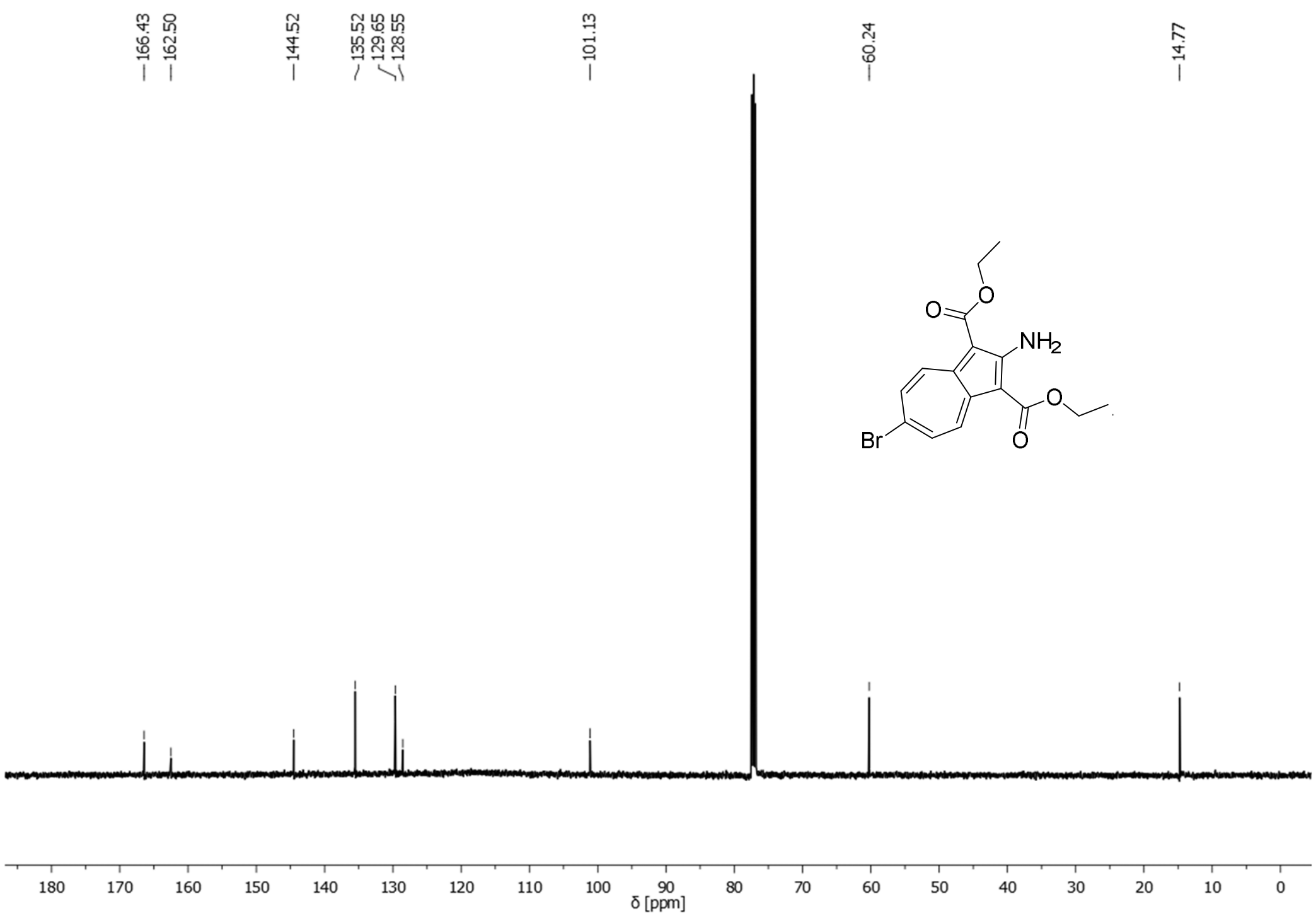

Figure S4. ^{13}C NMR spectrum (126 MHz, $CDCl_3$, 25 °C) of **2**.

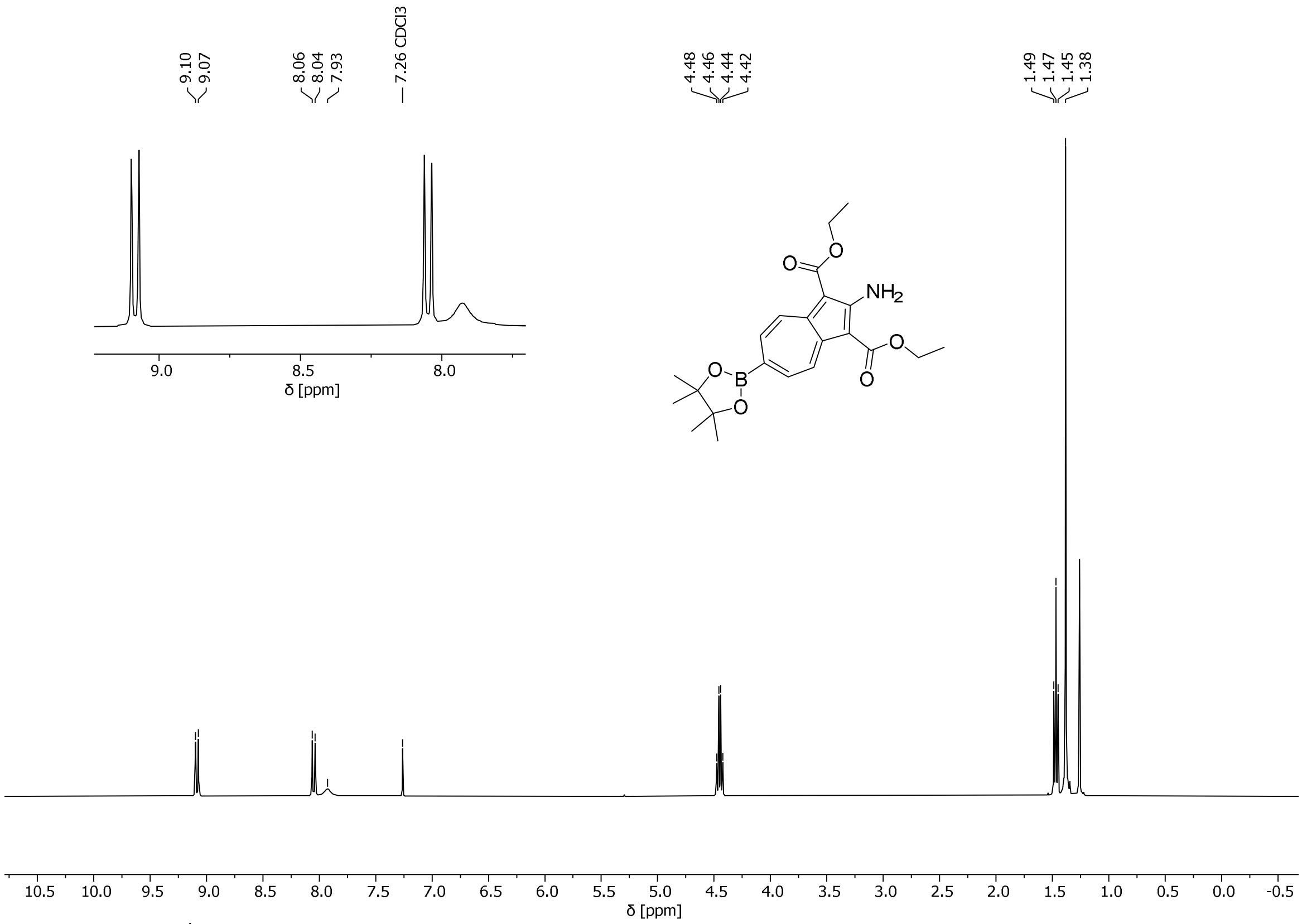

Figure S5. ^{1}H NMR spectrum (400 MHz, $CDCl_3$, 25 °C) of **2a**.

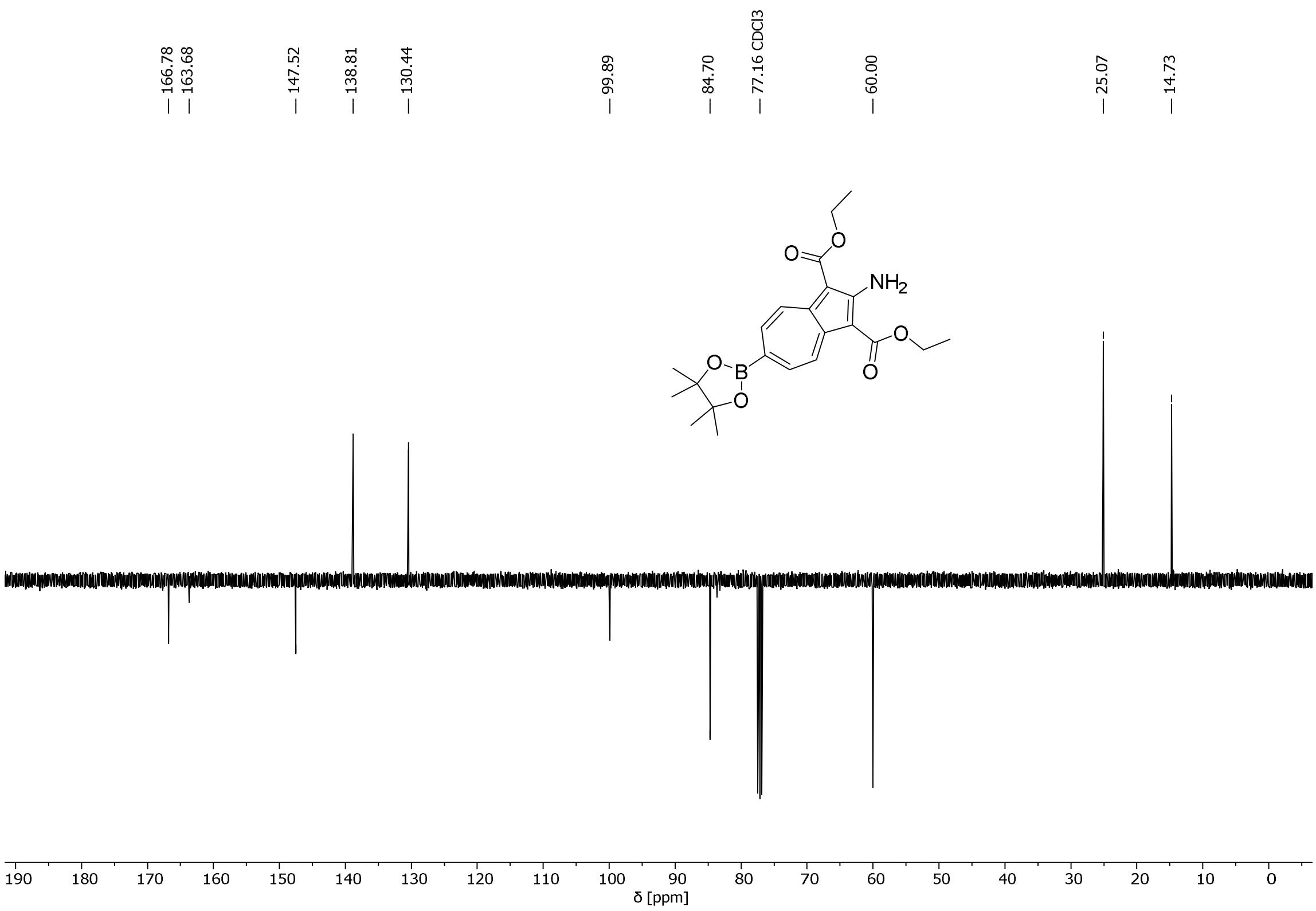

Figure S6. ^{13}C NMR APT spectrum (100 MHz, $CDCl_3$, 25 °C) of **2a.**

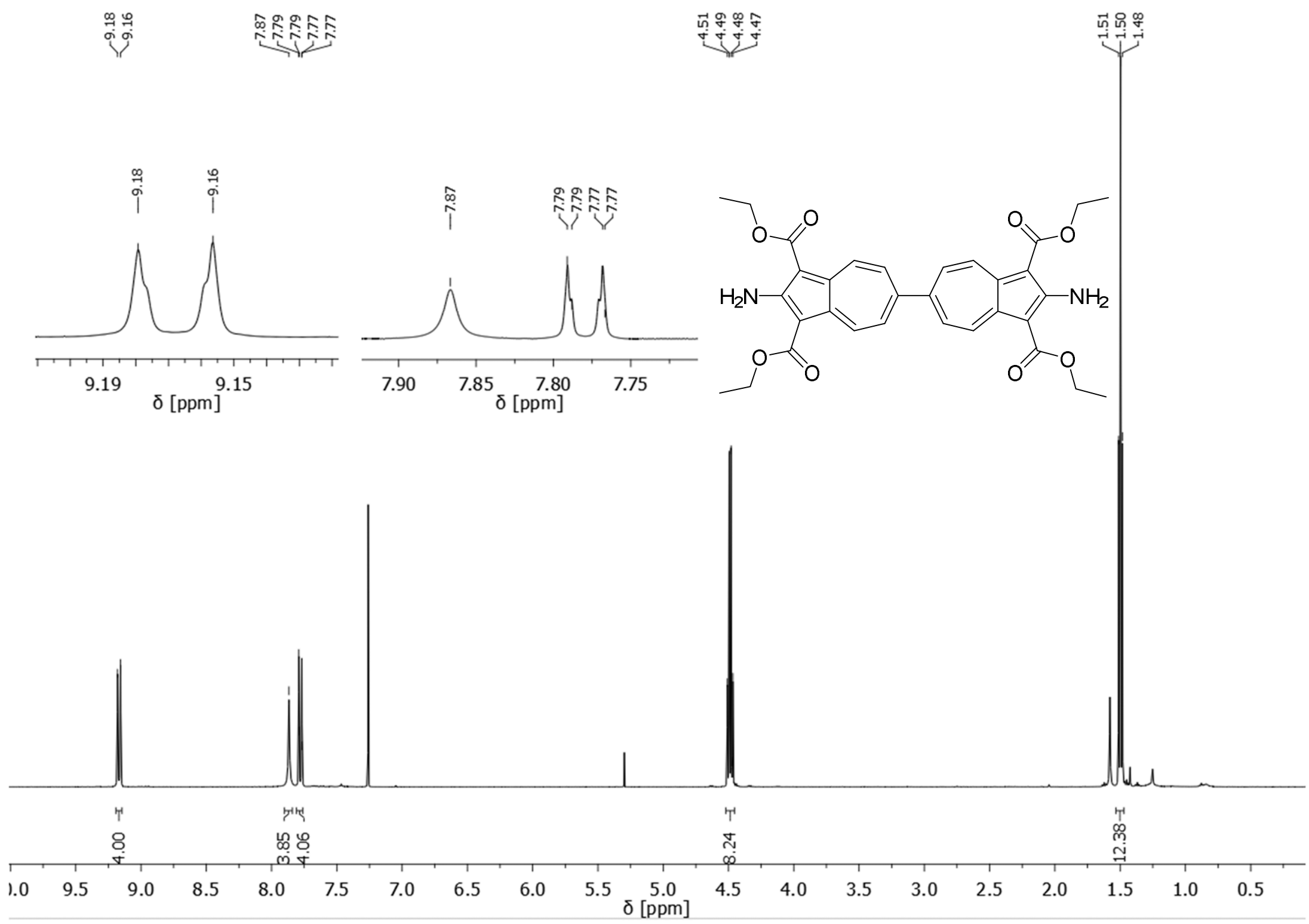

Figure S7. ^{1}H NMR spectrum (500 MHz, $CDCl_3$, 25 °C) of **3**.

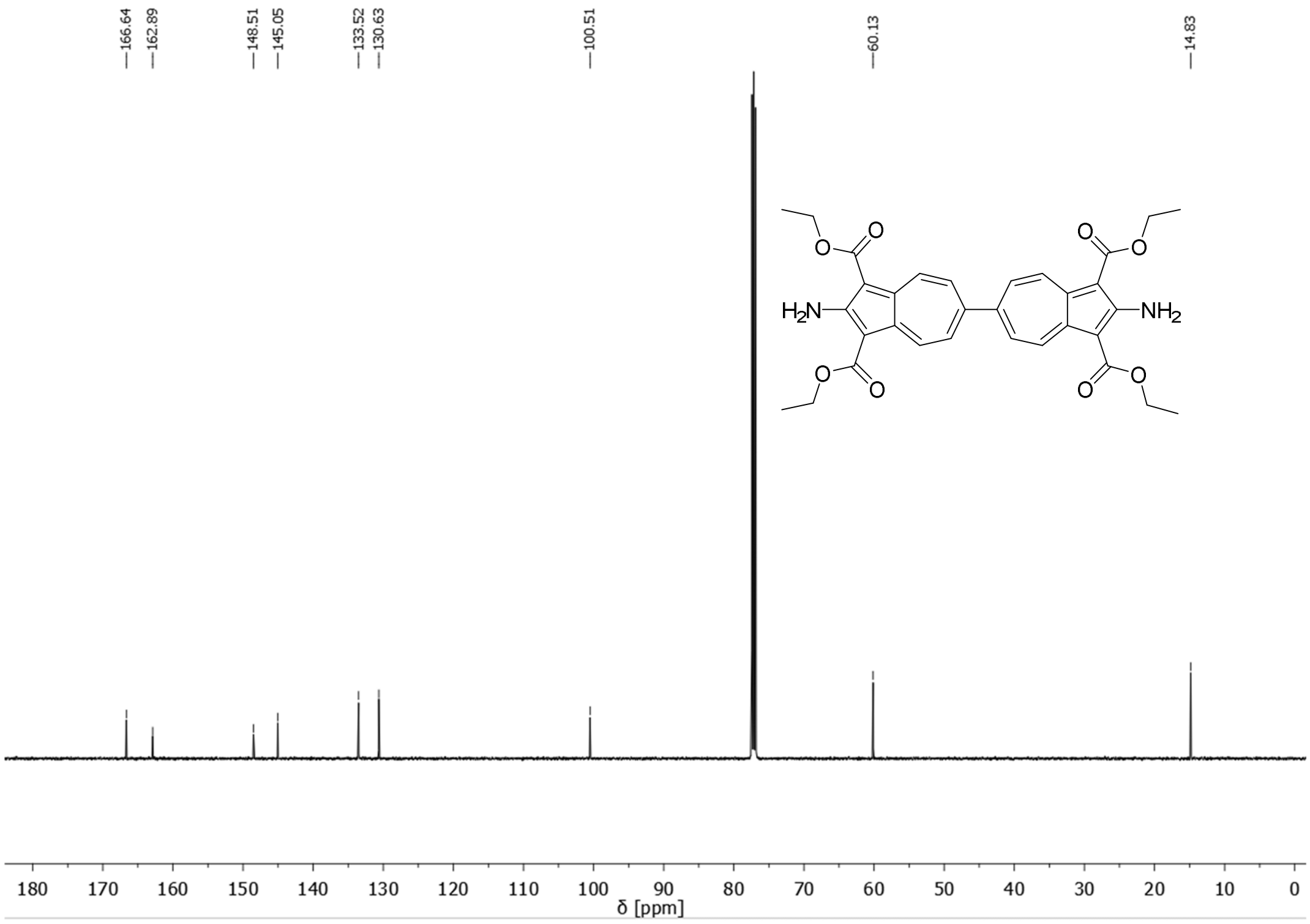

Figure S8. ^{13}C NMR spectrum (126 MHz, $CDCl_3$, 25 °C) of **3**.

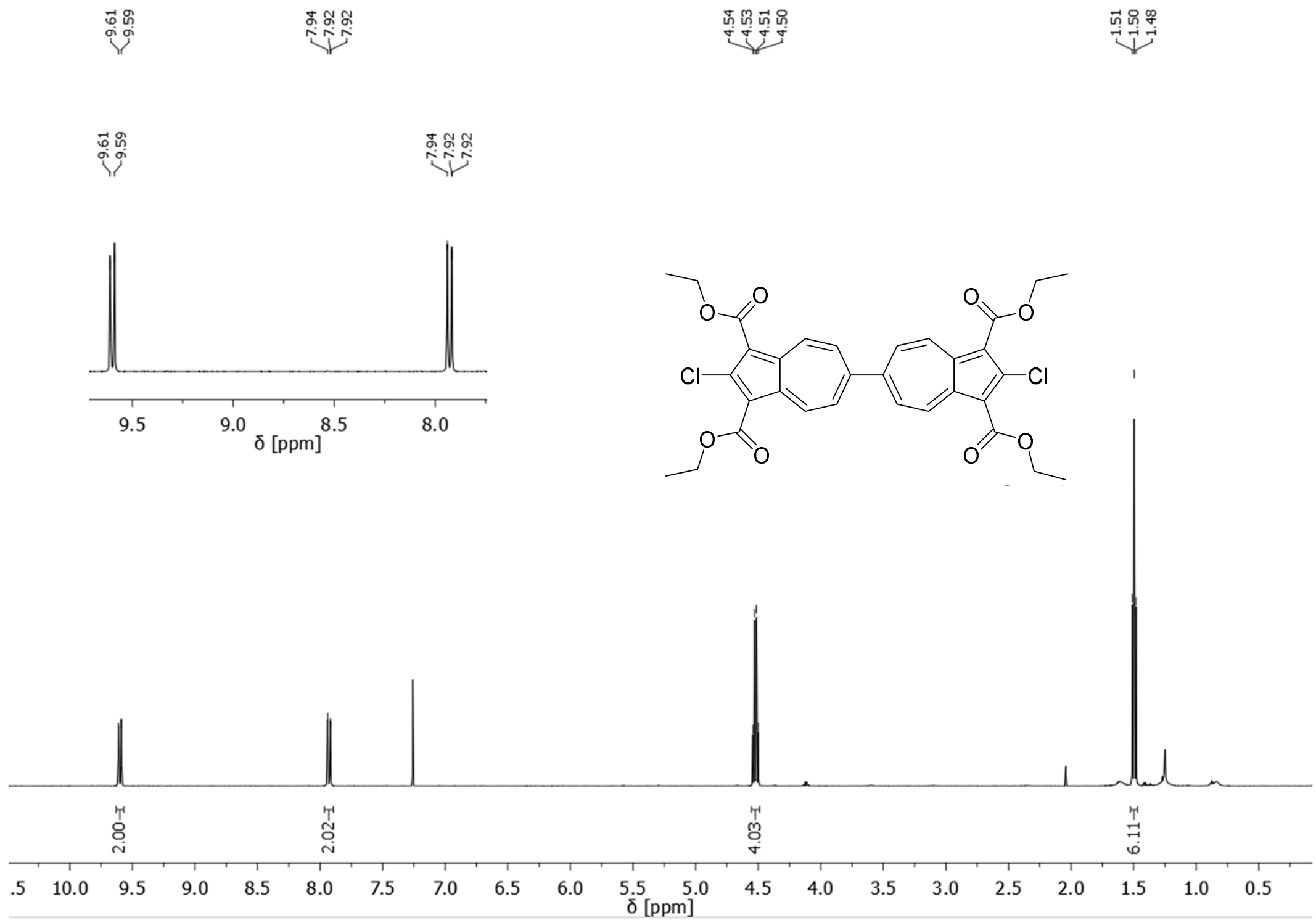

Figure S9. ^{1}H NMR spectrum (500 MHz, $CDCl_3$, 25 °C) of **3a**.

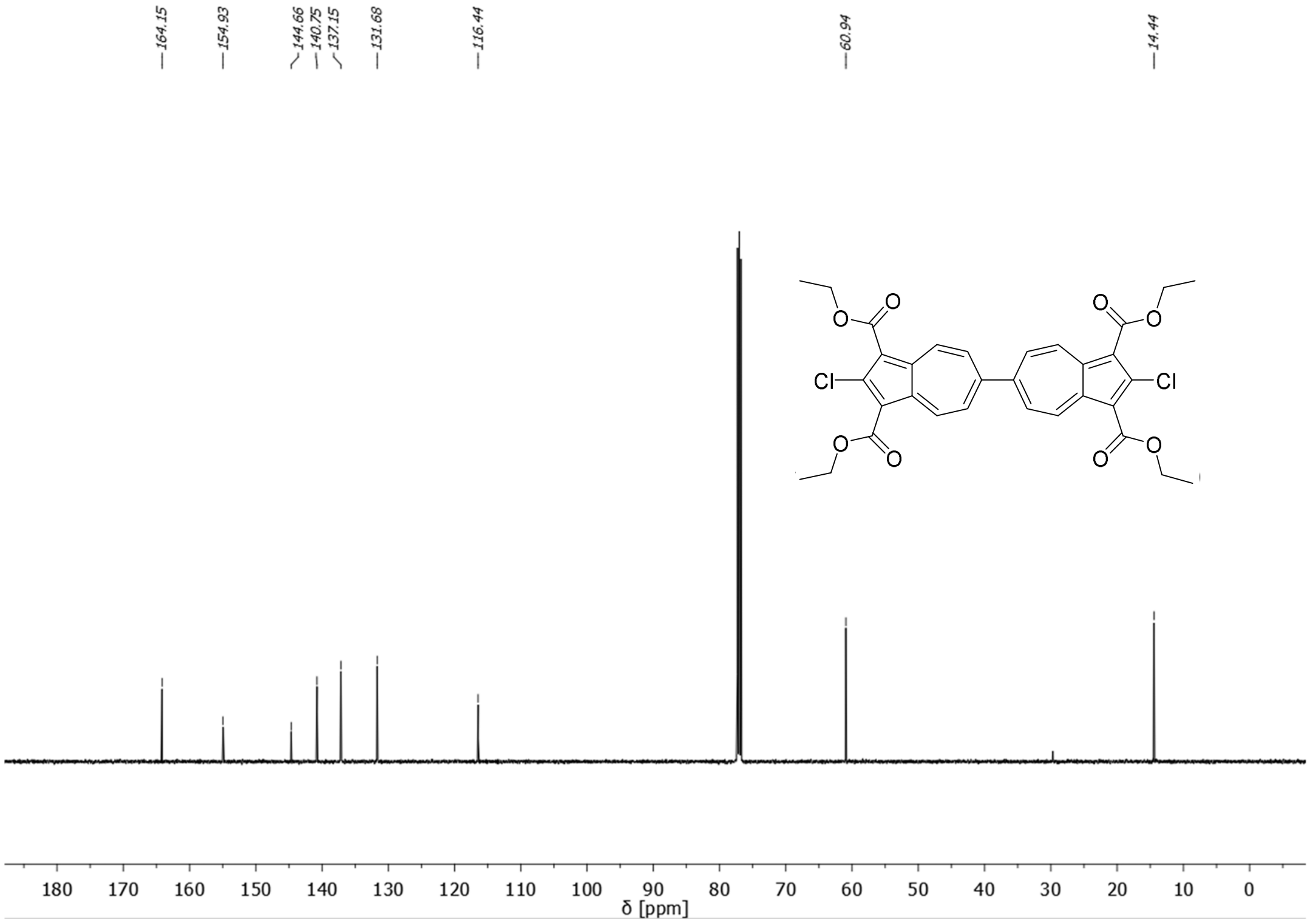

Figure S10. ^{13}C NMR spectrum (126 MHz, $CDCl_3$, 25 °C) of **3a**.

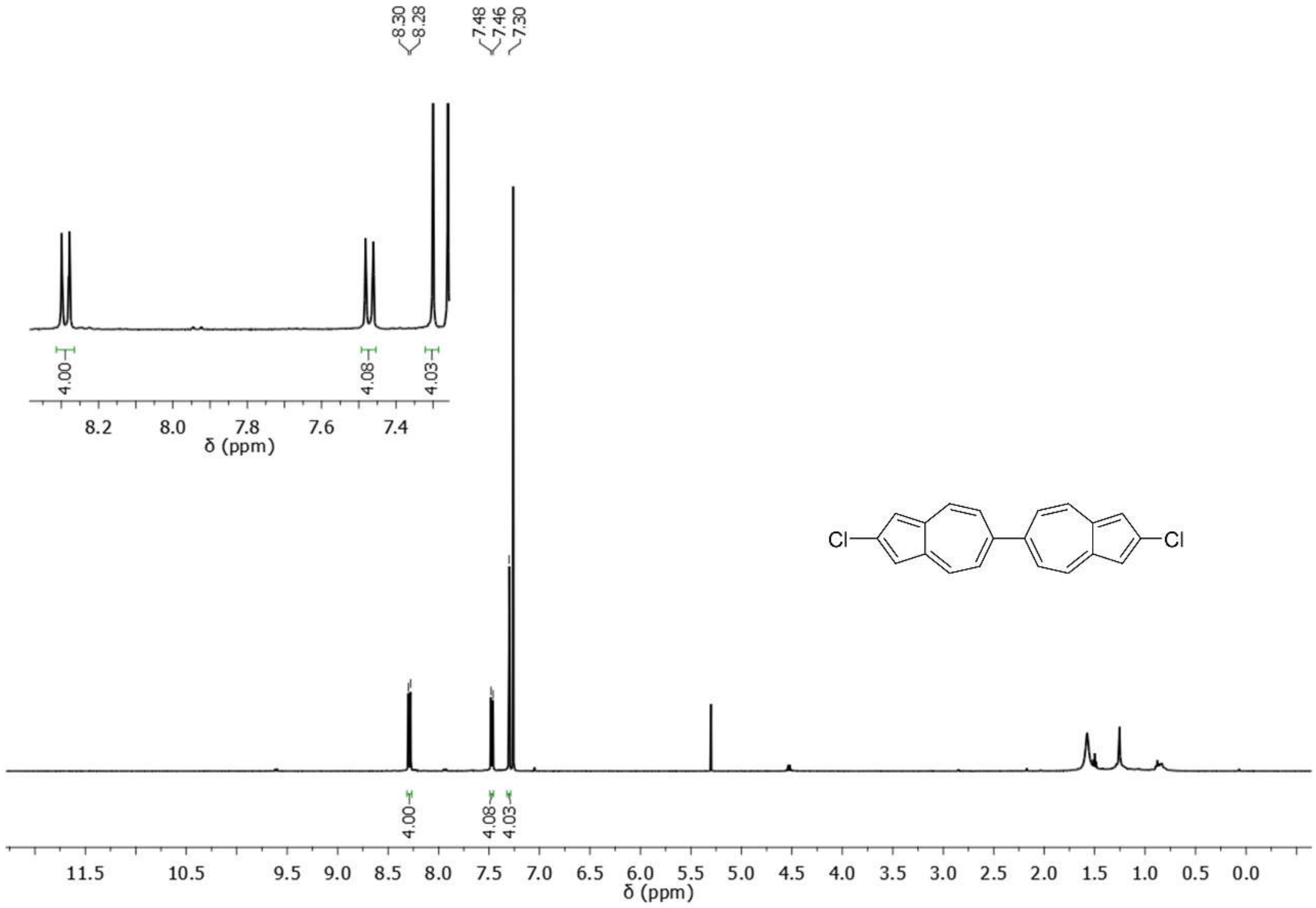

Figure S11. ^{1}H NMR spectrum (500 MHz, $CDCl_3$, 25 °C) of **3b**.

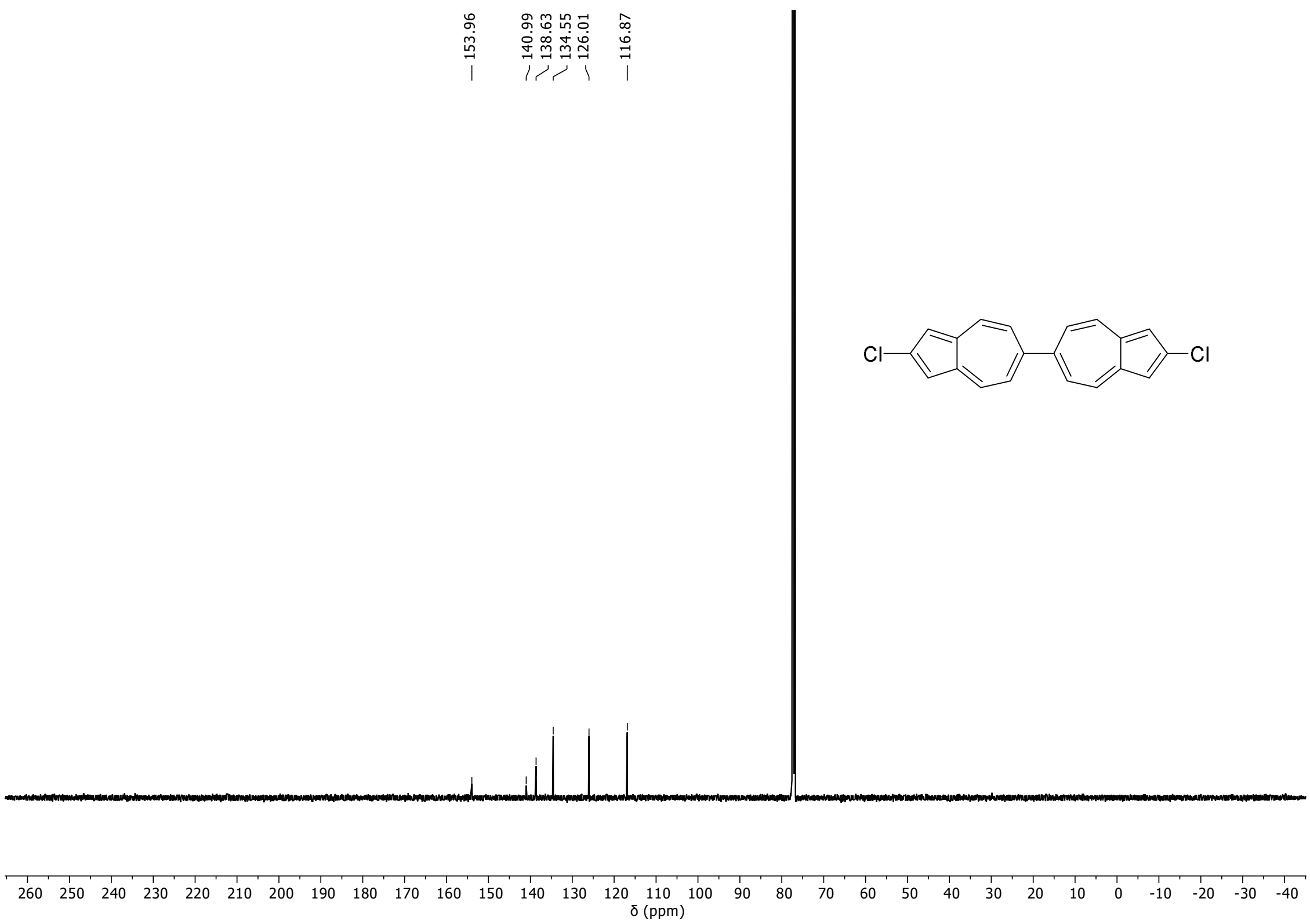

Figure S12. ^{13}C NMR spectrum (126 MHz, $CDCl_3$, 25 °C) of **3b**.

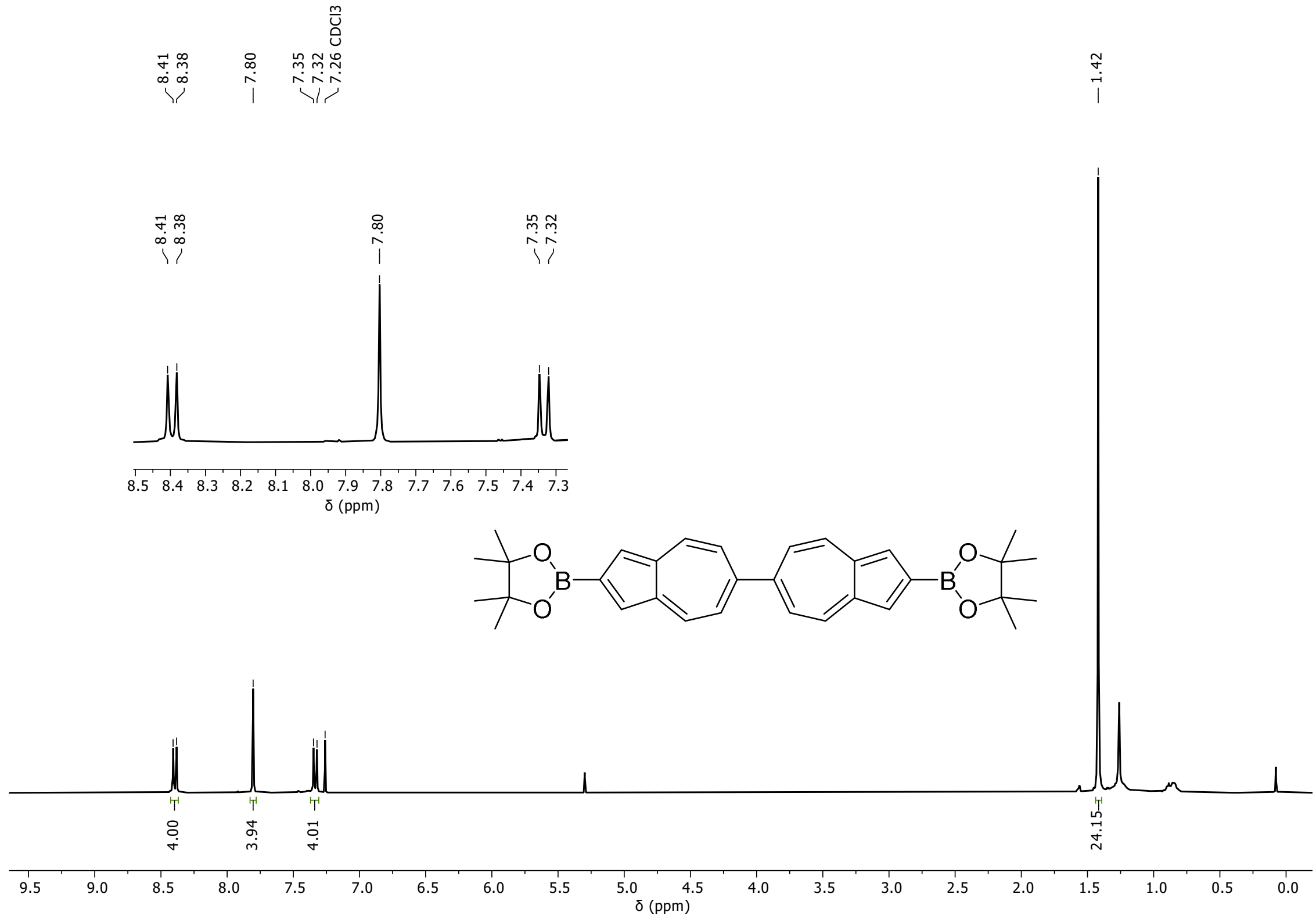

Figure S13. ^{1}H NMR spectrum (400 MHz, $CDCl_3$, 25 °C) of **4**.

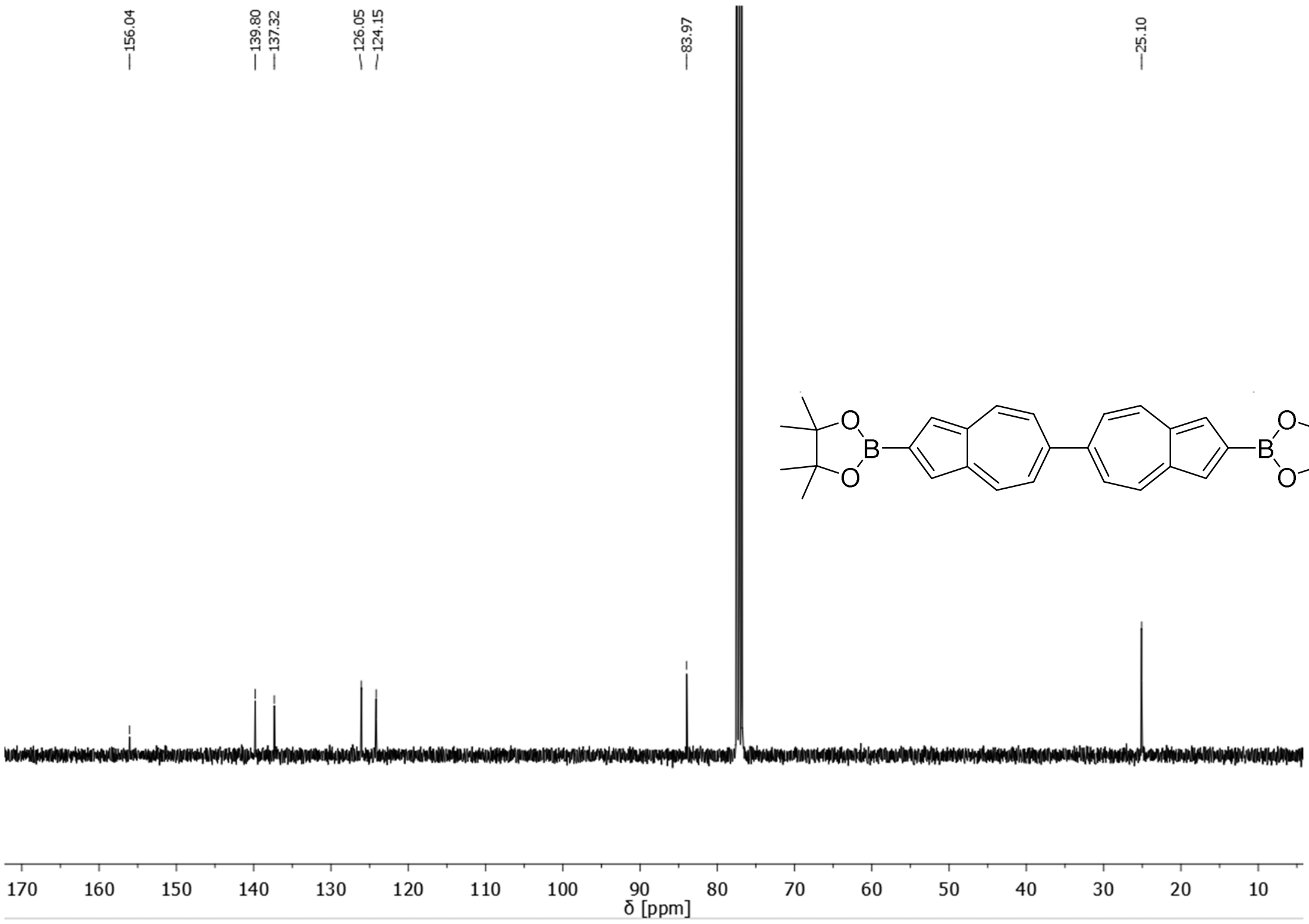

Figure S14. ^{13}C NMR spectrum (126 MHz, $CDCl_3$, 25 °C) of **4**.

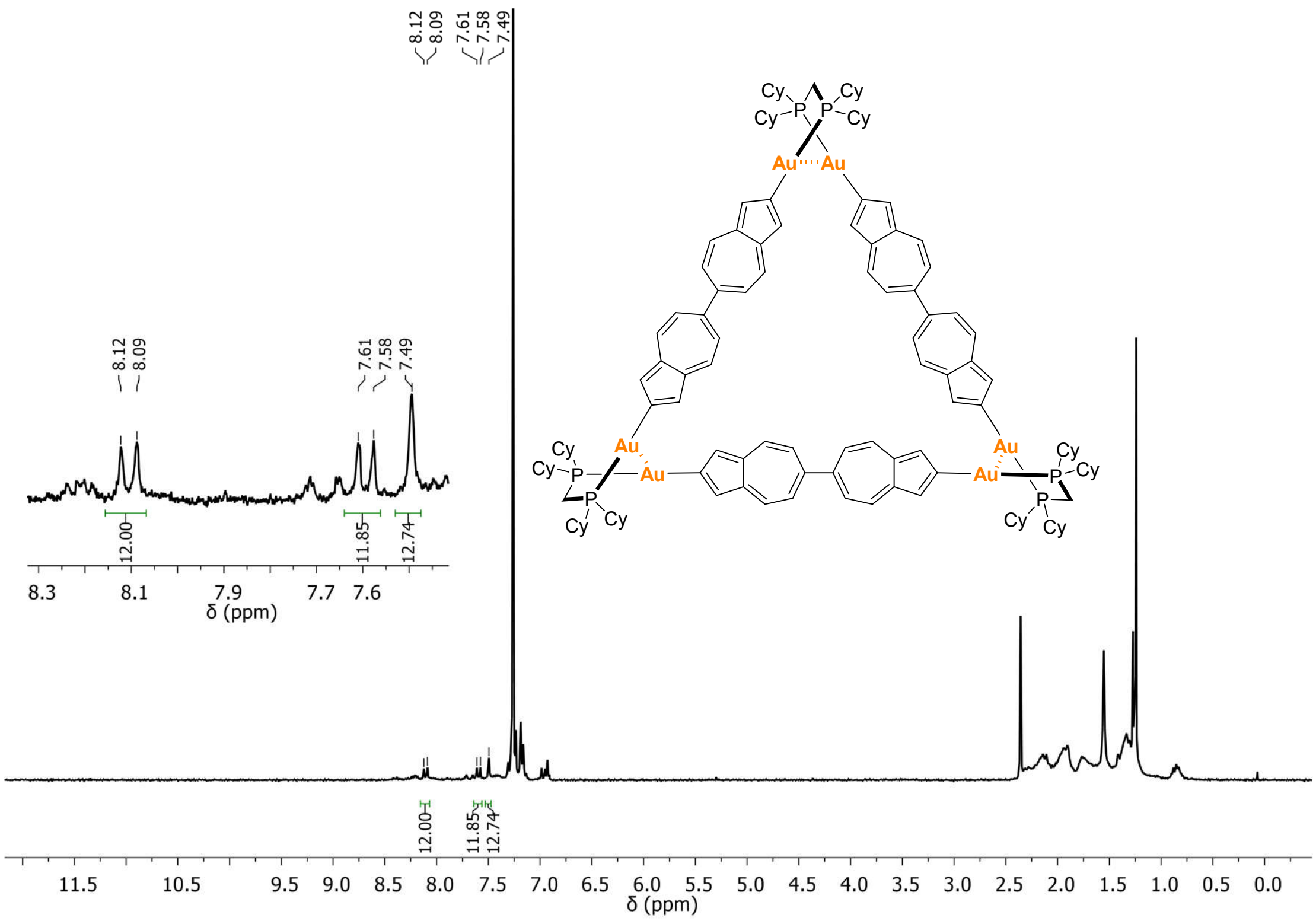

Figure S15. ^{1}H NMR spectrum (500 MHz, $CDCl_3$, 25 °C) of **5**.

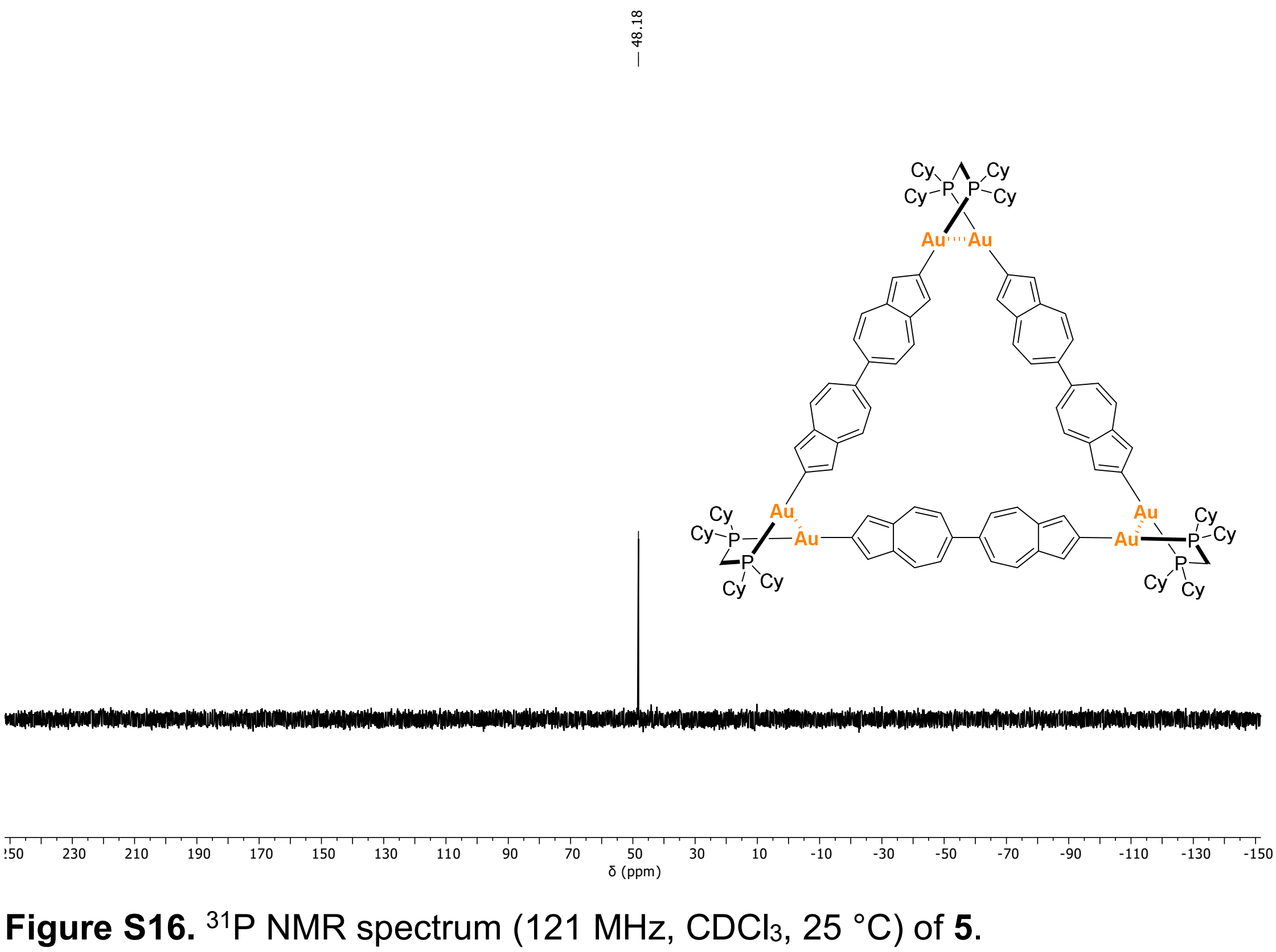

Figure S16. ^{31}P NMR spectrum (121 MHz, $CDCl_3$, 25 °C) of **5**.

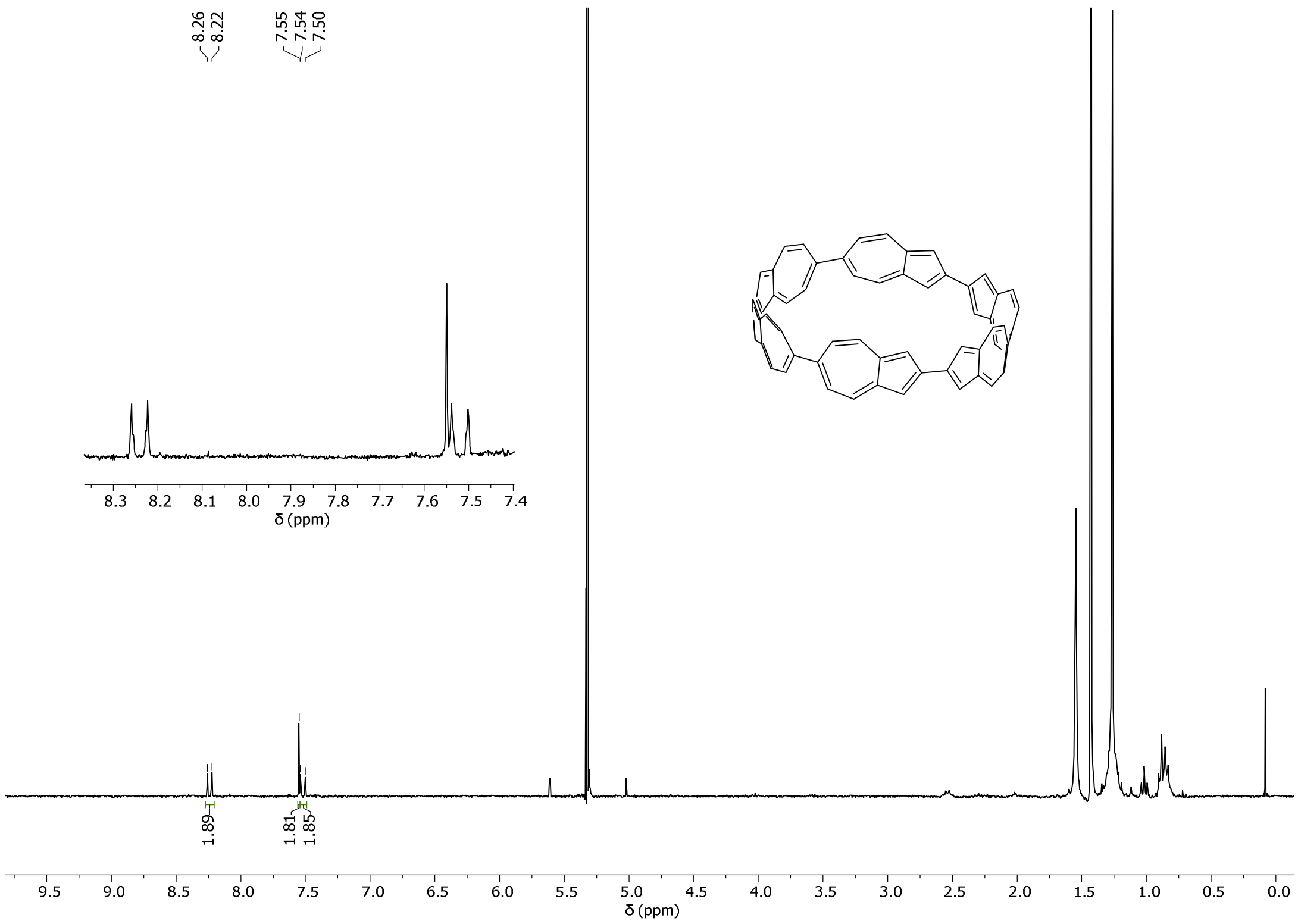

Figure S17. ^{1}H NMR spectrum (300 MHz, CD_2Cl_2, 25 °C) of **1**. The region is 0.75–1.7 ppm shows remaining grease and water signals.

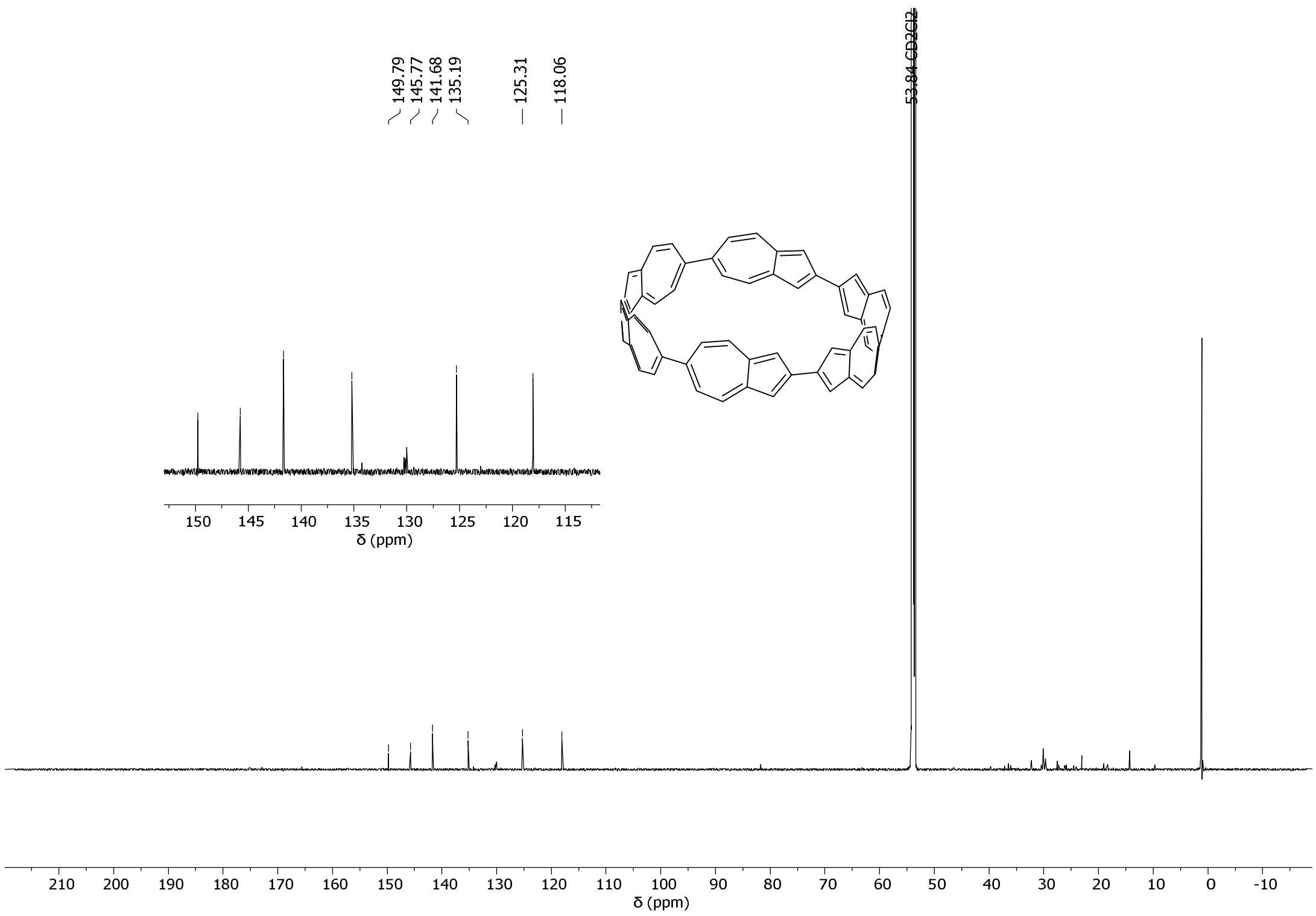

Figure S18. ^{13}C NMR spectrum (201 MHz, CD_2Cl_2, 25 °C) of **1**.

S4 Purification and Degradation Studies of [6]CPA

S4.1 Condition Screening Reductive Elimination

Table S1. Condition screening for the reductive elimination of Au macrocycle **5** to achieve **1**. Equivalents of $PhICl_2$ were calculated based on a 100% purity of crude **5**.

Entry	Solvent	$PhICl_2$	Conditions	Result
1	DMF	3 equiv.	–65 °C→25 °C	**1** and 6,6‘-biazulene (1.18:1)
2	$CHCl_3$	3 equiv.	–65 °C→25 °C	black precipitate
3	$C_2H_4Cl_2$	3 equiv.	–65 °C→25 °C	black precipitate
4	CH_2Cl_2	3 equiv.	–65 °C→25 °C	**1** and unidentified
5	CH_2Cl_2/ DMF	3 equiv	–65 °C→25 °C	**1** and 6,6‘-biazulene
6	DMF	3 equiv., KOtBu (3 equiv.)	–65 °C→25 °C	6,6‘-biazulene
7	DMF	3 equiv.	25 °C	6,6‘-biazulene and unidentified
8	DMF	3 equiv.	0 °C→25 °C	6,6‘-biazulene and unidentified
9	DMF	Old $PhICl_2$	–65 °C→25 °C	6,6‘-biazulene
10	DMF	0.3 equiv.	–65 °C→25 °C	no reaction

S4.2 Experimental UV-Vis spectra of [6]CPA

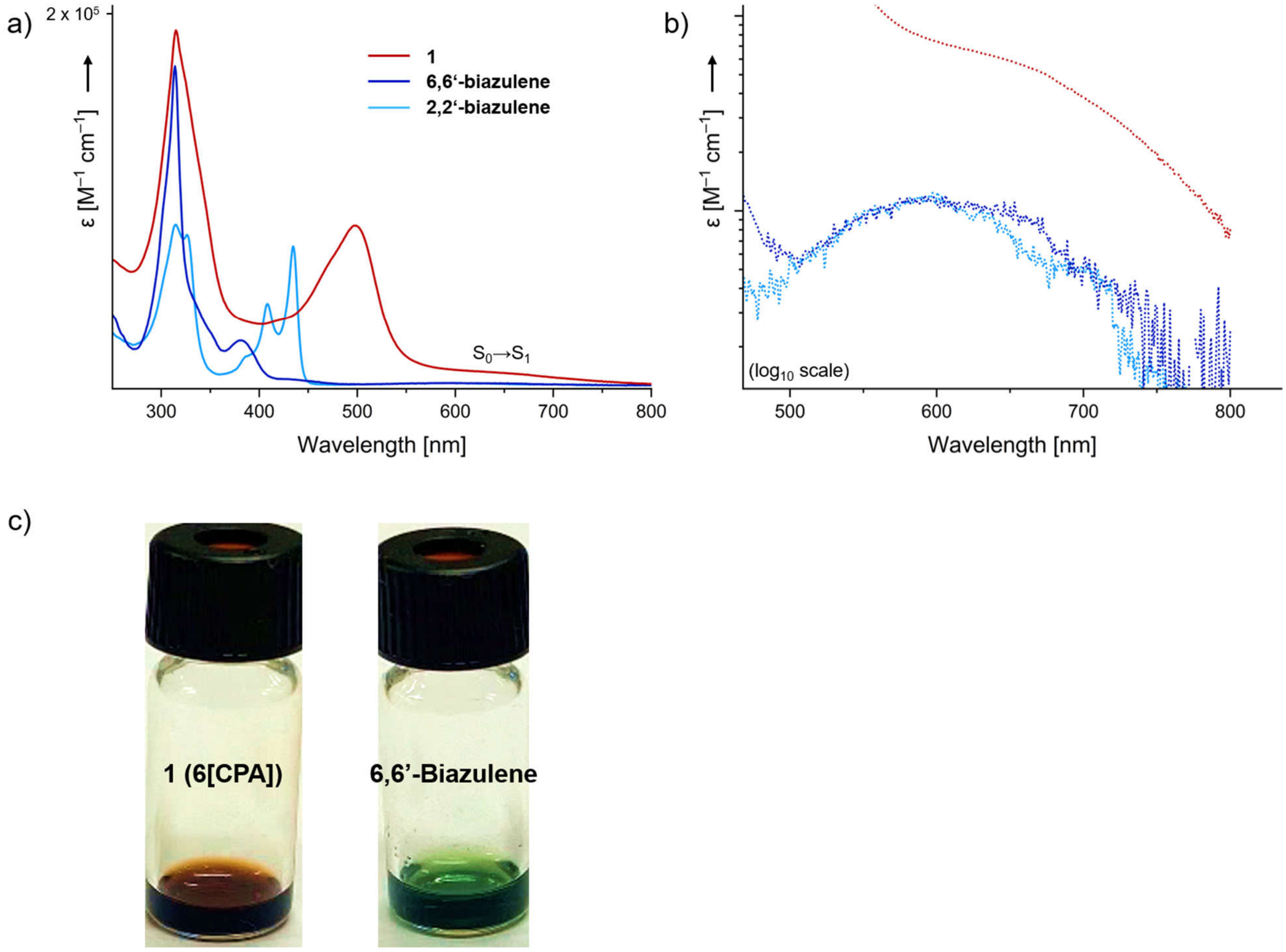

Figure S19. a) UV-Vis absorption spectra of **1**, 6,6'-biazulene, and 2,2'-biazulene in chloroform (10^{-5} M) and b) zoom of the $S_0 \rightarrow S_1$ transition region on a $\log_{10}$ scale for the absorbance coefficient; c) color comparison of dichloromethane solutions of **1** and 6,6'-biazulene.

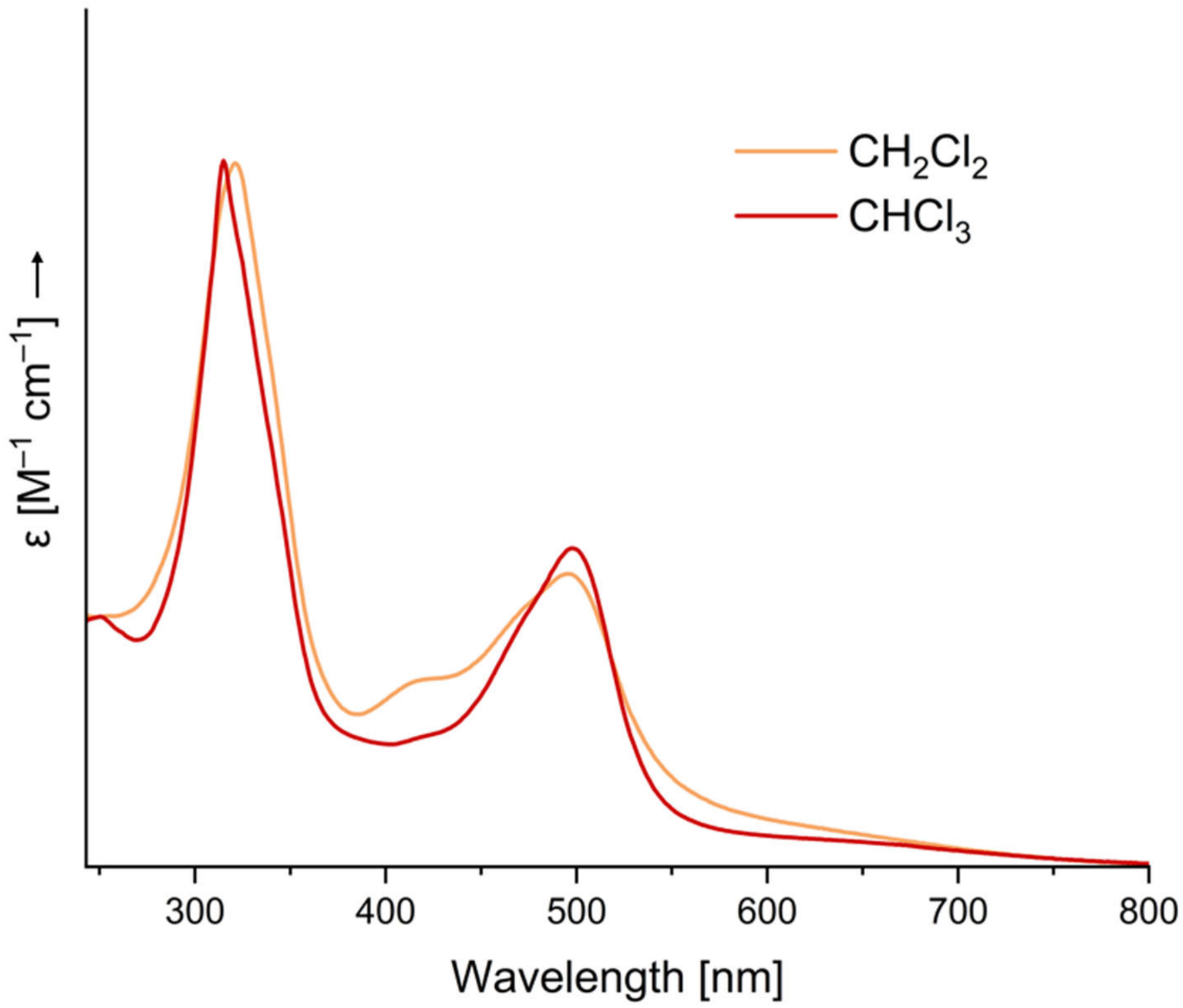

Figure S20. UV-Vis absorption spectra of **1** in dichloromethane (orange) and chloroform (red) (10^{-5} M).

S4.3 Degradation experiments on [6]CPA

Degradation during purification

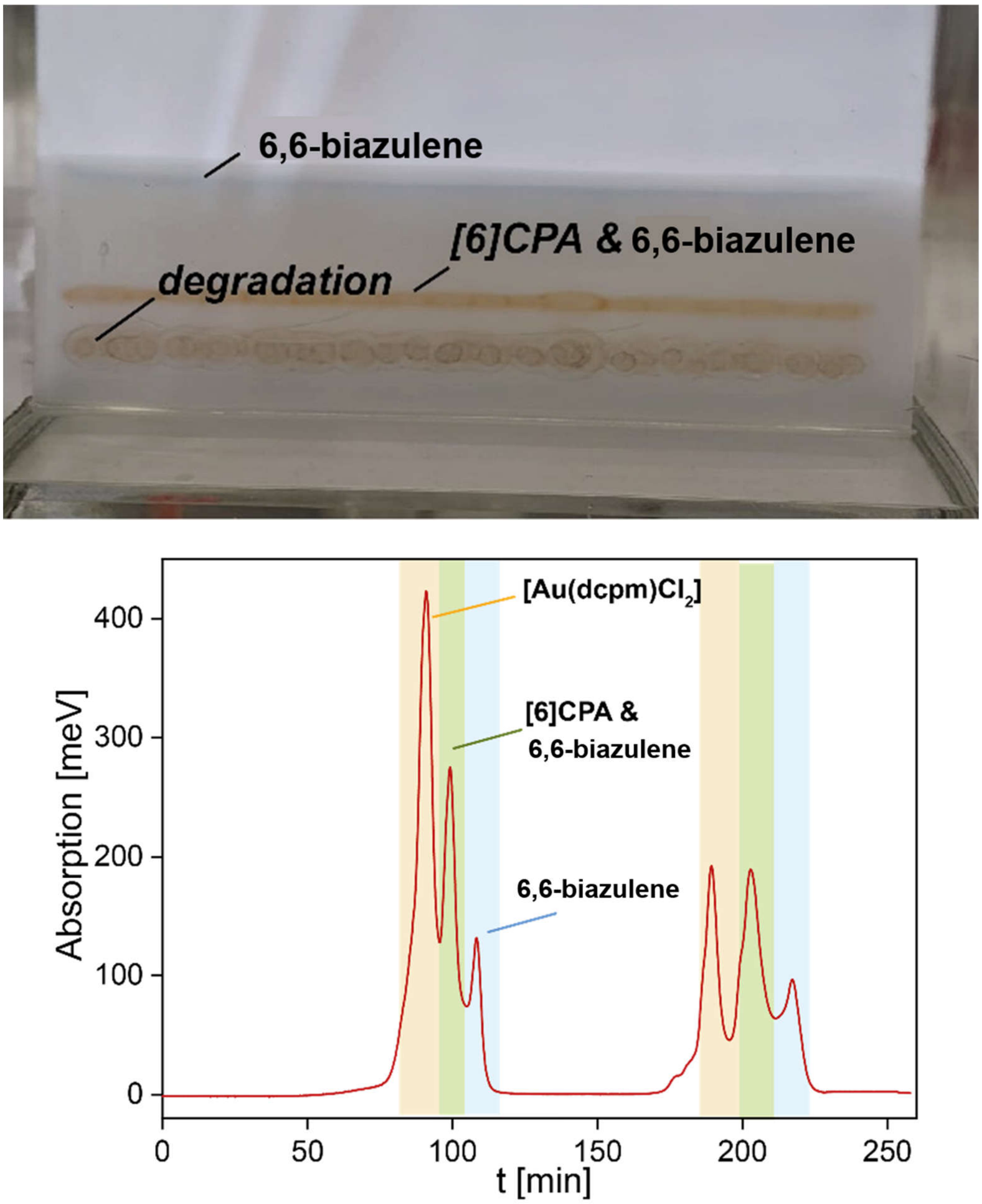

Figure S21. Photograph taken during pTLC showing degradation product on baseline, red band corresponding to a mixture of **1** and 6,6-biazulene and blue band corresponding to 6,6'-biazulene (top). *r*GPC ($CHCl_3$) chromatogram of crude reaction mixture containing [$Au_2(dcpm)Cl_2$], **1** and 6,6'biazulene (bottom).

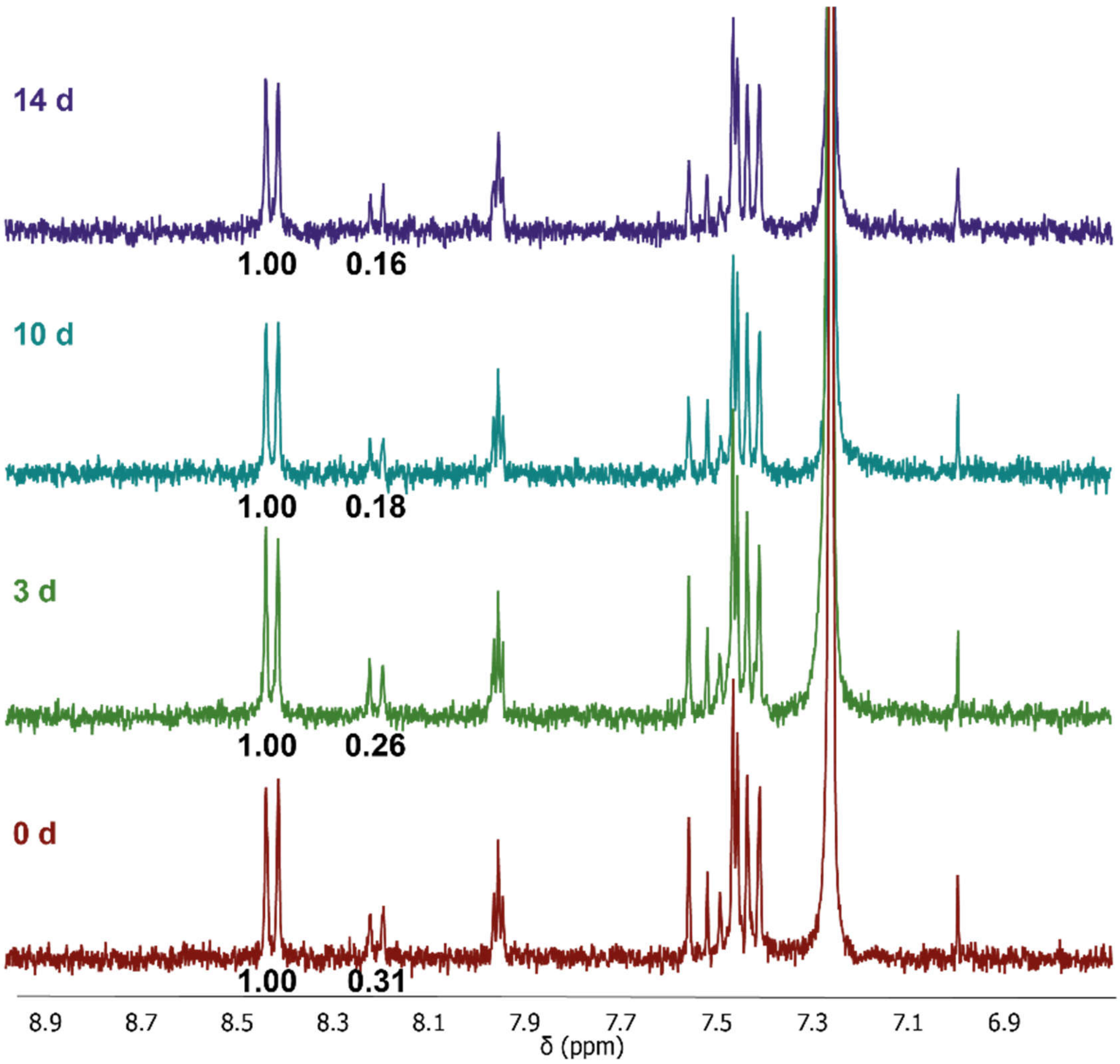

Figure S22. ^{1}H NMR spectroscopic (400 MHz, 25 °C) degradation study of mixed sample of **1** and 6,6'-biazulene in $CDCl_3$ under ambient conditions. The integral of one signal of 6,6'-biazulene was used as a reference and set to 1.00. Integrals are denoted below the signals.

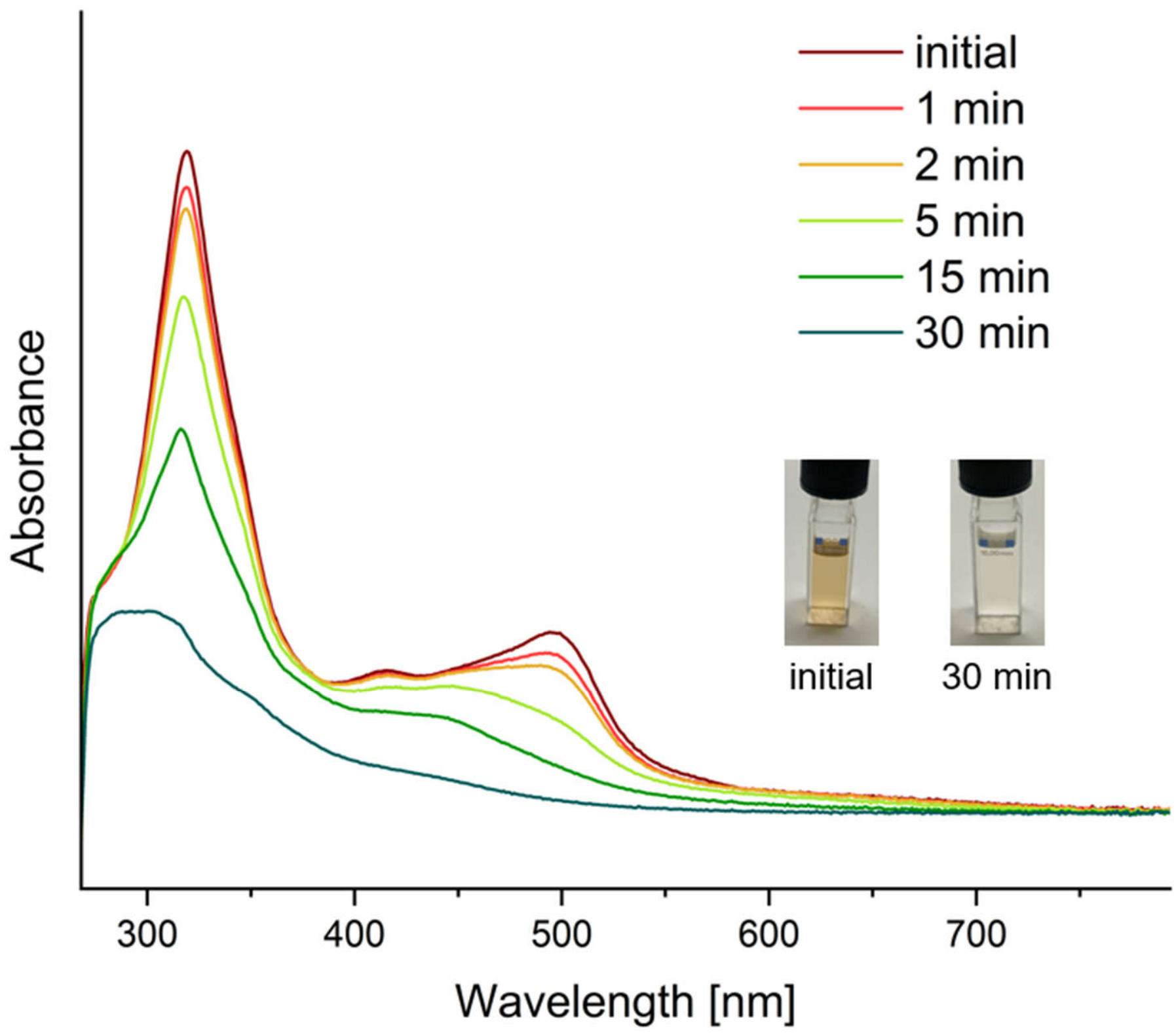

Figure S23. UV-Vis absorption spectra of **1** in Argon-purged dichloromethane (10^{-5} M) after irradiation with a 350 nm UV lamp for a given amount of time.

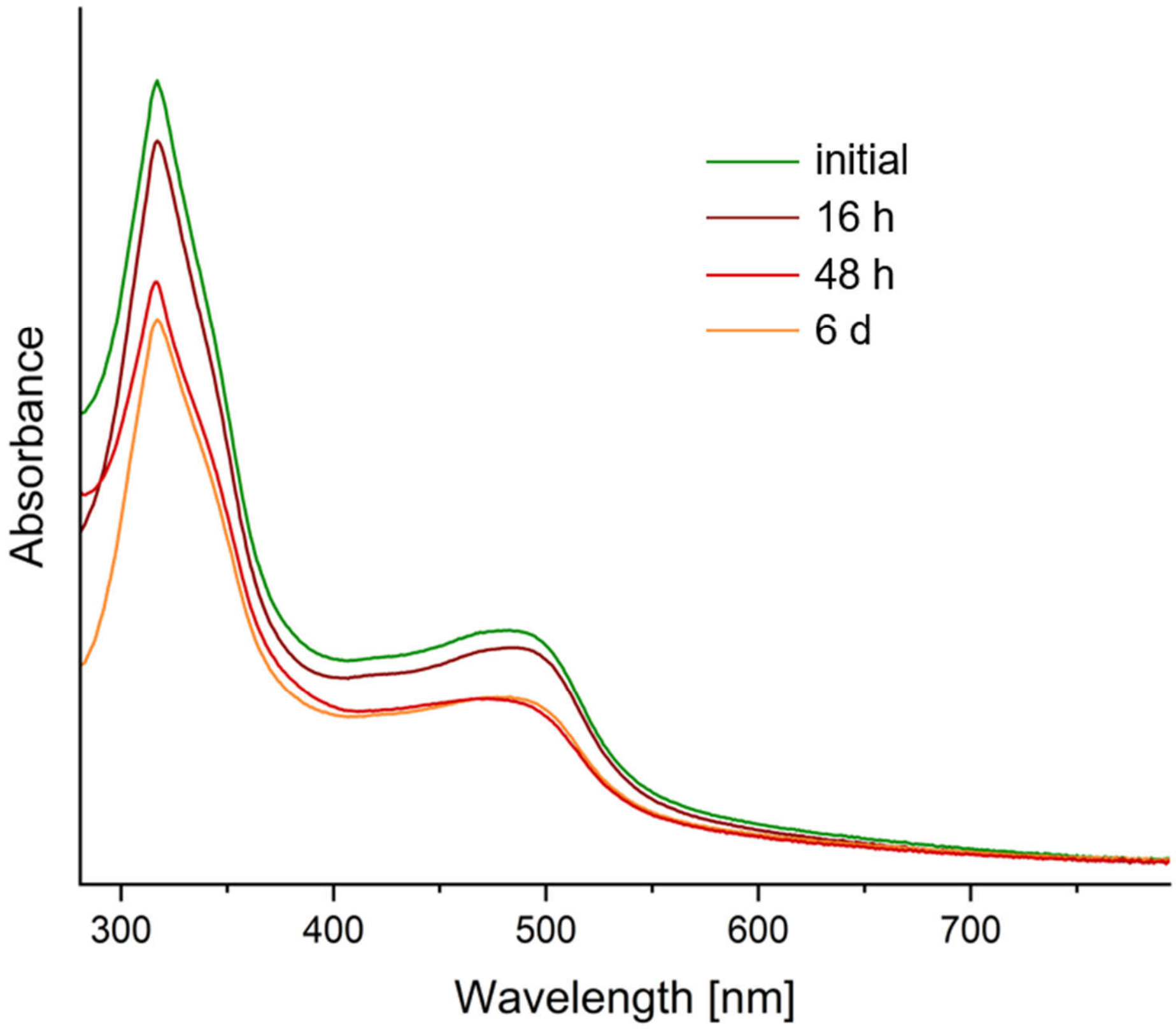

Figure S24. UV-Vis absorption spectra of **1** in dichloromethane (10^{-5} M) after exposure to air for a given amount of time. The sample was stored under exclusion of light.

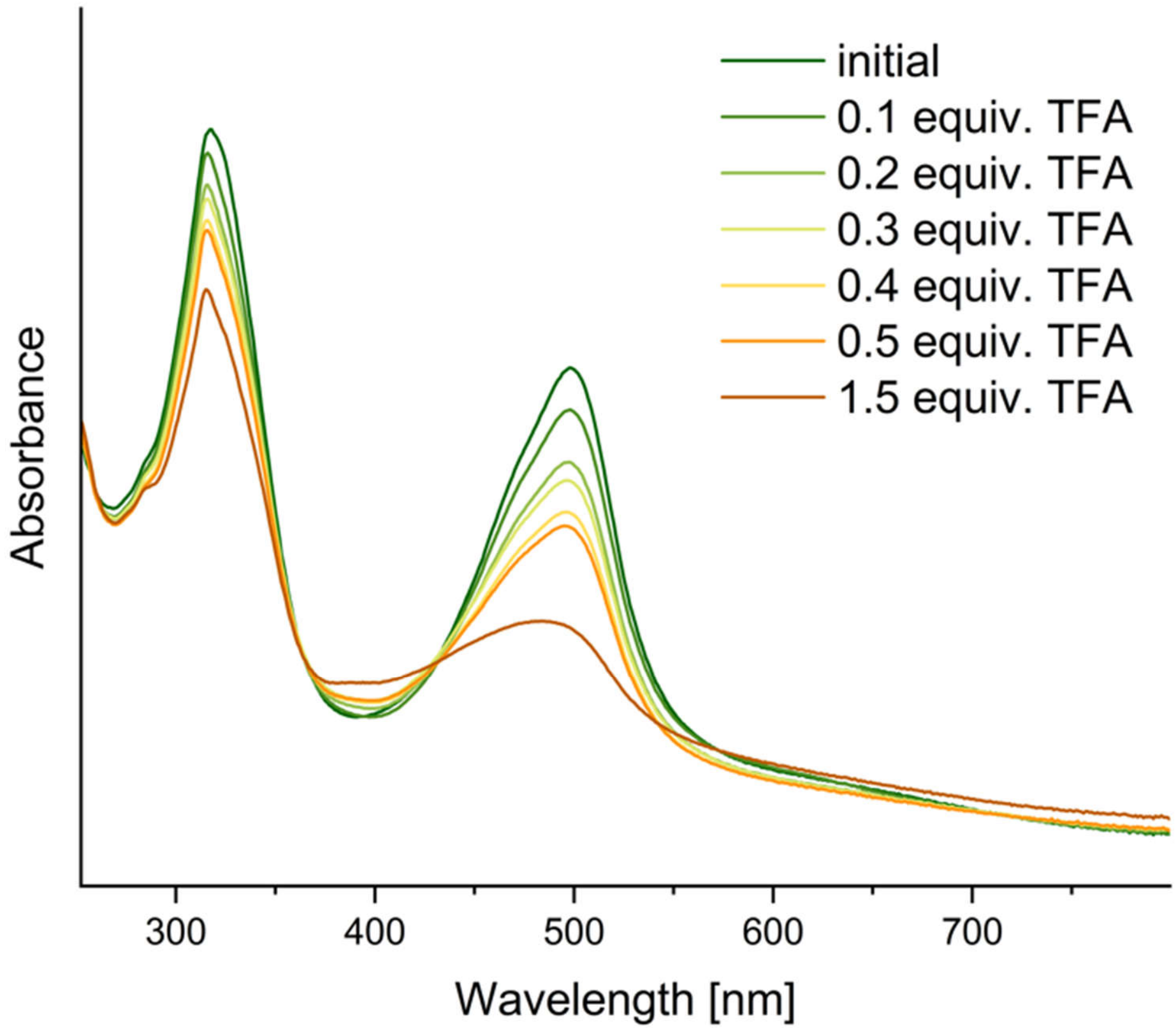

Figure S25. UV-Vis absorption spectra of **1** in chloroform (10^{-5} M) after successive addition of trifluoroacetic acid (TFA).

S5 Spectroelectrochemistry

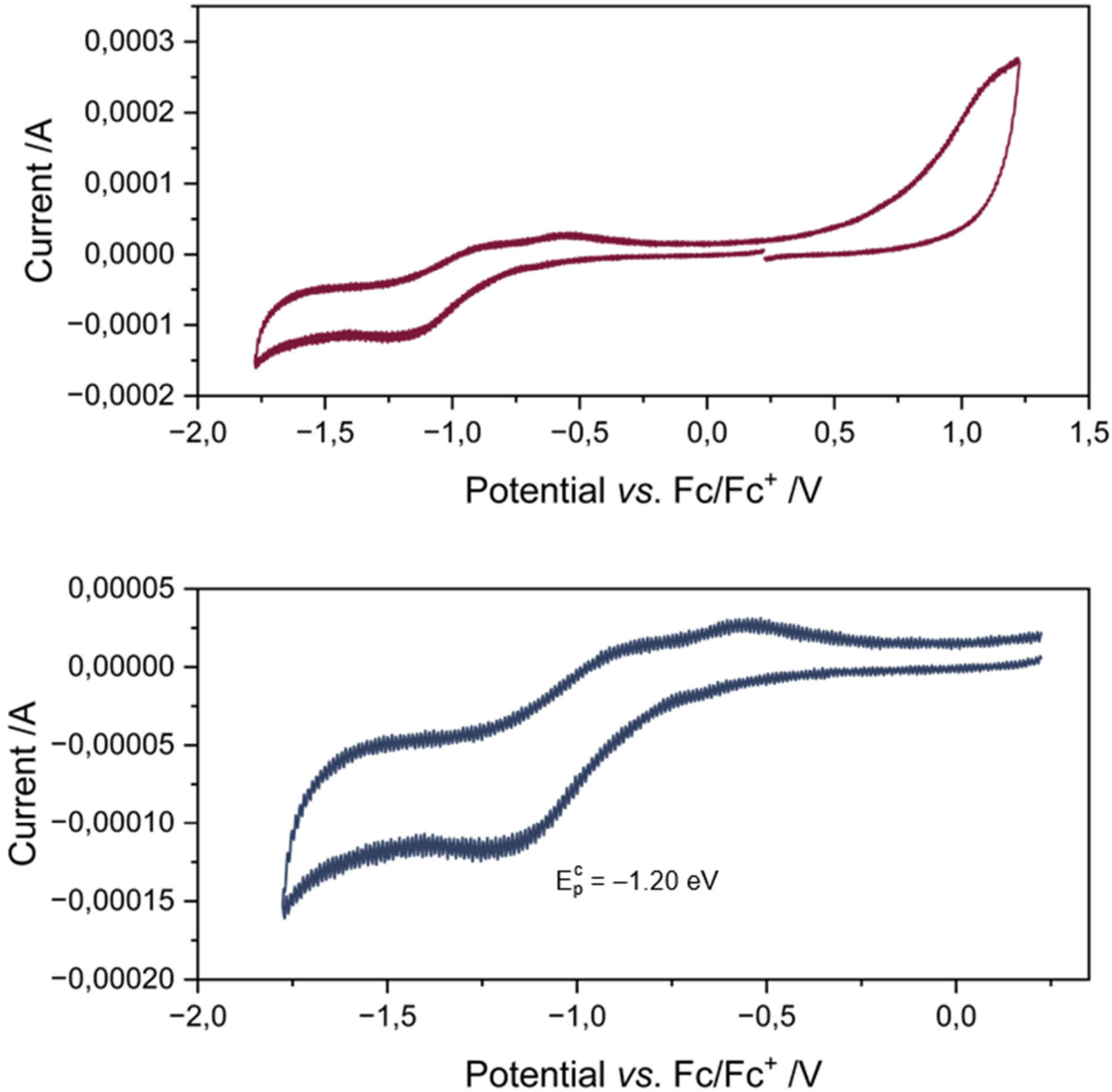

Figure S26. Cyclic voltammograms of 1 over one cycle at a scan rate of 0.1 V·s^{-1} (c = 0.5 mM with 0.1 M *n*BuN_4PF_6 in DMF).

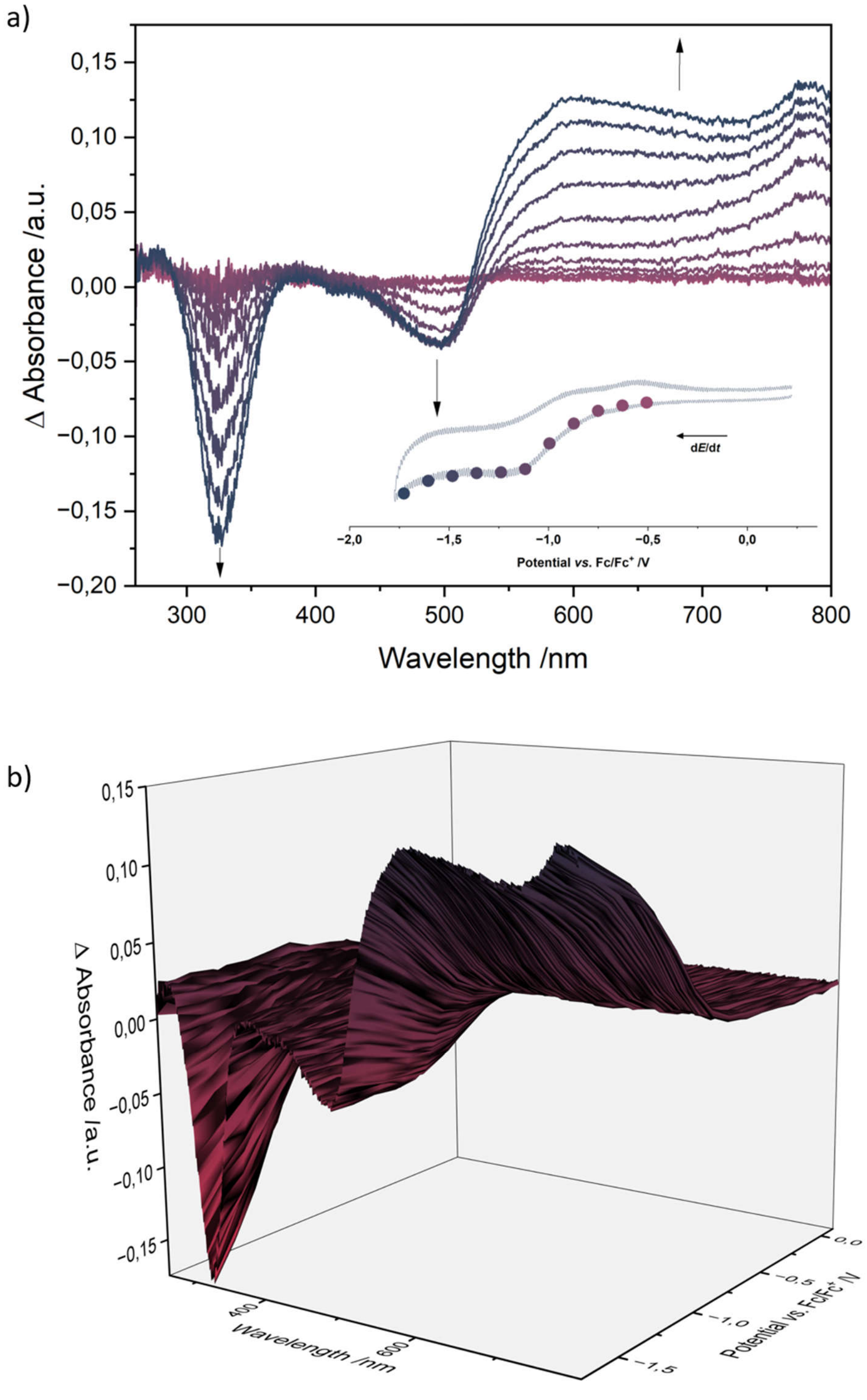

Figure S27. a) Spectroelectrochemical reduction of **1**. b) Spectroelectrochemical reduction plotted against the applied voltage. The experiment was carried out in a thin layer quartz glass spectroelectrochemical cell (*d* = 1 mm) in DMF with 0.1 M $n\text{Bu}_4\text{NPF}_6$, platinum mesh electrode, *c* = 0.5 mM, d*E*/d*t* = 10 mV s^{-1}, T = 295 K.

S6 NMR Titration Studies

Titrations were performed at 298 K to determine the association constant (K_a) of the complexation between **1** and fullerene (C_{60}).

solution of host (**1**) (~2 mM) in CS_2 was prepared first. This solution was then used as parent solution to prepare a second solution as a mixture of host and guest (C_{60}). A capillary filled with Acetone-d_6 was added to the sample as an NMR standard.

A solution of a known concentrations of guest C_{60} (excess) with **1** was added successively to the parent solution of **1**. The association constants were determined by non-linear curve fitting (using Origin 2024) of the titration curves according to the Benesi–Hildebrand method. The fitting curves were obtained by plotting the change of chemical shift of the host signals against the concentration of the guest.[10] The data was fitted to the following equation:[11]

$$\Delta\delta = \delta_{\Delta(\mathrm{HG})} \frac{\frac{1}{2} \cdot \left[\left([\mathrm{host}]_{Tot} \cdot [\mathrm{guest}] + \frac{1}{K_\mathrm{a}} \right) - \sqrt{\left([\mathrm{host}]_{Tot} + [\mathrm{guest}] + \frac{1}{K_\mathrm{a}} \right)^2 - (4 \cdot [\mathrm{host}]_{Tot} \cdot [\mathrm{guest}])} \right]}{[\mathrm{host}]_{Tot}}$$

, where the unknown parameters are: $\delta_{\Delta(HG)}$ as the chemical shift of the host–guest complex and K_a as the association constant. An error of 20% is estimated for these values. In the following the curve fitting for protons H_b of **1** are shown.

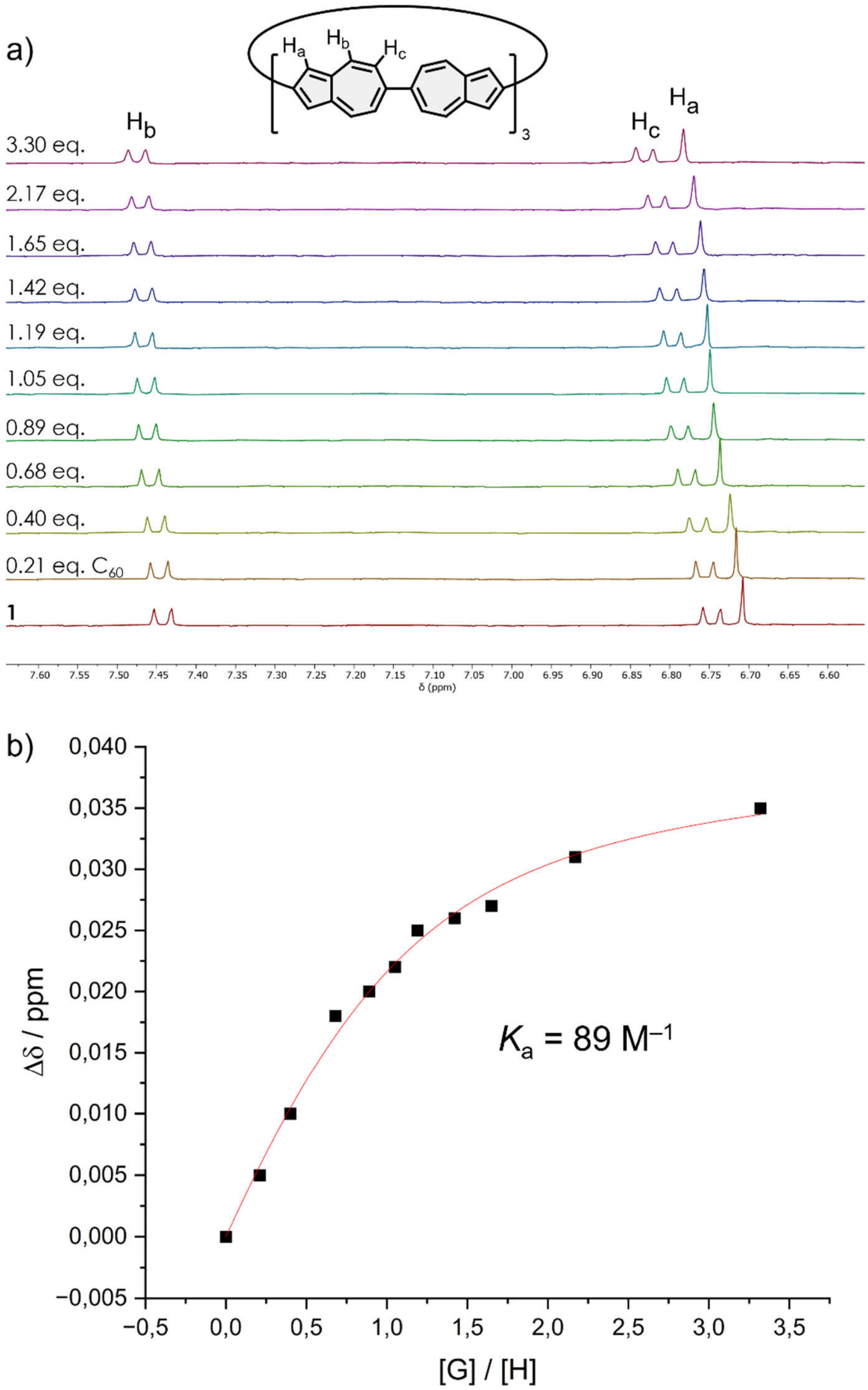

Figure S28. a) Isothermal ^{1}H NMR binding titration, spectra of **1** at 25 °C in CS_2 (c(**1**) = 2.4×10^{-3} mol L^{-1}, with Acetone-d^6 NMR standard) upon addition of C_{60} as a stock solution in CS_2. b) Non-linear least-square curve fitting analysis obtained from the isothermal binding titration upon addition of C_{60}. $\Delta\delta$ taken for proton H_b. The K_a was determined to be K_a = 89 M^{-1} with an R^2 of 0.9939.

S7 Selected mass spectra

Mass spectrum of **4**.

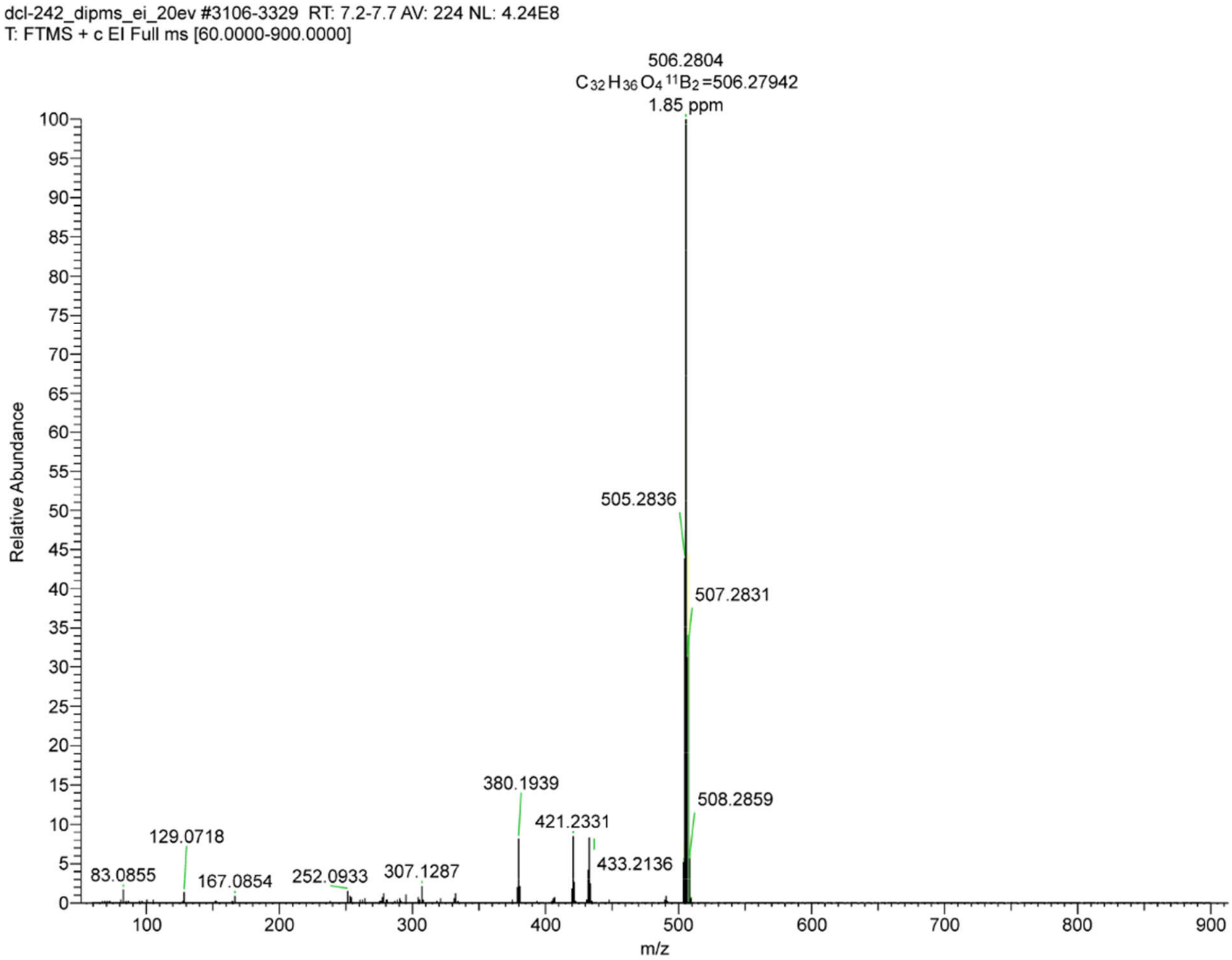

Figure S29. High-resolution mass spectrum (EI-MS) showing the mass of the molecular ion of **4**.

Mass spectra of **1**.

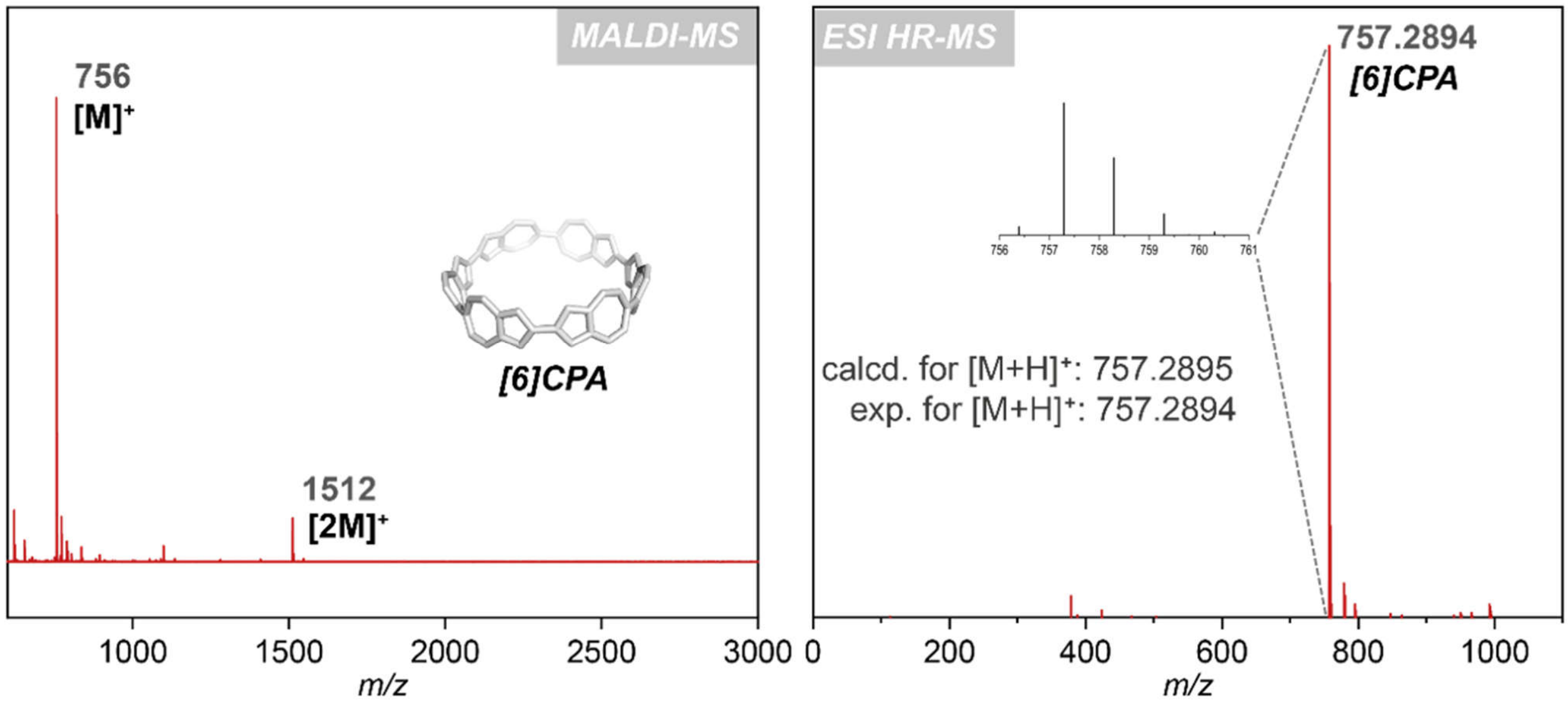

Figure S30. Mass spectra of **1** after pre-purification on *r*GPC. Left: MALDI-TOF mass spectrum showing the mass of $[M]^+$ and $[2M]^+$; Right: ESI-TOF high-resolution mass spectrum showing the mass of the protonated molecular ion.

S8 Computations

S8.1 Computational Methods

Quantum chemical calculations were performed using the ORCA 5.0.3 software package,[12–14] and, exclusively for the purpose of AICD plots and NICS maps, Gaussian 9.[15] Conformers of each compound were screened by CREST v3.0.2.[16] EDDB plots were calculated using the February 11th 2024 edition of the EDDB script,[17,18] using wavefunctions diagonalized by NBO 7.0.[19] Delocalization indices (MCI, FLU) were calculated using ESIpy.[20]

DFT (RKS) optimizations, single-point calculations (with the exception of the ESIpy calculation) and time-dependent calculations applied a def2-TZVP basis set,[21] Grimme's D3 dispersion correction[22] with Becke-Johnson damping.[23] Orca calculations also employed a RIJCOSX[24] density fitting scheme (def2/J auxiliary basis set, 'defgrid2' integration grid, 'verytighscf' and 'tightopt' constraints.

All ground state geometries and transition states were optimized by B3LYP exchange-correlation functional.[25] Geometry optimizations were carried out on pre-optimized geometries of the lowest energy conformers (found by CREST v3.0.2, applying GFN2-xTB[26] method under default constraints). All minimum energy geometries were verified by vibrational frequency calculations, applying analytical Hessian.[27] Transition states of the investigated intramolecular isomerization were calculated by the nudged elastic band (NEB) method,[28] through the climbing image approach, with a subsequent transition state optimization of the highest-energy image. Vibrational frequencies of the transition states were calculated to verify that their imaginary frequencies correspond solely to the investigated isomerization pathways.

TD-DFT calculations were carried out by B3LYP and, in a single case, CAM-B3LYP XC-functional (noted in text), applying def2-TZVP basis set,[21] Grimme's D3 dispersion correction[16] with Becke-Johnson damping.[23] Constraints and approximations were applied as previously outlined for Orca calculations. Where relevant, solvation was accounted for implicitly by the C-PCM scheme (noted in text).[29]

NICS (GIAO)[30,31] and AICD (CSGT)[32] calculations were carried out in Gaussian 9, at B3LYP/6-311+G** level of theory. ESIpy delocalization indices (MCI, FLU) were calculated using at B3LYP/ 6-311+G** level of theory and applied meta-Löwdin partitioning (sanity-checked against other partitioning approaches). Pople's basis set was chosen for these calculations specifically so that the obtained values can be compared to studies cited herein.

S8.2 Computational Results [6]CPA

S8.2.1 Geometry of [6]CPA

CREST conformer search at GFN2-xTB level of theory found two conformers, A and B, dubbed //- and /\- in text, respectively, for their contrasting geometry when viewed *in plane* (Figure S31). Optimization and subsequent analytical hessian calculation at B3LYP/def2-TZVP level of theory revealed that both conformers are minimum-energy geometries on the ground state potential energy surface, separated by ΔG = 0.96 kcal/mol. The global minimum energy geometry //- was used in the subsequent calculations, for the characterization of [6]CPA.

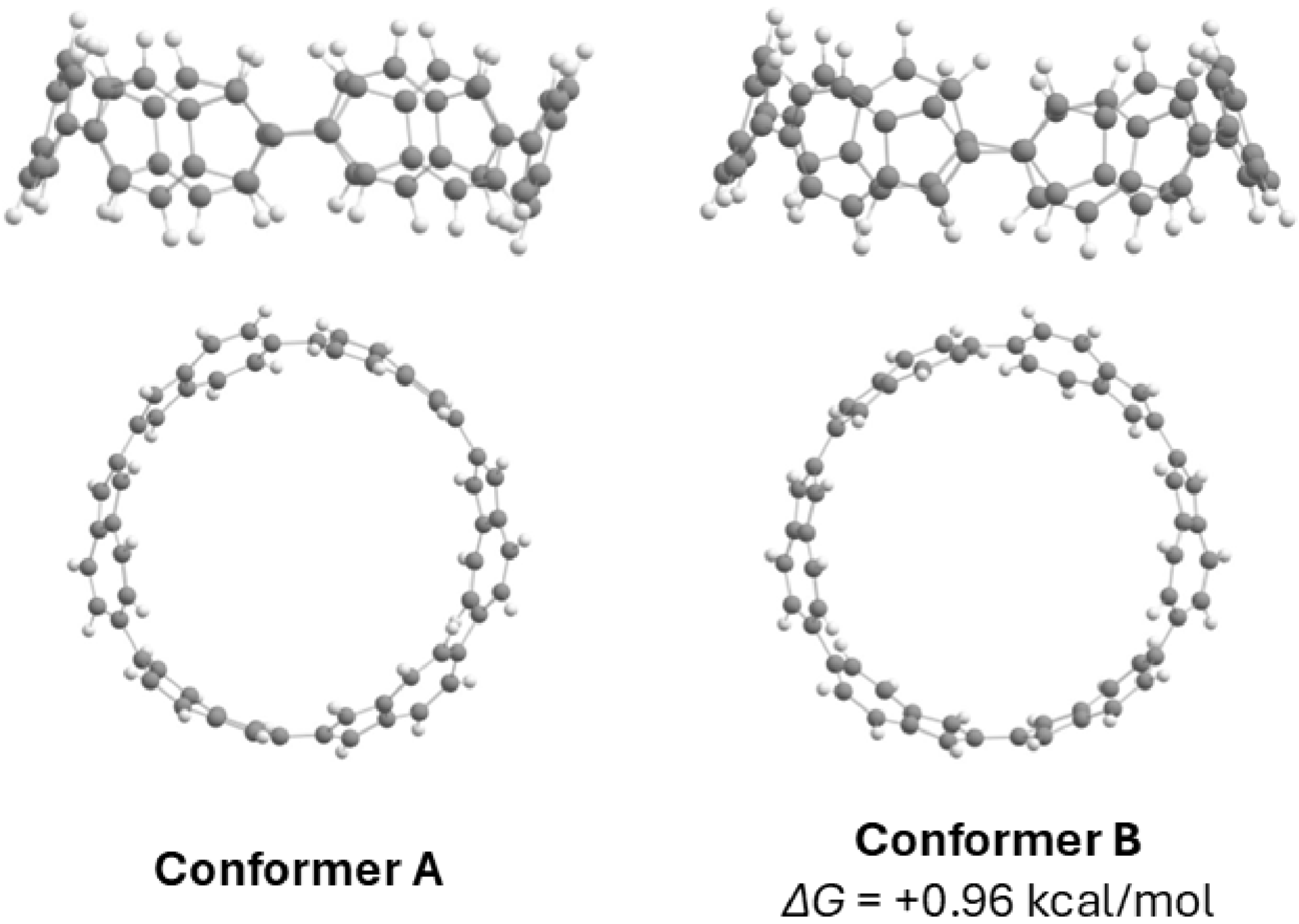

Figure S31. Geometries of optimized conformers of [6]CPA, visualized from the (top) *out of plane* and (bottom) *in plane* view, and incl. the relative Gibbs free energy of the higher energy conformer B (//), calculated at B3LYP/def2-TZVP level of theory.

Table S2. Dihedral angles between the planes of azulene subunits, measured at head-head (*hh*, C2—C2' bond) and tail-tail (*tt*, C6—C6' bond) connections of [6]CPA geometries found through CREST conformer search, at GFN2-xtb level of theory, reoptimized at B3LYP/def2-TZVP level of theory. It is revealed that the 5 identified geometries correspond to two conformers, A(//) and B(/\), which are double- and triple-degenerate, respectively.

Conformer	Geom	Dihedral Angle at Bridge Bond (°)[a]						Configuration
		*hh*1	*tt*1	*hh*2	*tt*2	*hh*3	*tt*3	
**A (//)**	1	-20.1	34.1	-20.2	33.0	-20.0	32.9	$(\text{skew}^{-1}\text{-skew})_3$
	2	20.5	-34.0	20.1	-33.0	20.1	-33.0	$(\text{skew-skew}^{-1})_3$
**B (/\)**	3	-4.7	31.0	-4.1	-31.8	26.1	-31	-(planar-skew)-(planar-skew^{-1})-(skew-skew^{-1})-
	4	-3.9	-32.2	26.0	-31.9	-3.4	30.6	-(planar-skew^{-1})-(skew-skew^{-1})-(planar-skew)-
	5	26.4	-32	-3.32	30.2	-3.6	-32.5	-(skew-skew^{-1})-(planar-skew)-(planar-skew^{-1})-

[a]Measured starting in direction of the 3-2 atom sequence, to ensure a common reference point.

Using CI-NEB, we calculated the minimum-energy pathway of the interconversion between M and P enantiomers of 1, at B3LYP/def2-TZVP level of theory. The converged pathway is symmetric, and proceeds through a D_{6h} highest-energy (climbing) image, ΔE_{CI} = 9.67 kcal/mol.

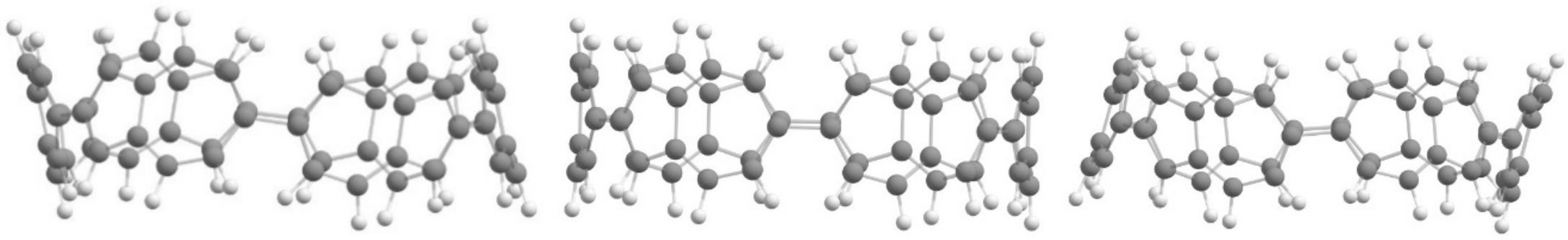

Figure S32. From the left: Starting geometry, highest-energy (climbing) image and end geometry of the calculated CI-NEB pathway, @B3LYP/def2-TZVP level of theory.

Figure S33. Energy profile of the CI-NEB pathway, corresponding to the interconversion between *M* and *P* enantiomers of 1 (ΔE_{CI} = 9.67 kcal/mol), calculated at B3LZP/def2-TZVP level of theory.

Please note that 1 has rather high number of geometric degrees of freedom (*N* atoms = 96) and is conformationally flexible. Therefore, interconversion between *M* and *P* enantiomers may proceed through a step-wise process, which cannot be accounted for by our approach.

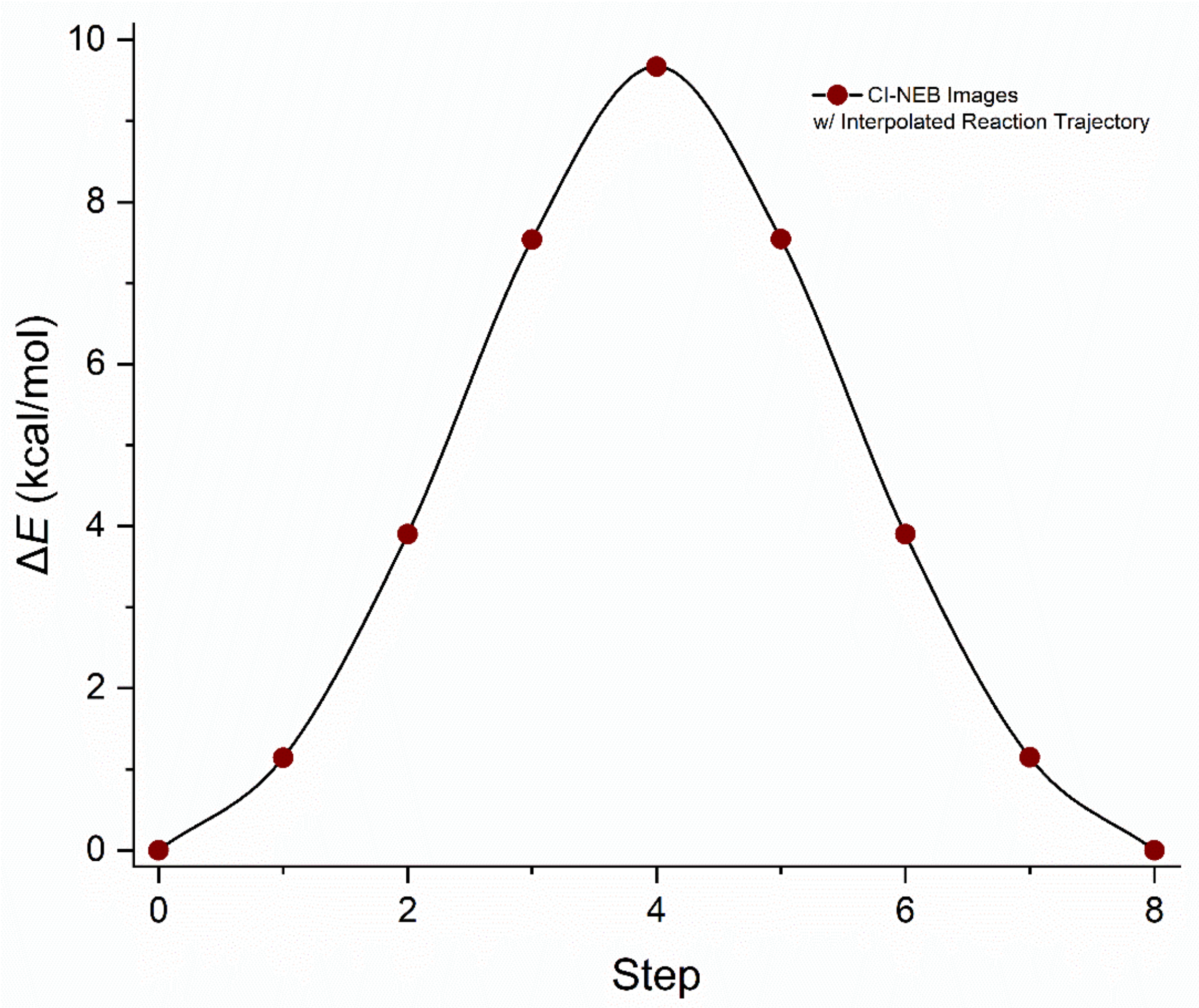

Figure S33. Energy profile of the CI-NEB pathway, corresponding to the interconversion between *M* and *P* enantiomers of 1 (ΔE_{CI} = 9.67 kcal/mol), calculated at B3LZP/def2-TZVP level of theory.

Please note that **1** has rather high number of geometric degrees of freedom (*N* atoms = 96) and is conformationally flexible. Therefore, interconversion between *M* and *P* enantiomers may proceed through a step-wise process, which cannot be accounted for by our approach.

S8.2.2 MOs and TD-DFT

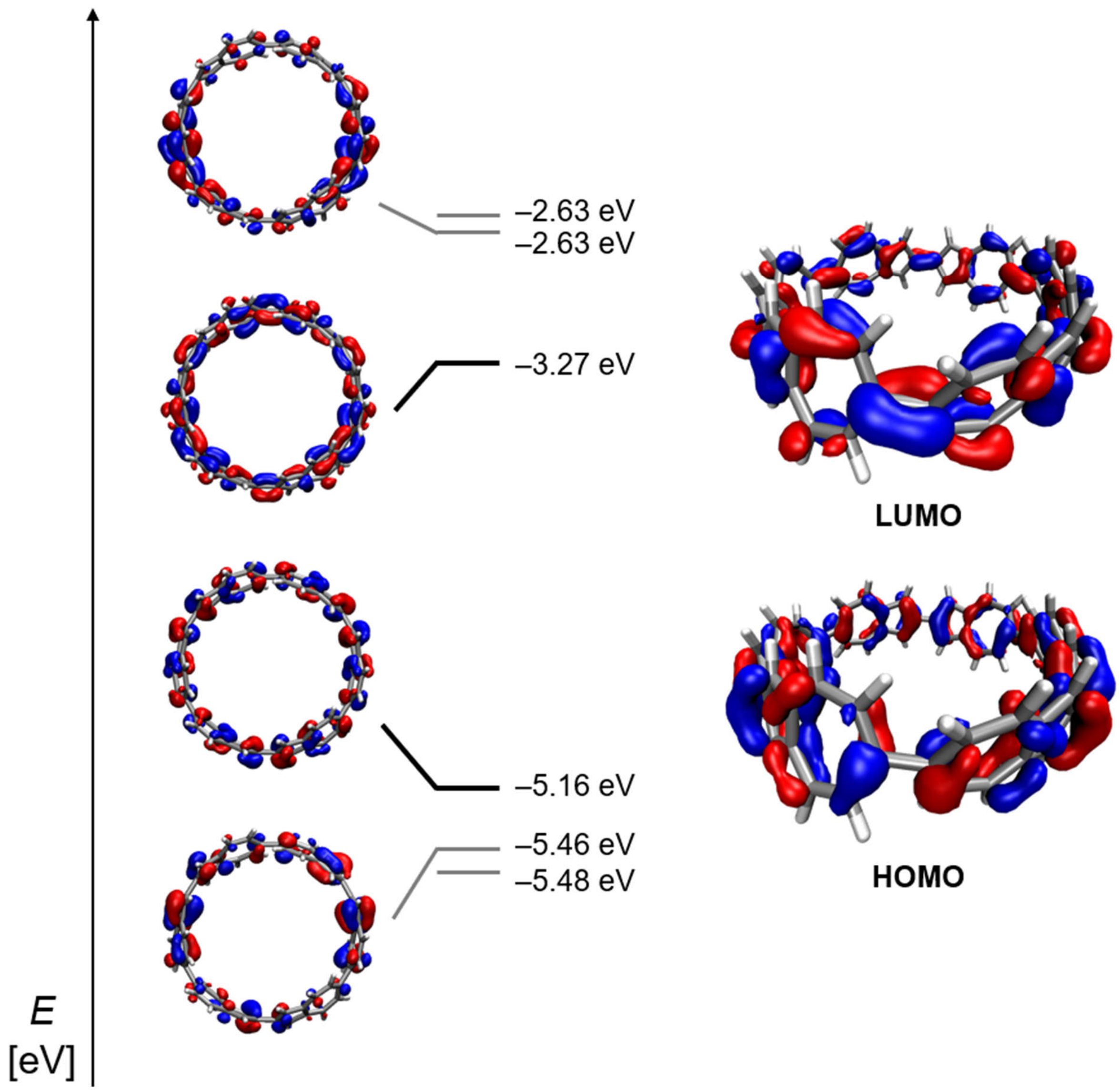

Figure S34. Frontier orbital energies (B3LYP/def2-TZVP) and orbital visualization of **1** from HOMO–2 to LUMO+2 plotted at the 0.002 $e \cdot A^{-3}$ contour, orbitals energies are given in eV.

Table S3. Calculated electronic transitions of **1** at CAM-B3LYP/def2-TZVP level of theory, applying C-PCM implicit solvent model (chloroform).

Transition	Energy (cm^{-1})	Wavelength (nm)	f_{osc}	T^2 (au^2)	T_X (au)	T_Y (au)	T_Z (au)
1	15394	650	9.68E-04	0.021	0.045	0.025	0.134
2	16675	600	8.55E-05	0.002	-0.023	0.034	0.005
3	16851	593	1.07E-02	0.210	-0.443	-0.117	0.007
4	16864	593	1.05E-02	0.204	-0.121	0.436	-0.001
5	17674	566	2.26E-04	0.004	-0.048	-0.015	-0.041
6	17700	565	2.14E-04	0.004	0.021	-0.056	-0.019
7	17940	557	6.21E-02	1.139	0.014	0.011	-1.067
8	21026	476	1.93E+00	30.274	1.356	-5.332	0.002
9	21110	474	1.97E+00	30.798	-5.378	-1.370	0.002
10	25465	393	5.27E-06	0.000	-0.004	0.007	-0.003
11	25478	393	3.31E-05	0.000	0.014	-0.015	0.002
12	27505	364	2.02E-07	0.000	-0.001	-0.001	0.000
13	28074	356	3.86E-03	0.045	0.037	-0.209	-0.004
14	28092	356	4.45E-03	0.052	0.224	0.043	0.006
15	28759	348	4.43E-03	0.051	-0.015	-0.009	0.224
16	28949	345	1.65E-02	0.188	-0.424	-0.090	0.002
17	28958	345	1.52E-02	0.172	-0.083	0.407	-0.003
18	29456	340	7.18E-05	0.001	0.016	-0.024	0.000
19	29565	338	1.77E-03	0.020	0.134	0.042	0.012
20	29599	338	3.76E-03	0.042	0.057	-0.197	-0.004
21	30118	332	7.14E-03	0.078	0.097	0.260	0.033
22	30124	332	3.24E-03	0.035	0.036	0.184	0.014
23	30135	332	1.63E-02	0.179	0.304	-0.293	0.020
24	30170	332	1.53E-02	0.166	-0.369	-0.173	0.026
25	31000	323	3.77E-03	0.040	0.082	-0.182	0.002

26	31007	323	3.75E-03	0.040	-0.181	-0.083	-0.019
27	31044	322	6.53E-04	0.007	0.029	0.006	-0.078
28	31243	320	1.14E-05	0.000	0.001	0.011	0.001
29	31729	315	2.91E-05	0.000	0.010	-0.014	0.000
30	31931	313	1.45E-03	0.015	0.120	0.021	0.007
31	31981	313	1.95E-03	0.020	0.023	-0.140	-0.004
32	32117	311	2.06E-03	0.021	-0.046	-0.025	-0.135
33	33175	301	2.57E+00	25.505	1.329	-4.872	0.006
34	33185	301	2.63E+00	26.095	4.927	1.350	-0.013
35	33522	298	1.65E-02	0.162	-0.393	-0.076	-0.044

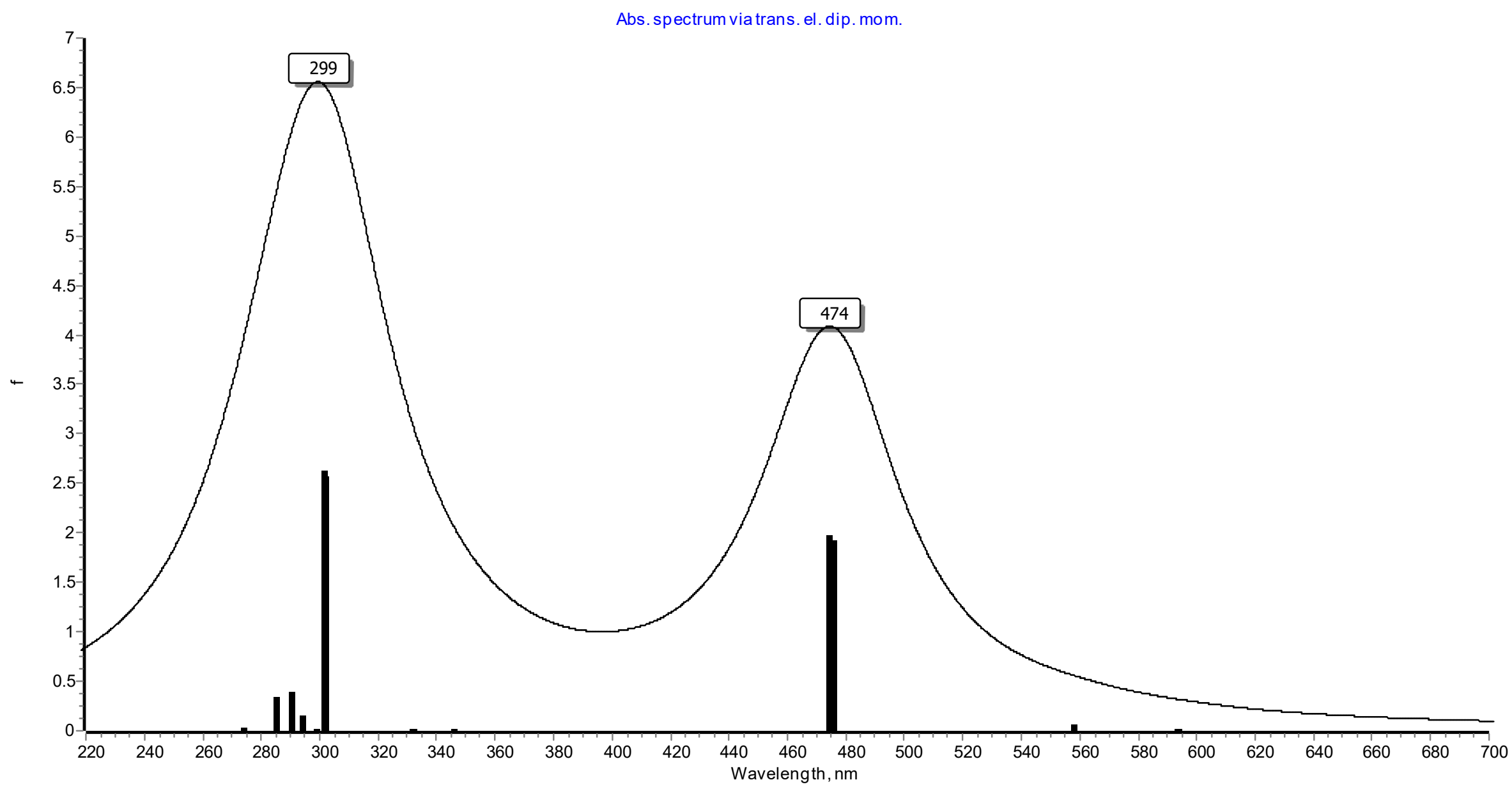

Figure S35. Plotted oscillator strengths calculated electronic transitions of **1** at CAM-B3LYP/def2-TZVP level of theory, applying C-PCM implicit solvent model (chloroform).

S8.2.3 Strain Calculations

Calculations were run at the B3LYP/6-31G(d) level of theory on structures optimized at the B3LYP/def2-TZVP level of theory. Three fragments were used to calculate the strain. Each fragment consisted of an azulene tetramer truncated at the 2,2'-positions. For a detailed description how to perform calculations using StrainViz, see the cited literature.[33]

Table S4. Calculated strain energies of **1** (conformer A and B) and [9]CPP in kcal mol^{-1} (level of theory: B3LYP/6-31G(d)).

Compound	[6]CPA Conformer A	[6]CPA Conformer B	[9]CPP
**Total Strain**	74.1	70.6	64.2
**Bond Strain**	8.4	8.0	-
**Angle Strain**	3.5	5.2	-
**Dihedral Strain**	62.3	57.4	-

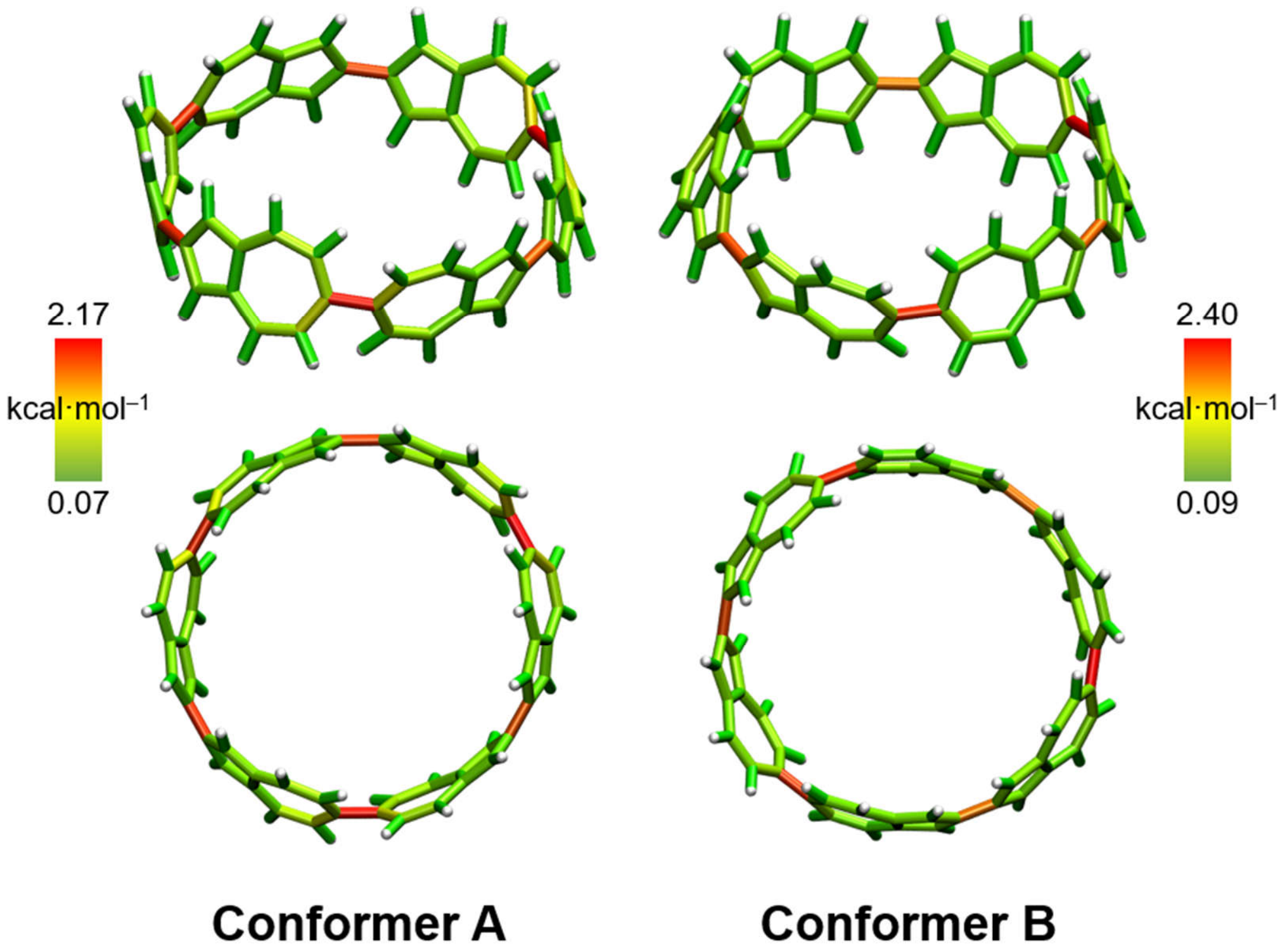

Figure S36. StrainViz representations of **1** (Conformer A and B). Based on geometry optimization at the B3LYP/def2-TZVP level of theory.

S8.2.4 Aromaticity Analysis of [6]CPA

(Anti)Aromaticity of [6]CPA in its ground state was evaluated by a combination of geometric, electronic (delocalization), magnetic and energetic indices, calculated for the 1) [6]CPA 60π-macrocycle, and 2) individual azulene subunits and their 10M, 7M and 5M rings (Figure S37). The //-[6]CPA conformer was used in these calculations because it is 1) the conformer found by XRD, and 2) C_3 symmetric, thereby narrowing down the calculations of (2) to a single biazulene subunit.

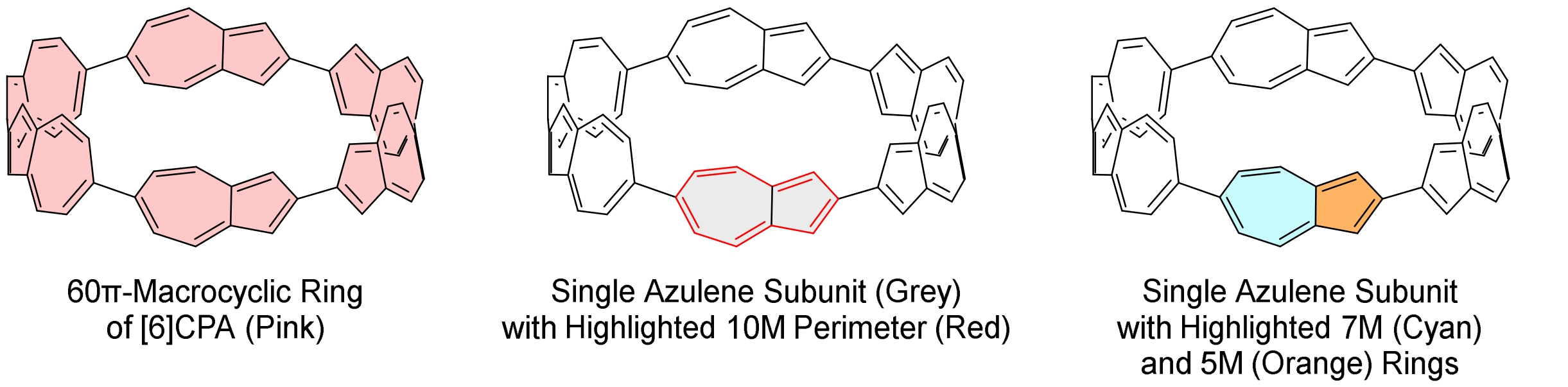

Figure S37. Highlighted delocalization circuits of [6]CPA.

S8.2.5 Geometric and Electronic Delocalization Indices (HOMA, FLU, MCI)

Table S5. Summary of aromaticity indices calculated for two neighbouring azulene subunits (*a* and *b*) of //-[6]CPA in its optimized minimum-energy geometry, within their 5-member, 7-member and 10-member circuits: harmonic oscillator model of aromaticity (HOMA), multicenter bond index (MCI) and aromatic fluctuation index (FLU), calculated at B3LYP/6-311+G** level of theory.

Subunit	Ring	HOMA	FLU	MCI
	7M	0.60	0.012	0.022
a	*5M*	0.40	0.016	0.043
	10M	**0.92**	**0.0025**	**0.024**
	7M	0.60	0.012	0.022
b	*5M*	0.40	0.016	0.043
	10M	**0.92**	**0.0028**	**0.024**

S8.2.6 Nucleus-Independent Chemical Shifts (NICS & NICS Heatmaps)

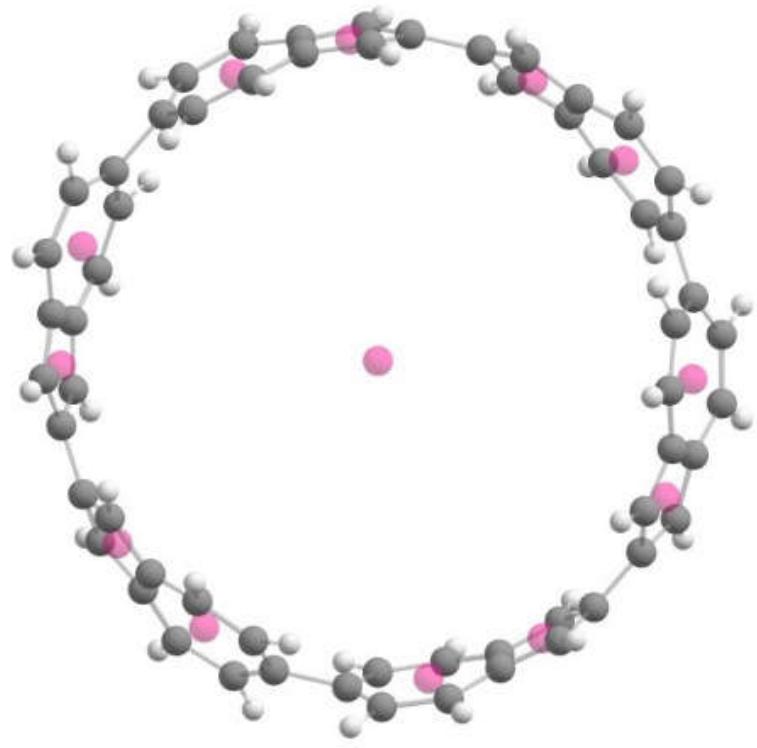

Figure S38. Dummy atom (pink) setup for $NICS_{zz}(0)$ calculations. Global centroid (at the center of //-[6]CPA) was calculated as the centroid of all 5M and 7M rings of the azulene/biazulene subunits. $NICS_{zz}(1)$ and $NICS_{zz}(1.7)$ were calculated using dummy atoms placed *above* the centroids, tangentially to the respective ring planes, in the outward direction of the macrocycle.

Table S6. Full overview of calculated $NICS_{zz}$ values in //-[6]CPA, calculated at B3LYP/6-311+G** level of theory.

$NICS_{zz}$	Centroid	5M	7M	7M	5M	5M	7M	7M	5M	5M	7M	7M	5M
0.0	**7.1**	-19.5	-3.1	-3.4	-19.7	-20.2	-1.9	-3.5	-19.1	-20.3	-3.4	-2.3	-21.5
1.0	6.8	-36.2	-13.8	-13.8	-36.6	-36.6	-12.5	-13.4	-36.0	-36.7	-13.7	-12.9	-36.9
1.7	6.3	**-23.7**	**-14.6**	**-14.5**	**-24.0**	**-24.0**	**-14.3**	**-14.4**	**-23.6**	**-24.0**	**-14.6**	**-14.3**	**-24.1**

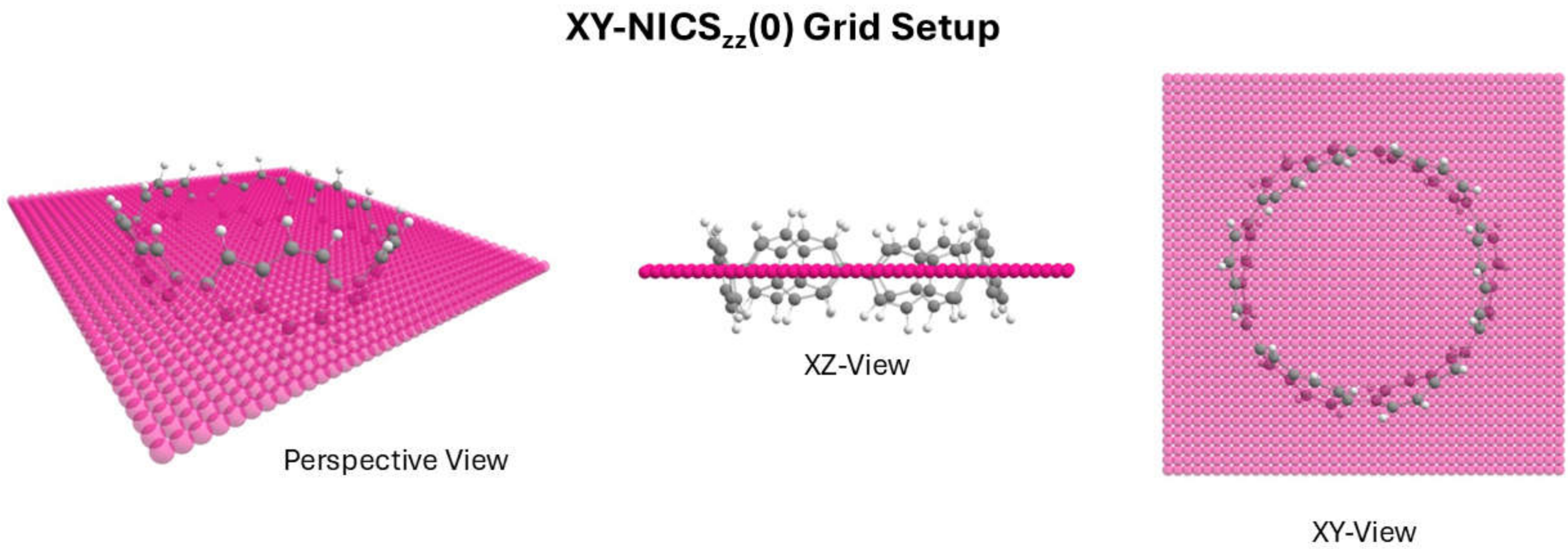

Figure S39. Dummy atom (pink) setup for $NICS_{zz}(0)$ heatmap calculations in the XY plane of [6]CPA. The grid was oriented in the xy plane which was, in turn, oriented to intersect with centroids of all azulene 5M and 7M rings.

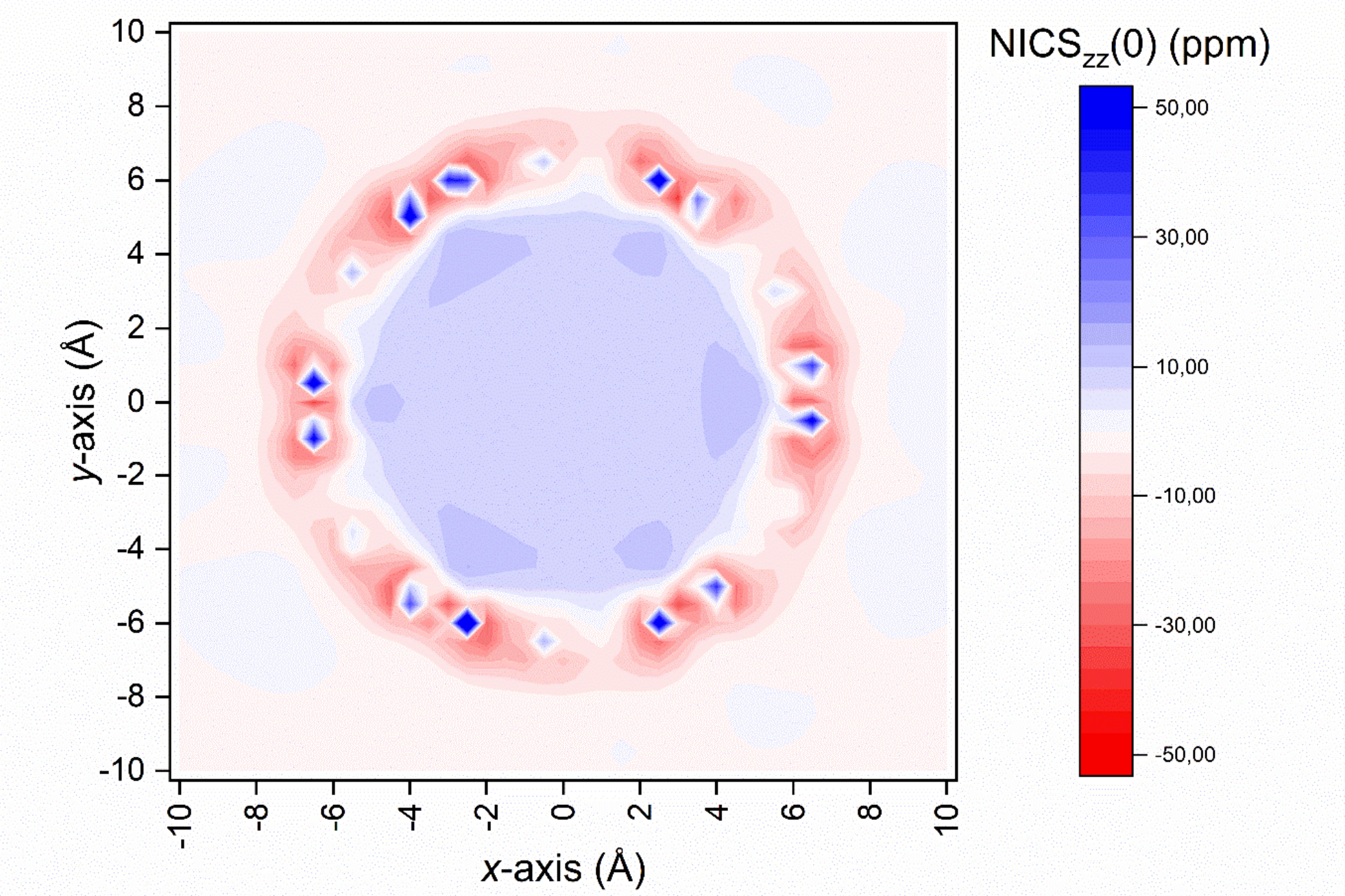

Figure S40. Calculated NICS$_{zz}$(0) heatmap in xy plane of //-[6]CPA (**neutral**), in its S_0 optimized minimum energy geometry. Calculation was performed using a 10x10 Å grid with 0.5 Å step, at B3LYP/6-311+G** level of theory.

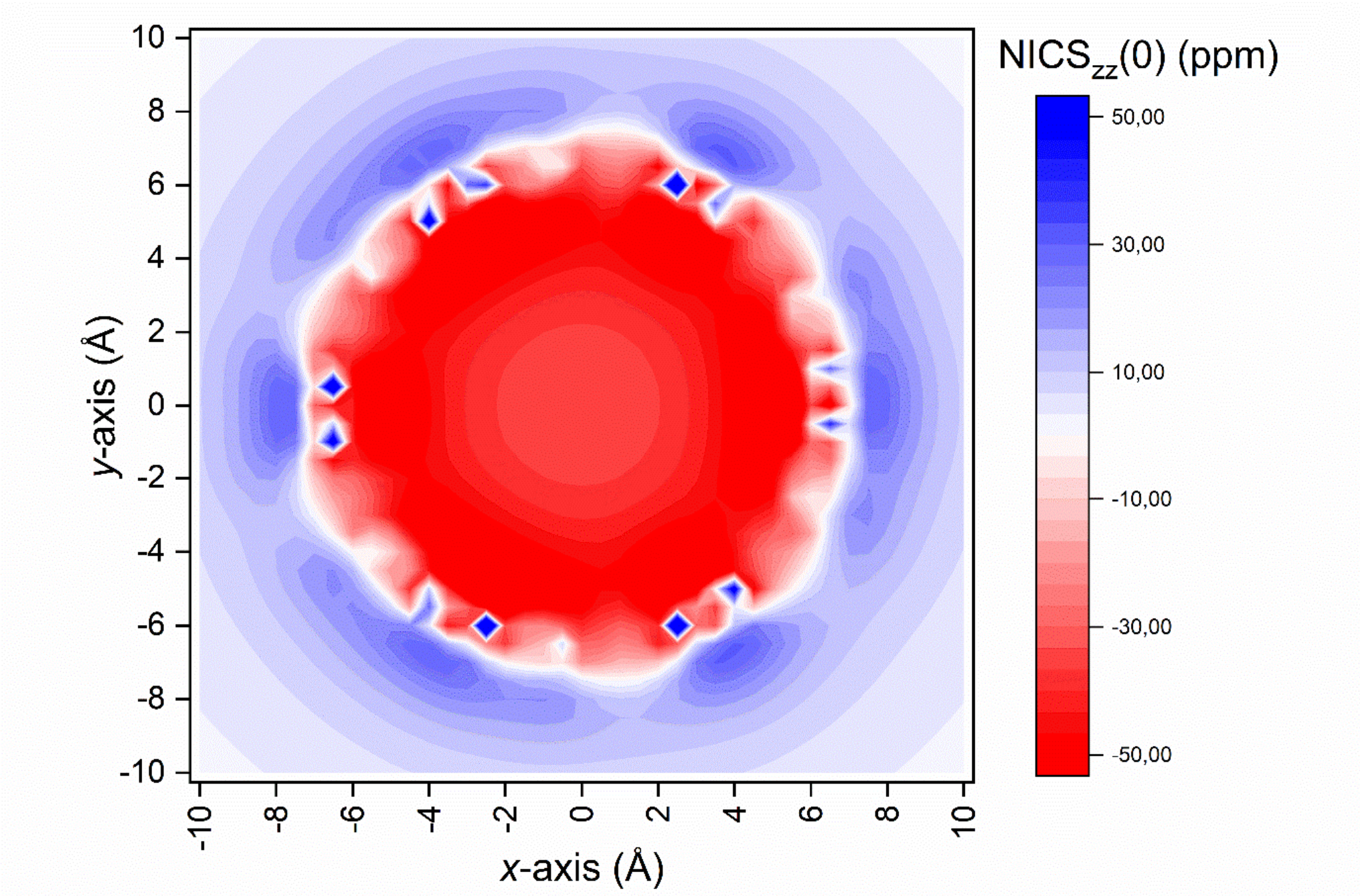

Figure S41. Calculated NICS$_{zz}$(0) heatmap in xy plane of //-[6]CPA **dianion**, calculated using the unrelaxed S_0 optimized minimum energy geometry of (neutral) //-[6]CPA. Calculation was performed using a 10x10 Å grid with 0.5 Å step, at B3LYP/6-311+G** level of theory.

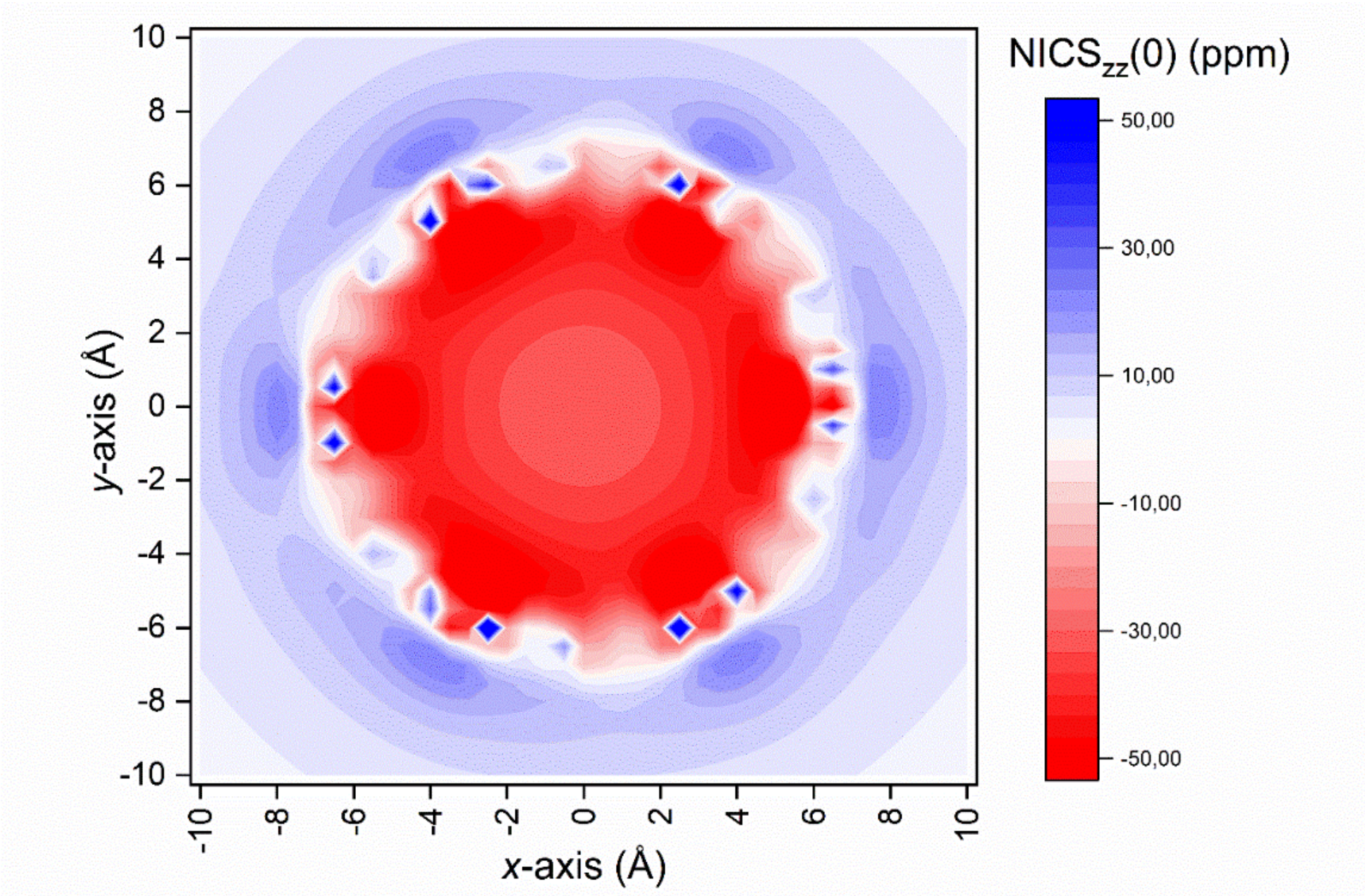

Figure S42. Calculated NICS$_{zz}$(0) heatmap in xy plane of //-[6]CPA **dication**, calculated using the unrelaxed S_0 optimized minimum energy geometry of (neutral) //-[6]CPA. Calculation was performed using a 10x10 Å grid with 0.5 Å step, at B3LYP/6-311+G** level of theory.

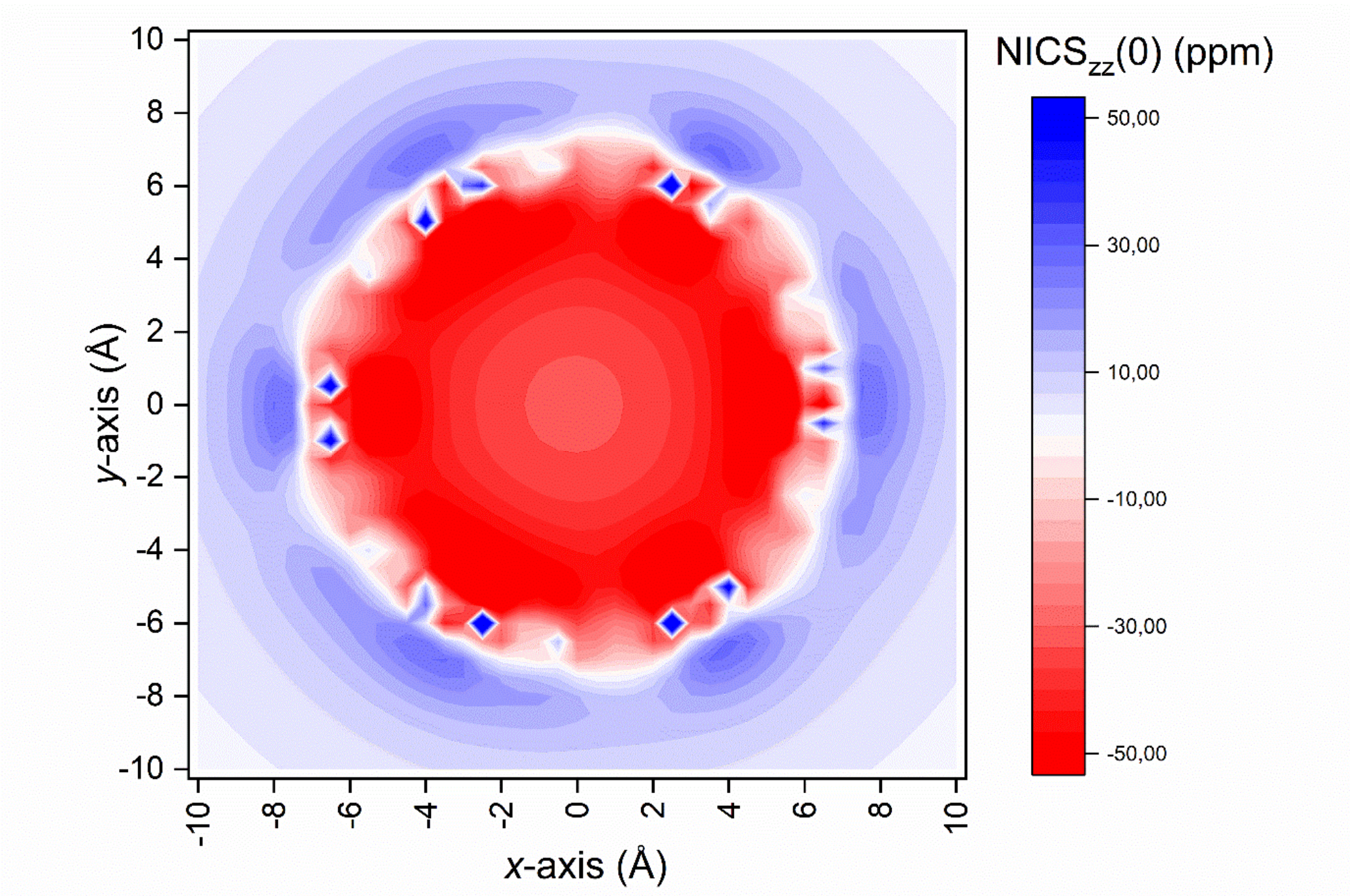

Figure S43. Calculated NICS$_{zz}$(0) heatmap in xy plane of **T_1** //-[6]CPA, calculated using the unrelaxed S_0 optimized minimum energy geometry of //-[6]CPA. Calculation was performed using a 10x10 Å grid with 0.5 Å step, at B3LYP/6-311+G** level of theory.

S8.2.7 Anisotropy of Induced Current Density (AICD Plots)

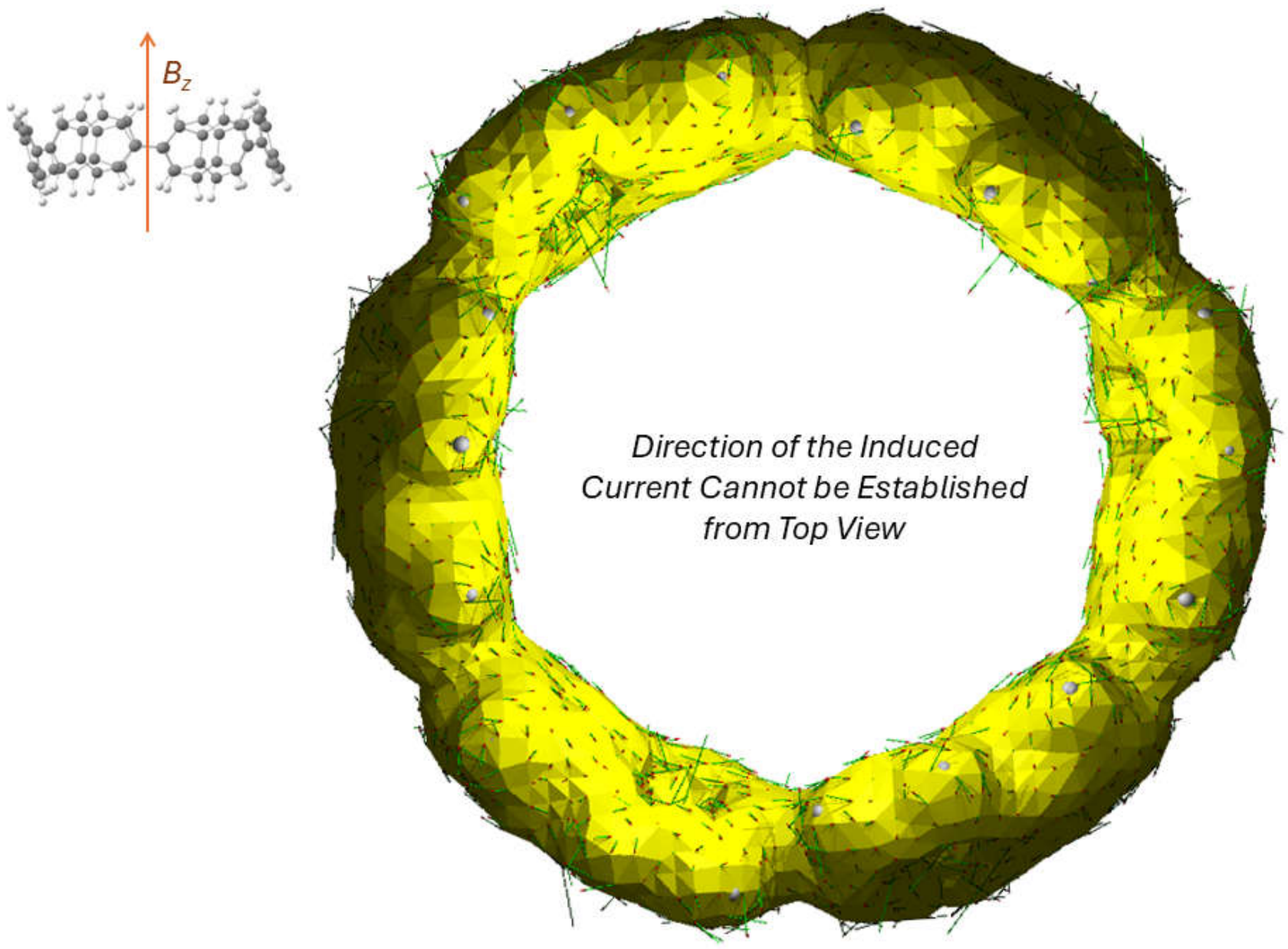

Figure S44. AICD plot of //-[6]CPA, where the external magnetic field is oriented in the z-axis (B_z), viewed from the top of the macrocycle (in the direction of the z-axis), calculated at B3LYP/6-311+G** level of theory, plotted at iso = 0.015.

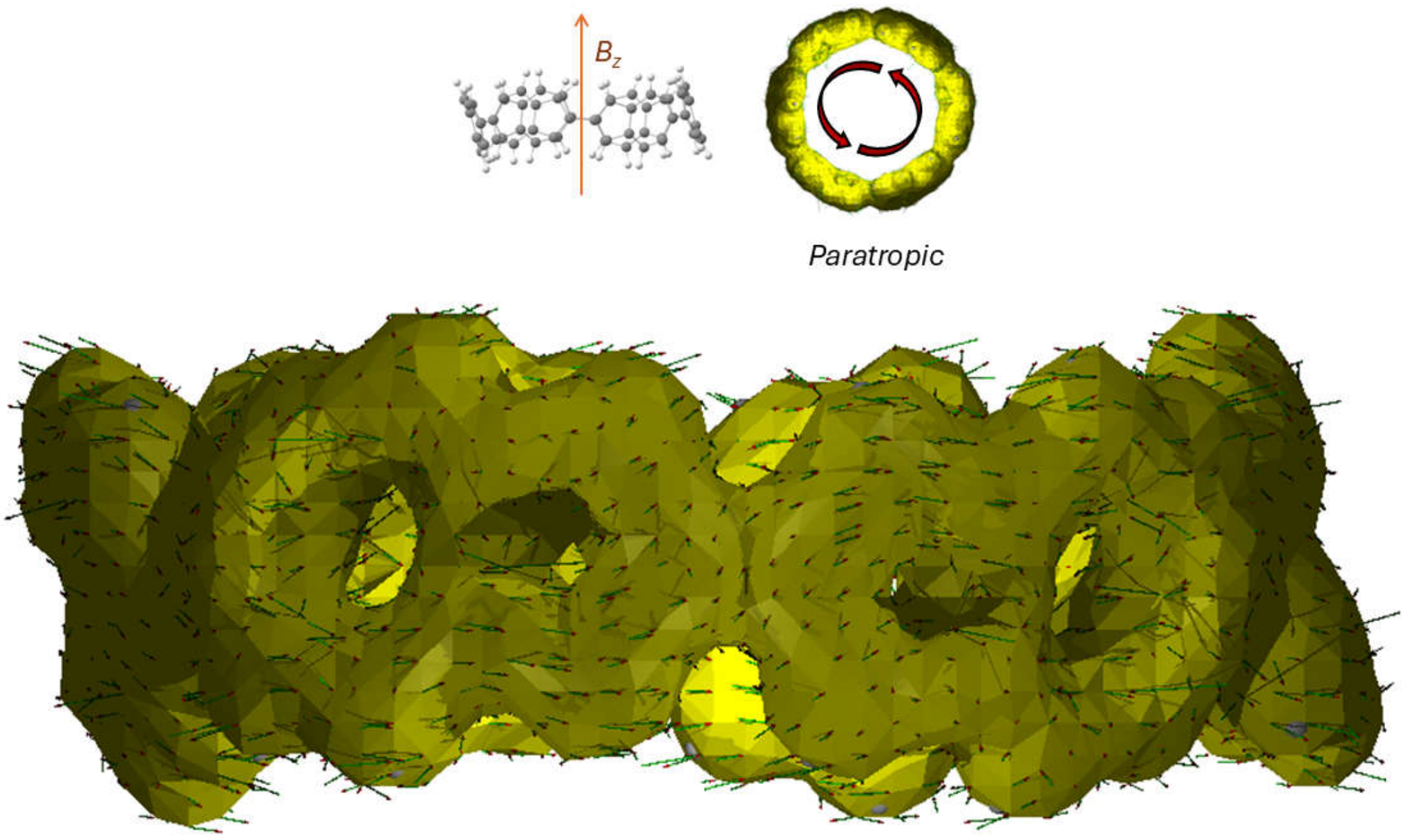

Figure S45. AICD plot of //-[6]CPA, where the external magnetic field is oriented in the z-axis (B_z), viewed in-plane of the macrocycle from the direction of the x-axis calculated at B3LYP/6-311+G** level of theory, plotted at iso = 0.015.

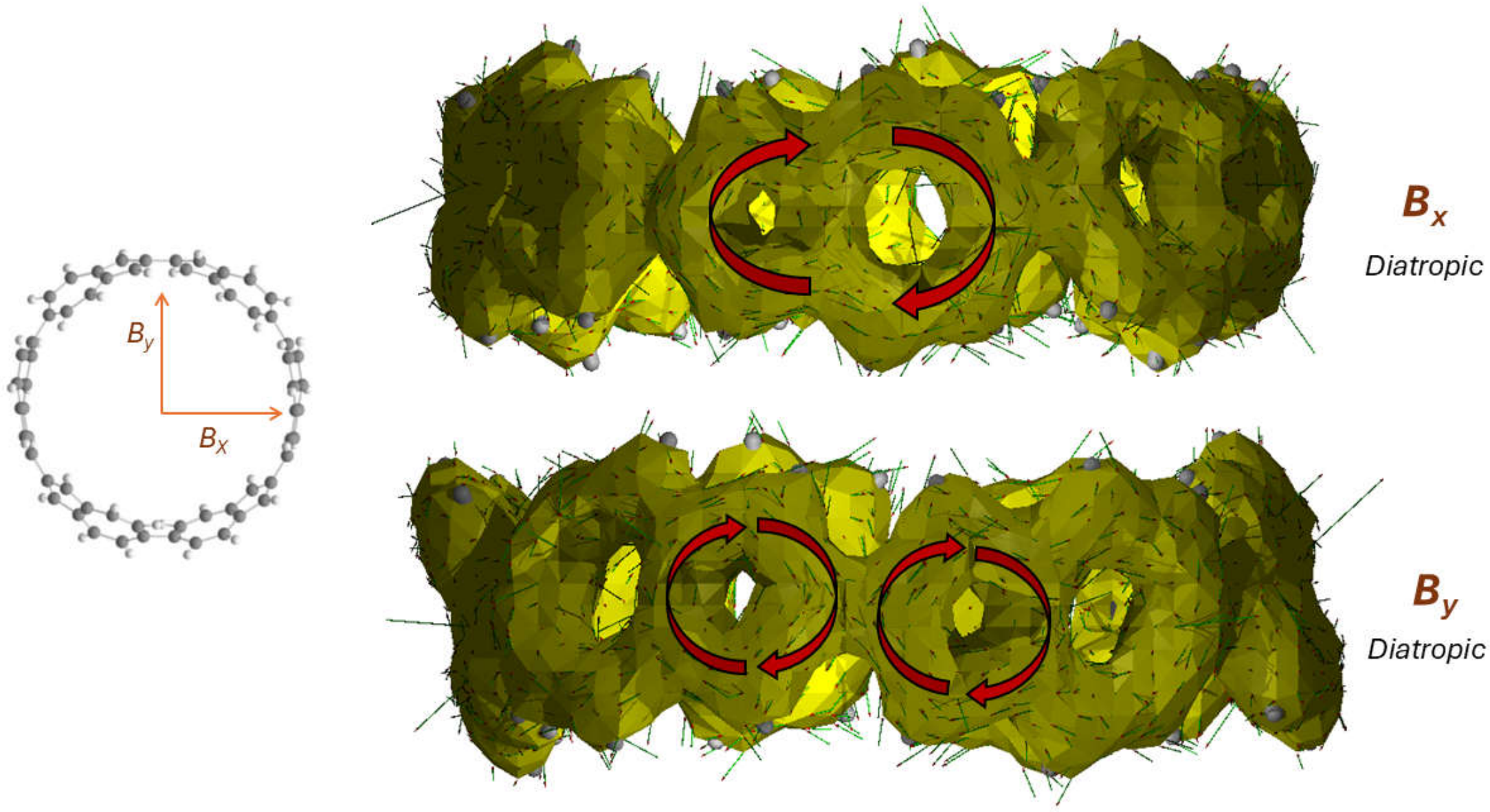

Figure S46. AICD plot of //-[6]CPA, where the external magnetic field is oriented in the x-axis (B_x) and the the y-axis (B_y) viewed in-plane of the macrocycle from the direction of the x-axis and the y-axis, respectively, calculated at B3LYP/6-311+G** level of theory, plotted at iso = 0.015.

S8.2.8 Isomerization Stabilization Energy (ISE)

We calculated the isomerization stabilization energy (ISE) of //-[6]CPA by applying the methyl-methylene isomerization protocol.[34] to a single azulene subunit. For this purpose, all reactants and products (Figure S47) were optimized at B3LYP/def2-TZVP level of theory and their minimum energy geometries were verified by an analytical hessian calculation, at the same level. The listed ISE values are the resulting electronic energies rather than Gibbs free energies, to avoid unnecessary contributions from strain relief. Nevertheless, because molecular strain and resonance energies cannot be fully decoupled by this approach, we highlight that isomerization reactions changing the hybridization of C2 and C6, particularly reactions 1a/1a‘ and 3b/3b‘, result in significant geometry change and thereby in outlier ISE values which cannot be used to evaluate resonance (de)stabilization. For comparison, the ISE values of pristine azulene have been previously reported by Slanina et. al.[35]

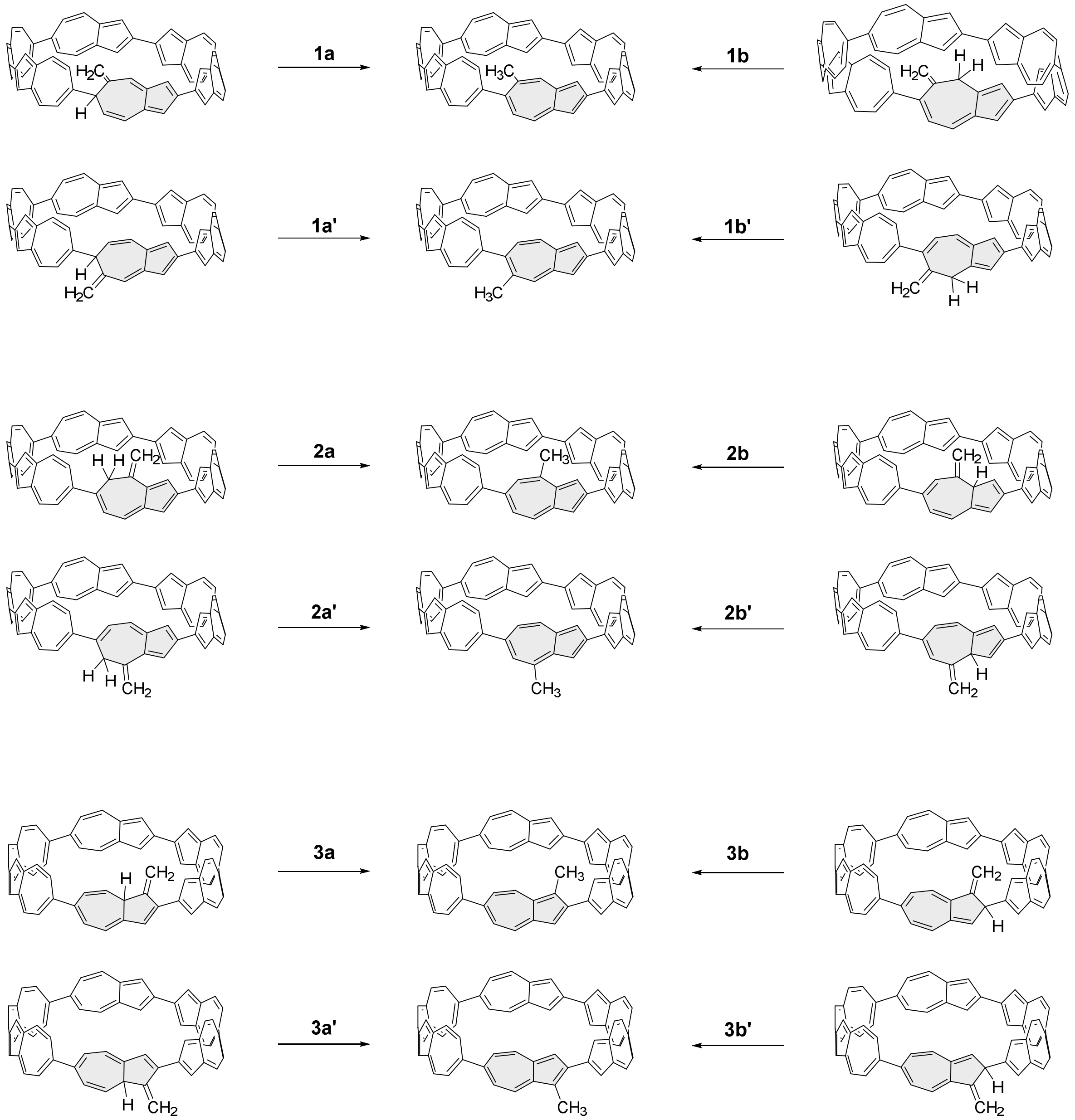

Figure S47. Isomerization reactions used for calculation of ISE of //-[6]CPA.

Table S7. Calculated ISE of //-[6]CPA, following the energies of reactant and products of reactions in Figure S47. Isomers were optimized and their single-point energies were calculated at B3LYP/def2-TZVP level of theory.

Reaction (Top Edge)	ISE kcal/mol	Reaction (Bottom Edge)	ISE kcal/mol
**1a**	-4.4[a]	**1a'**	0.2 [a]
**1b**	-16.2	**1b'**	-11.4
**2a**	-16.1	**2a'**	-21.7
**2b**	-16.3	**2b'**	-19.1
**3a**	-20.5	**3a'**	-19.3
**3b**	-12.8 [a]	**3b'**	-10.3[a]

[a]Outlier value skewed by significant geometry change. Cannot be used for evaluation of resonance (de)stabilization.

S8.2.9 Electron Delocalized Density in Bonds (EDDB)

Table S8. EDDB electron densities of //-[6]CPA in its optimized minimum-energy geometry at B3LYP/def2-TZVP level of theory; $EDDB_H$ – density of globally delocalized electrons in bonds of heavy atoms, $EDDB_F$ – density of delocalized bonds in bonds of fragments (local contributions), $EDDB_E$ – density of delocalized electrons in bonds of fragments (local+non-local contributions), $EDDB_P$ – density of delocalized electrons in bonds along a pathway. The “perimeter” and “azulene subunit” pathways are illustrated in Figure S48 and Figure S49.

**Index**	**Perimeter**	**Azulene Subunit**
$EDDB_H$	55.6	
$EDDB_E$	34.6	9.7
$EDDB_F$	20.1	8.3
$EDDB_P$	17.6	6.6
$EDDB_P$(cyc.)	7.5	5.5

$EDDB_H$

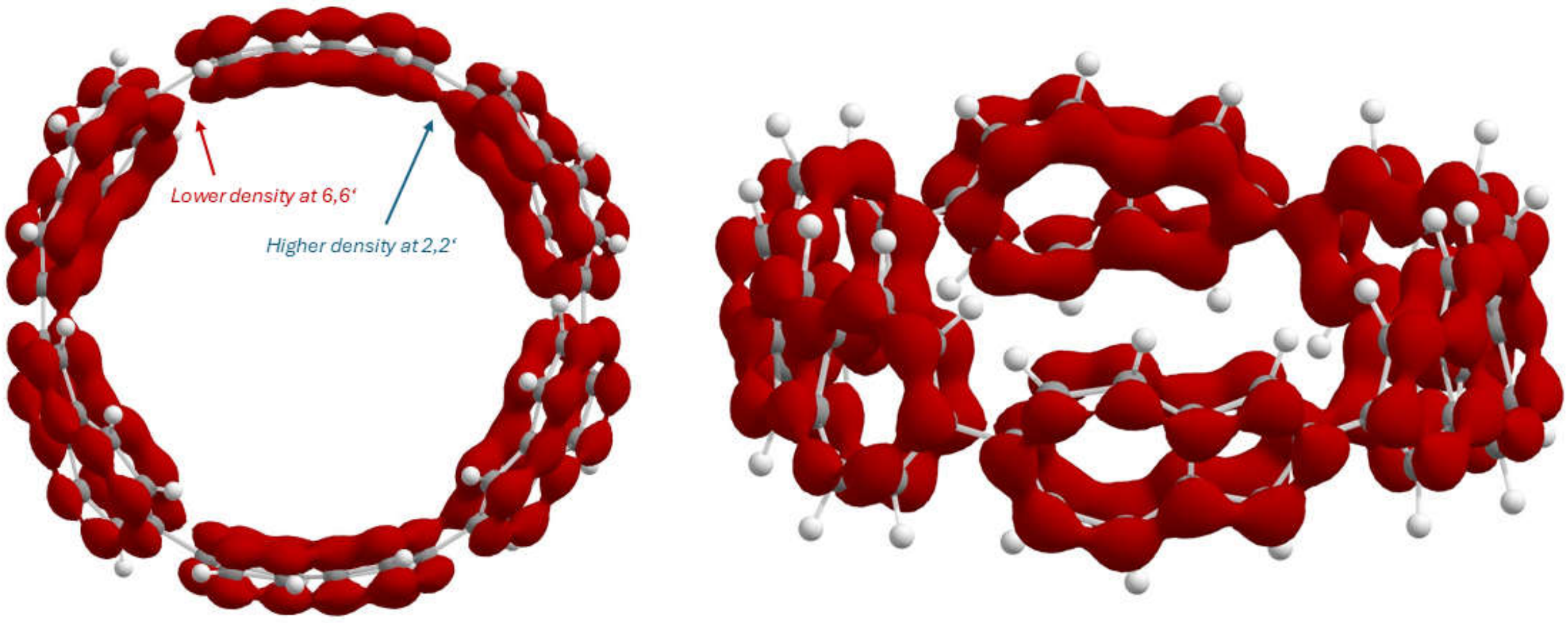

Figure S48. $EDDB_H$ (density of globally delocalized electrons in bonds of heavy atoms) plot of //-[6]CPA, calculated at B3LYP/def2-TZVP level of theory. The plot is rendered at iso = 0.0285, highlighting the lower density of delocalized electrons at C2**—**C2' and C6**—**C6' connections.

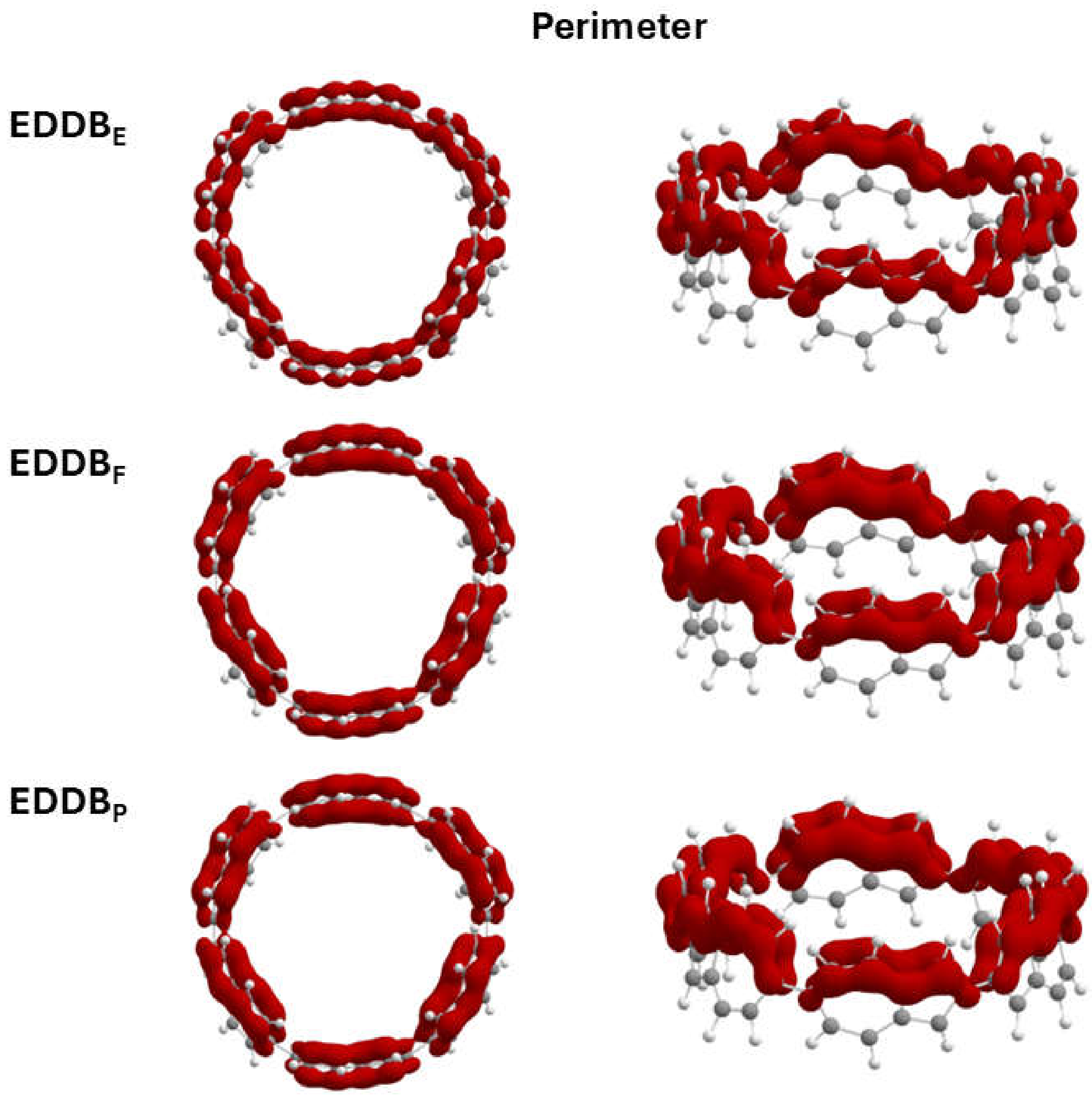

Figure S49. EDDB$_F$ – density of delocalized bonds in bonds of fragments (local contributions) plot, EDDB$_E$ – density of delocalized electrons in bonds of fragments (local+non-local contributions) plot, and EDDB$_P$ – density of delocalized electrons in bonds along a pathway plot of the "perimeter pathway" of //-[6]CPA, calculated at B3LYP/def2-TZVP level of theory. The plots are rendered at iso = 0.0285, 0.0225 and 0.0175, respectively, highlighting the lower density of delocalized electrons at C2—C2' and C6—C6' connections.

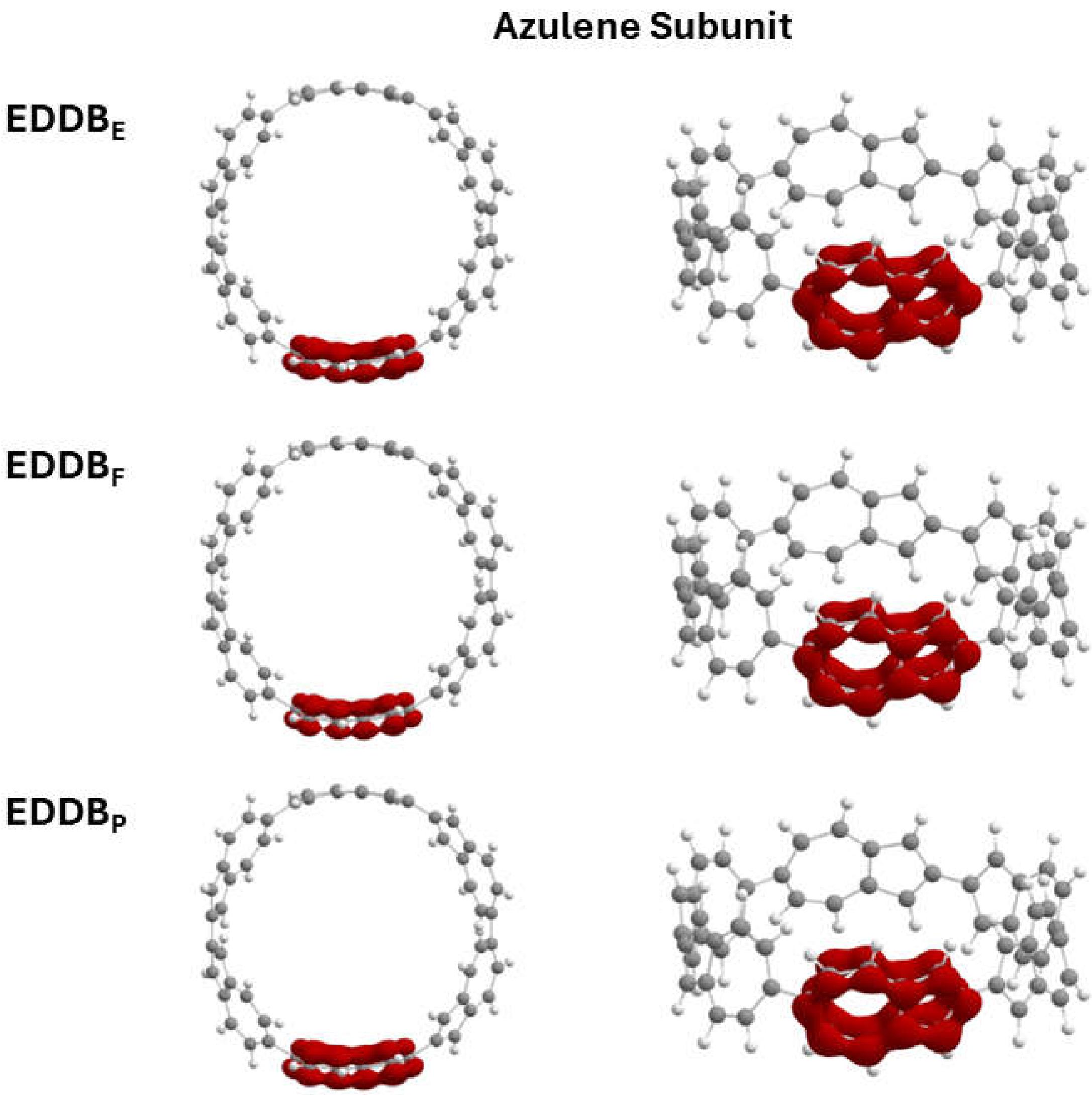

Figure S50. EDDB$_F$ – density of delocalized bonds in bonds of fragments (local contributions) plot, EDDB$_E$ – density of delocalized electrons in bonds of fragments (local+non-local contributions) plot, and EDDB$_P$ – density of delocalized electrons in bonds along a pathway plot of the “azulene subunit pathway” of //-[6]CPA, calculated at B3LYP/def2-TZVP level of theory. The plots are rendered at iso = 0.03, 0.03 and 0.0225, respectively.

S8.3 Computational Results on 2,2'-Biazulene and 6,6'-Biazulene

S8.3.1 Geometry of 2,2'- and 6,6'-Biazulene

To elucidate the conformation of biazulenyl subunits in [6]CPA, we performed a detailed conformer analysis of 2,2'- and 6,6'-biazulene, directly evaluating their isomerization profile by the *climbing image nudge elastic band* (CI-NEB) approach.[28]

Initial CREST conformer search, followed by geometry optimization and analytical hessian calculation, revealed one minimum energy geometry per each compound (Figure S51). Next, by renumbering the atoms, we produced product geometries of internal rotation, thereby allowing us to investigate the energy profile of this rotation by CI-NEB. Final isomerization energies were determined by saddle point optimization of the highest energy image, which was also verified by an analytical hessian calculation.

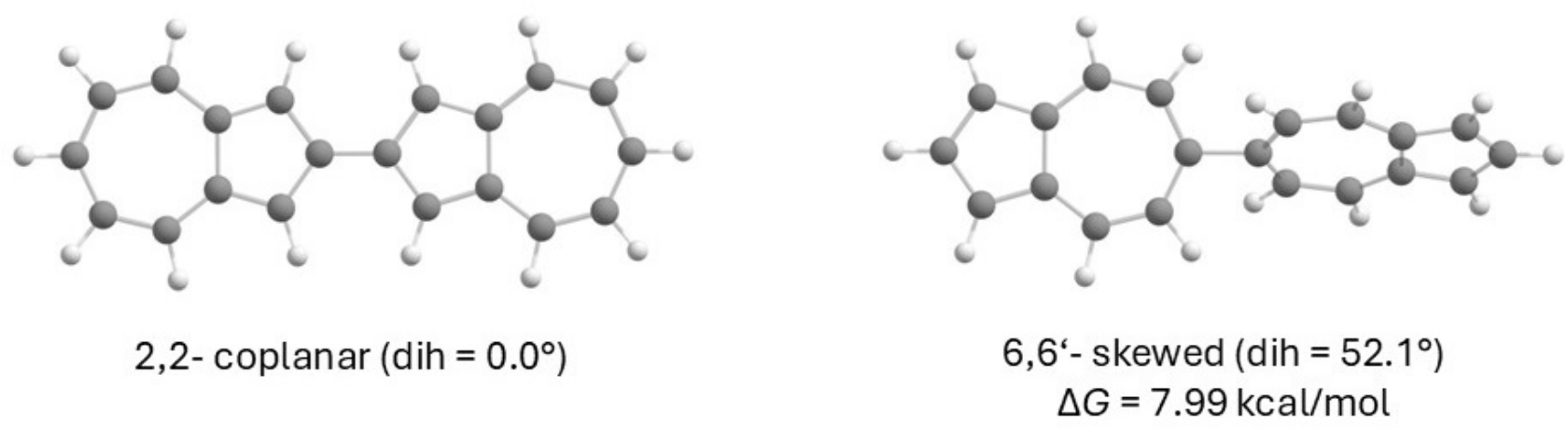

Figure S51. Minimum energy geometries of 2,2'- and 6,6'-biazulenyl, obtained by CREST Conformer Search (GFN2-xTB) and subsequent DFT optimization (B3LYP/def2-TZVP), incl. the relative Gibbs free energy of the higher-energy 6,6'-biazulene (ΔG).

2,2'-Biazulene

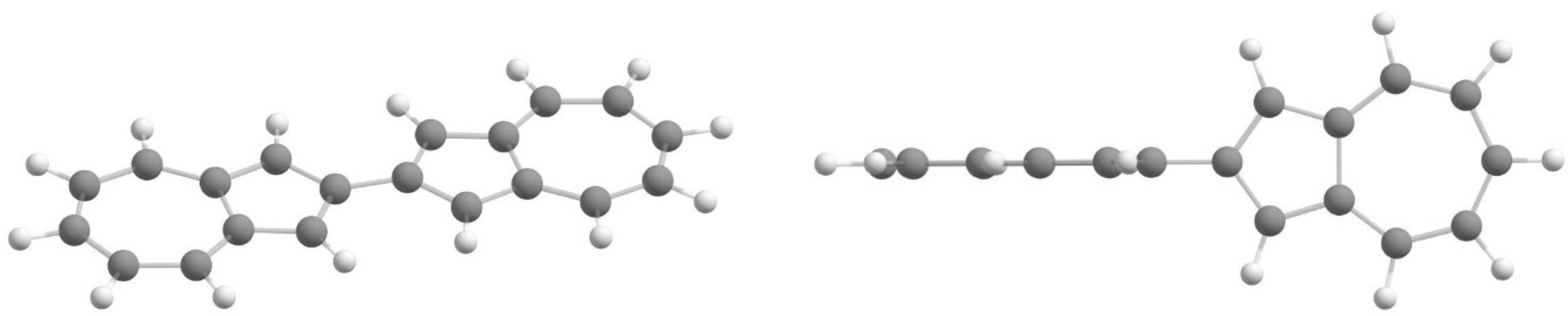

Figure S52. Left: Global minimum energy geometry, co-planar (0°); Right: Optimized saddle point geometry (90°, transition state, ΔE = 5.8 kcal/mol), both optimized at B3LYP/def2-TZVP level of theory.

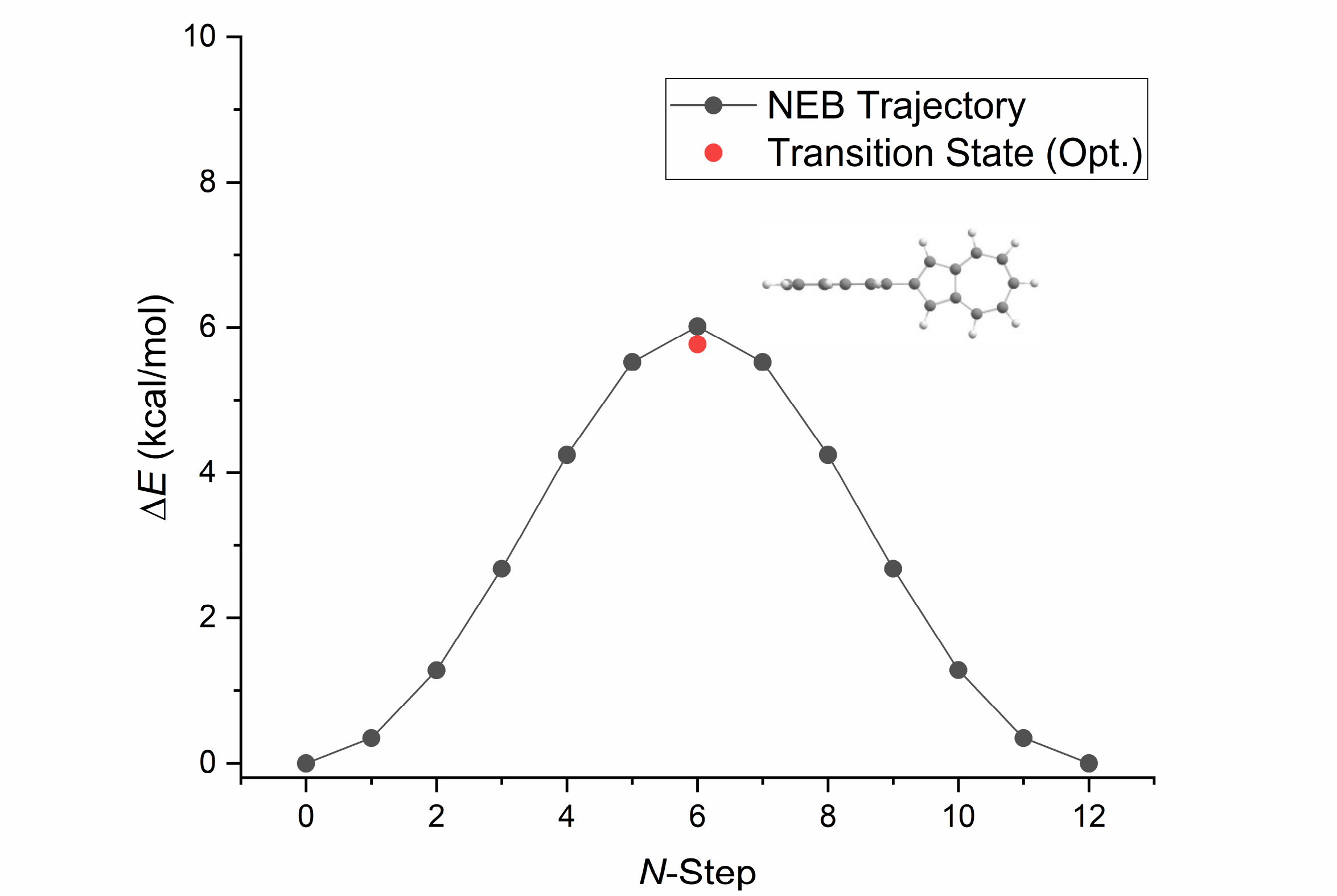

Figure S53. NEB-CI trajectory of isomerization of 2,2’-biazulene, used to identify and subsequently optimize the saddle point geometry (90°). NEB-CI trajectory and subsequent optimization were carried out at B3LYP/def2-TZVP level of theory.

6,6’-Biazulene

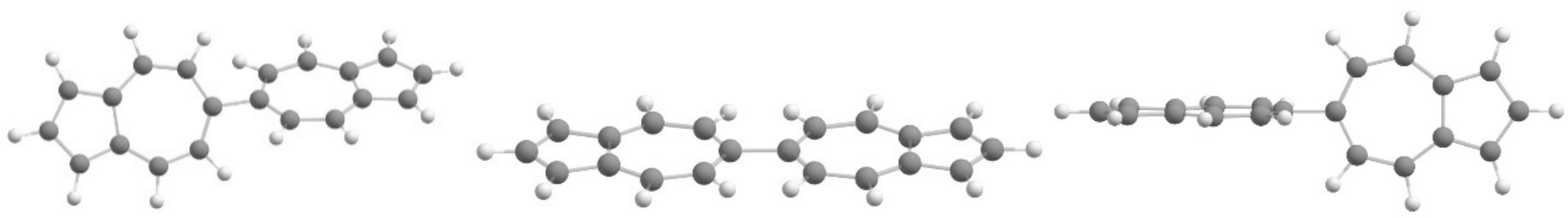

Figure S54. Left: Global minimum energy geometry, skewed; Middle: Optimized saddle point geometry 0° (transition state, Δ*E* = 7.6 kcal/mol); Right: Optimized saddle point geometry 0° transition state, Δ*E* = 1.4 kcal/mol), optimized at B3LYP/def2-TZVP level of theory.

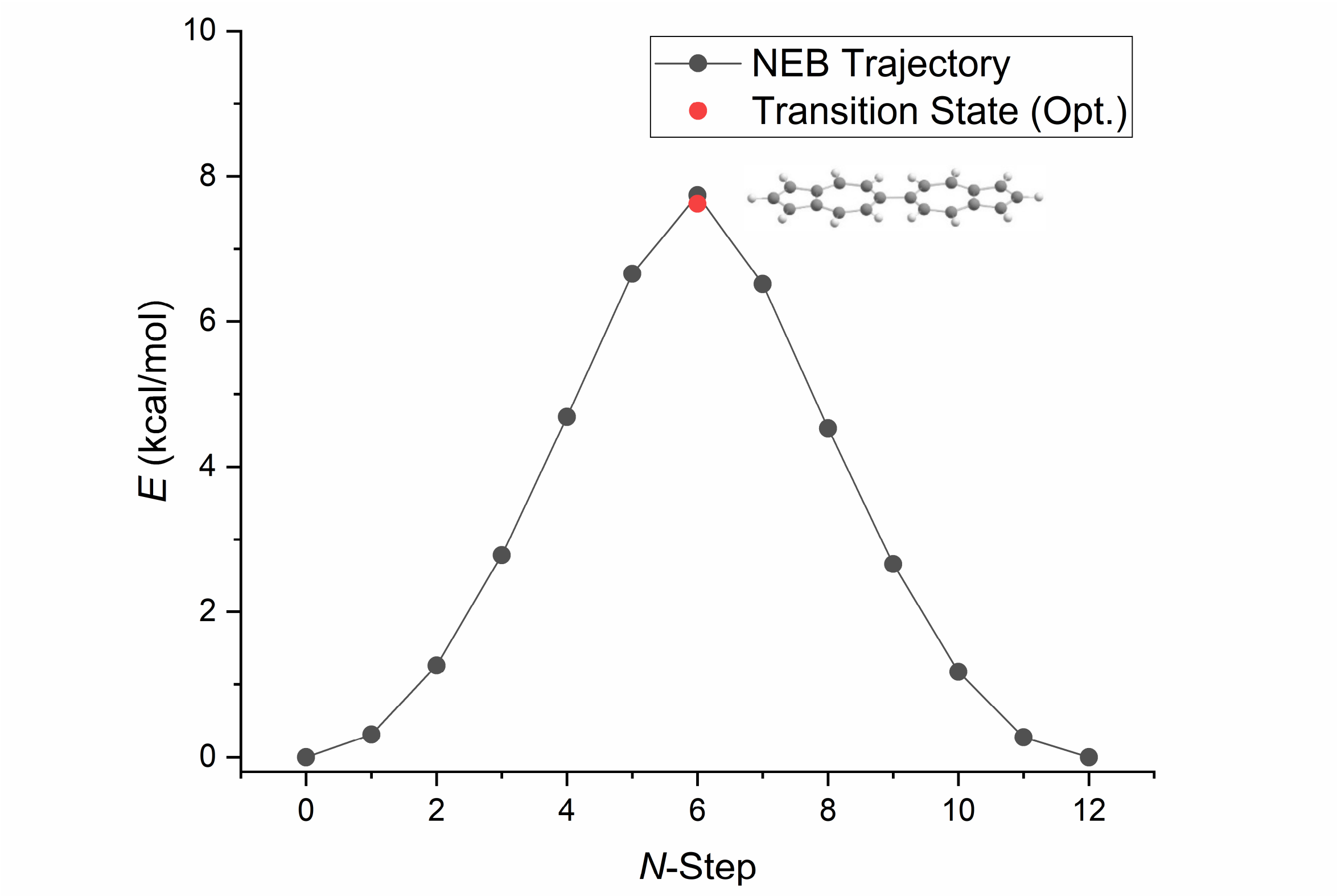

Figure S55. NEB-CI trajectory of isomerization of 6,6'-biazulene, used to identify and subsequently optimize the saddle point geometry (0°). NEB-CI trajectory and subsequent optimization were carried out at B3LYP/def2-TZVP level of theory.

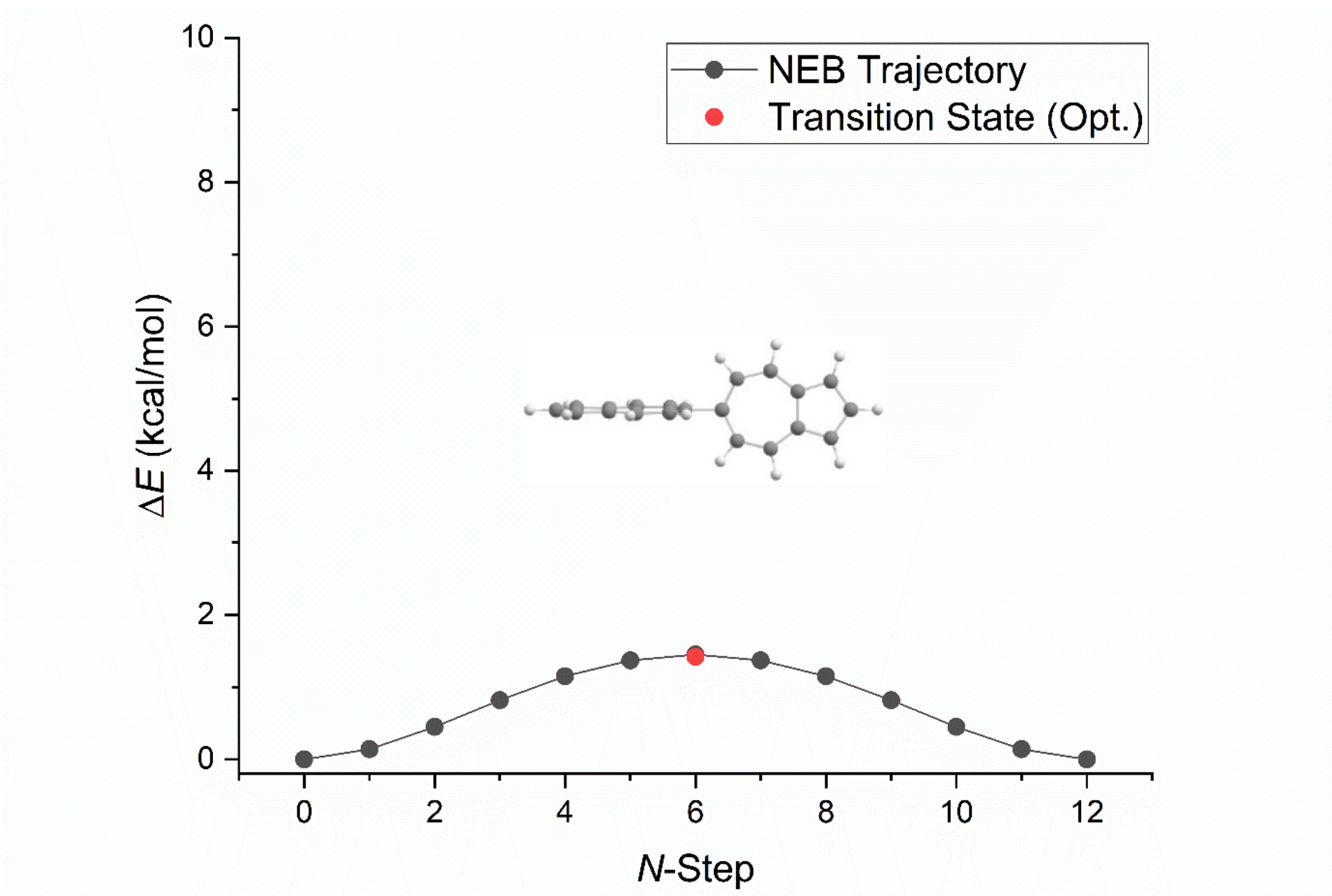

Figure S56. NEB-CI trajectory of isomerization of 6,6'-biazulene, used to identify and subsequently optimize the saddle point geometry (90°). NEB-CI trajectory and subsequent optimization were carried out at B3LYP/def2-TZVP level of theory.

S8.3.2 TD-DFT Results

Table S9. Calculated electronic transitions of 2,2'-biazulene at B3LYP/def2-TZVP level of theory, applying C-PCM implicit solvent model (chloroform).

Transition	Energy (cm^{-1})	Wavelength (nm)	f_{osc}	T^2 (au^2)	T_X (au)	T_Y (au)	T_Z (au)
1	16978	589	0.0E+00	0.000	0.000	0.000	0.000
2	18385	544	2.5E-02	0.445	0.000	-0.667	0.000
3	22419	446	9.5E-01	13.903	3.729	0.000	0.000
4	25400	394	4.6E-04	0.006	0.000	-0.078	0.000
5	26730	374	0.0E+00	0.000	0.000	0.000	0.000
6	27975	358	0.0E+00	0.000	0.000	0.000	0.000
7	30080	333	8.6E-01	9.435	-3.072	0.000	0.000
8	30774	325	1.0E-09	0.000	0.000	0.000	0.000
9	31122	321	0.0E+00	0.000	0.000	0.000	0.000
10	32198	311	0.0E+00	0.000	0.000	0.000	0.000
11	33198	301	1.3E-01	1.269	0.000	-1.127	0.000
12	33495	299	1.3E+00	12.868	-3.587	0.000	0.000
13	39772	251	0.0E+00	0.000	0.000	0.000	0.000
14	41820	239	2.3E-02	0.178	0.000	-0.421	0.000
15	42181	237	0.0E+00	0.000	0.000	0.000	0.000

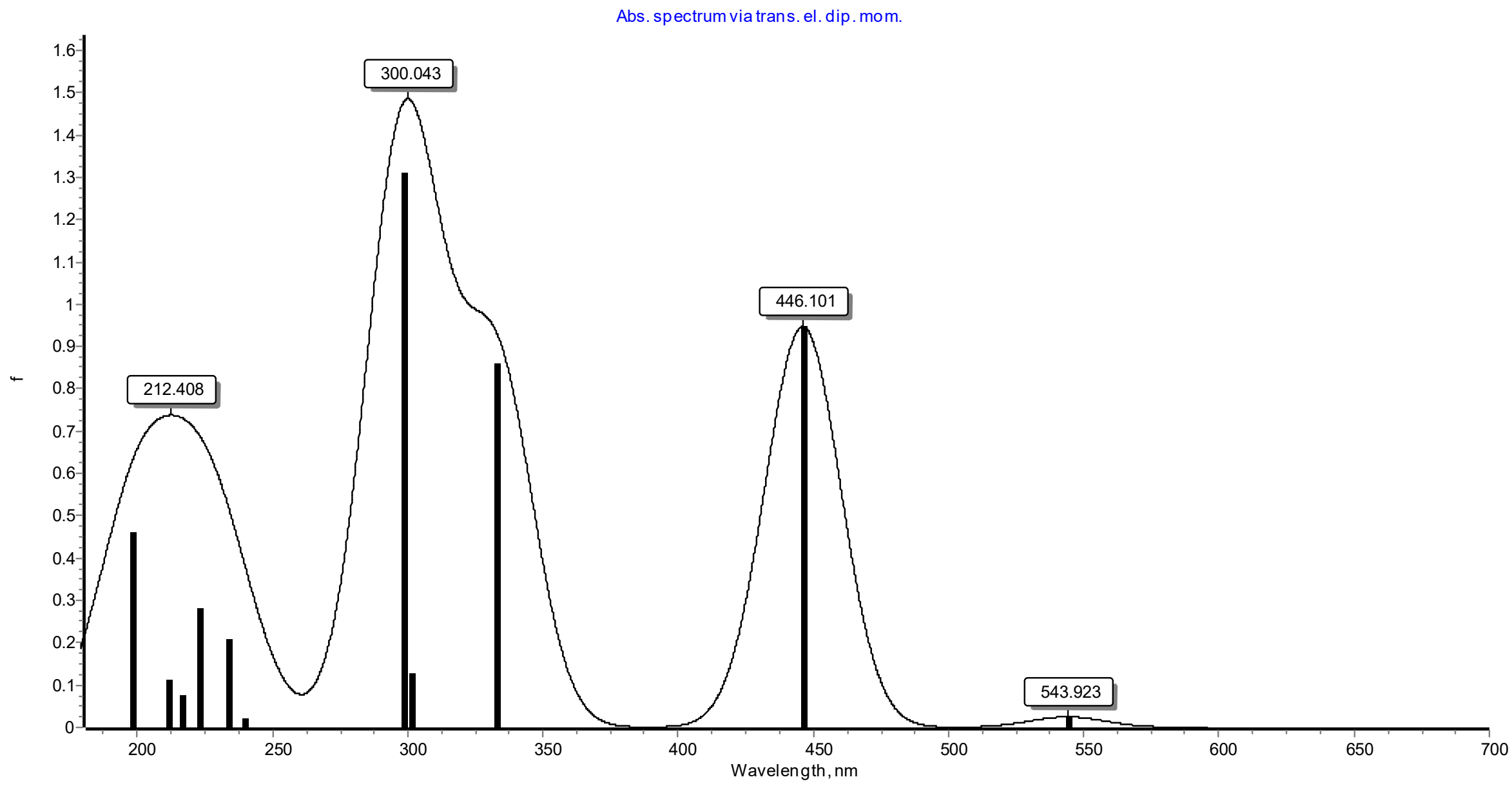

Figure S57. Plotted oscillator strengths calculated electronic transitions of 2,2'-biazulene at B3LYP/def2-TZVP level of theory, applying C-PCM implicit solvent model (chloroform).

Table S10. Calculated electronic transitions of 6,6'-biazulene at B3LYP/def2-TZVP level of theory, applying C-PCM implicit solvent model (chloroform).

Transition	Energy (cm^{-1})	Wavelength (nm)	f_{osc}	T^2 (au^2)	T_X (au)	T_Y (au)	T_Z (au)
1	17523	571	1.44E-02	0.270	0.000	0.520	0.000
2	17884	559	3.49E-03	0.064	0.000	0.000	0.253
3	24979	400	6.32E-04	0.008	0.000	0.000	-0.091
4	25348	395	2.07E-03	0.027	0.000	-0.164	0.000
5	25778	388	4.17E-01	5.324	-2.307	0.000	0.000
6	28868	346	0.00E+00	0.000	0.000	0.000	0.000
7	29907	334	3.22E-01	3.539	1.881	0.000	0.000
8	30144	332	1.00E-09	0.000	0.000	0.000	0.000
9	31053	322	0.00E+00	0.000	0.000	0.000	0.000
10	31919	313	1.35E+00	13.938	3.733	0.000	0.000
11	34655	289	8.42E-02	0.800	0.000	-0.894	0.000
12	35559	281	2.00E-02	0.186	0.000	0.000	0.431
13	38409	260	1.15E+00	9.843	-3.137	0.000	0.000
14	38956	257	1.00E-09	0.000	0.000	0.000	0.000
15	39081	256	6.13E-03	0.052	0.000	0.000	0.227

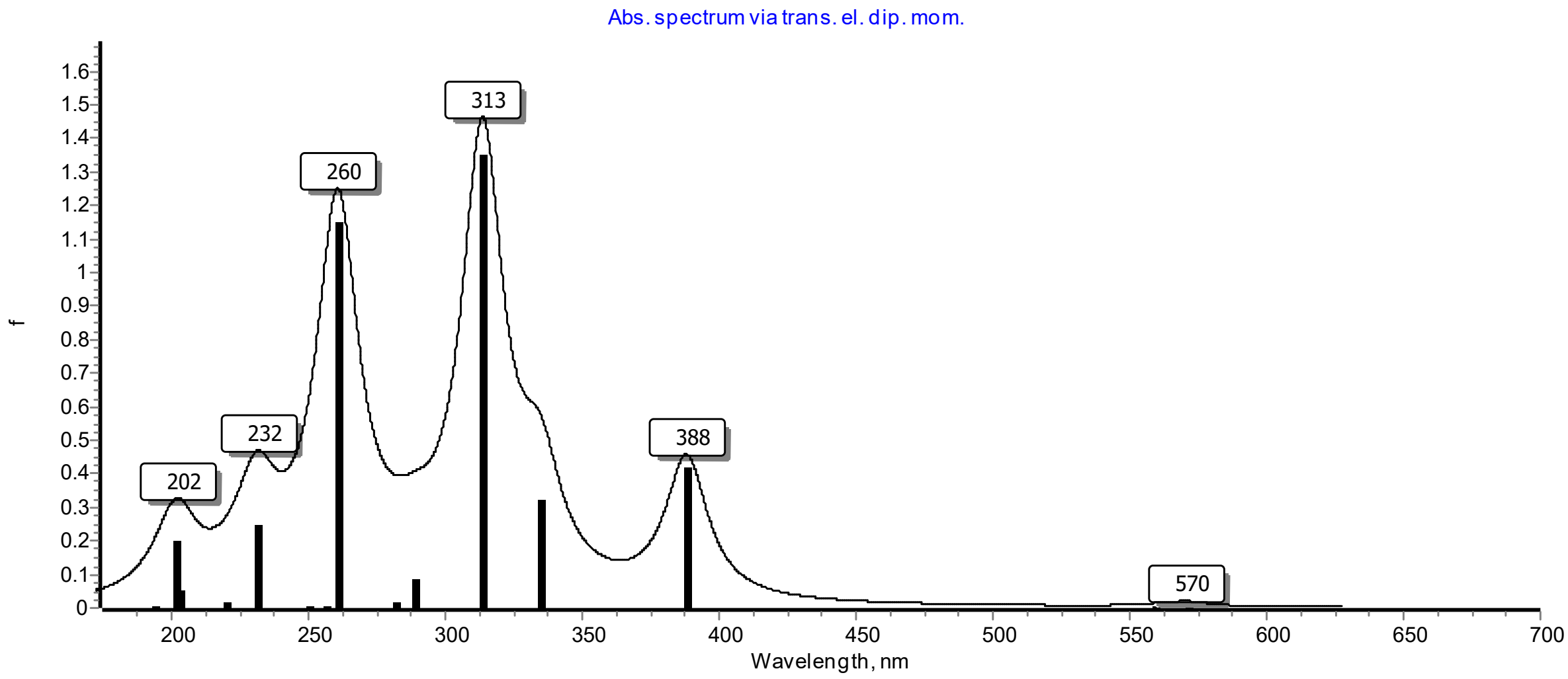

Figure S58. Plotted oscillator strengths calculated electronic transitions of 6,6'-biazulene at B3LYP/def2-TZVP level of theory, applying C-PCM implicit solvent model (chloroform).

S8.3.3 Geometric and Electronic Delocalization Indices (HOMA, FLU, MCI)

Table S11. Harmonic oscillator model of aromaticity (HOMA) index of 2,2'-biazulene (22') and 6,6'-biazulene (66') in their minimum energy geometries, within their 5-member (*5M*), 7-member (*7M*) and 10-member (*10M*) circuits. The geometries were optimized at B3LYP/def2-TZVP level of theory. The conformers are denoted by their dihedral angle.

Ring (HOMA)	22' (0°)	66' 52°
7M	0.58	0.60
5M	0.35	0.42
10M	0.96	0.97

Table S12. Multicenter bond index (MCI) of 2,2'-biazulene (22') and 6,6'-biazulene (66') in their minimum energy geometries, within their 5-member (*5M*), 7-member (*7M*) and 10-member (*10M*) circuits, at B3LYP/6-311+G** level of theory. The geometries were optimized at B3LYP/def2-TZVP level of theory. The conformers are denoted by their dihedral angle.

Ring (MCI)	22' 0°	66' 52°
7M	0.026	0.025
5M	0.042	0.049

10M	0.029	0.032

Table S13. Aromatic fluctuation index (FLU) of 2,2'-biazulene (22') and 6,6'-biazulene (66') in their minimum energy geometries, within their 5-member (*5M*), 7-member (*7M*) and 10-member (*10M*) circuits, at B3LYP/6-311+G** level of theory. The geometries were optimized at B3LYP/def2-TZVP level of theory. The conformers are denoted by their dihedral angle.

**Ring (FLU)**	**22' 0°**	**66' 52°**
7M	0.010	0.010
5M	0.016	0.015
10M	0.0007	0.0002

S8.3.4 Magnetic (Shielding) Indices (NICS)

Table S14. Overview of calculated $NICS_{zz}$ values of 2,2'-biazulene (22') and 6,6'-biazulene (66') in their minimum energy geometries, at/tangentially to the centroid of their 5-member (*5M*) ring, at B3LYP/6-311+G** level of theory. The geometries were optimized at B3LYP/def2-TZVP level of theory. The conformers are denoted by their dihedral angle.

**$NICS_{zz}$(*N*), 5M**	**22' 0°**	**66' 52°**
0.0	-23.6	-29.7
1.0	-43.6	-47.9
1.7	**-26.2**	**-29.3**

Table S15. Overview of calculated $NICS_{zz}$ values of 2,2'-biazulene (22') and 6,6'-biazulene (66') in their minimum energy geometries, at/tangentially to the centroid of their 7-member (*7M*) ring, at B3LYP/6-311+G** level of theory. The geometries were optimized at B3LYP/def2-TZVP level of theory. The conformers are denoted by their dihedral angle.

**$NICS_{zz}$(*N*), 7M**	**22' 0°**	**66' 52°**
0.0	-4.1	-7.7
1.0	-16.4	-19.6
1.7	**-15.8**	**-17.9**

S8.3.5 Anisotropic Induced Current Density (AICD Plots)

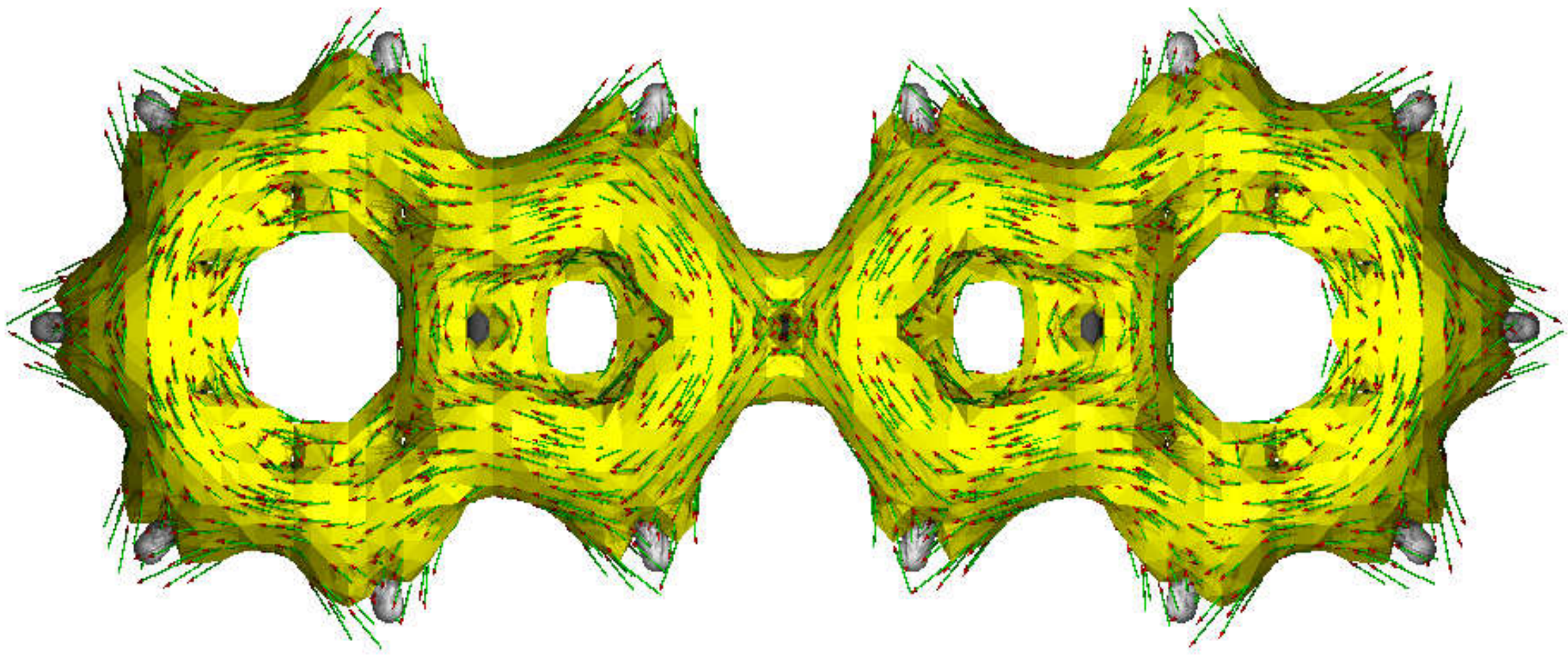

Figure S59. AICD plot of 2,2’-biazulene at minimum energy geometry, calculated at B3LYP/6-311+G** level of theory, plotted at iso = 0.04.

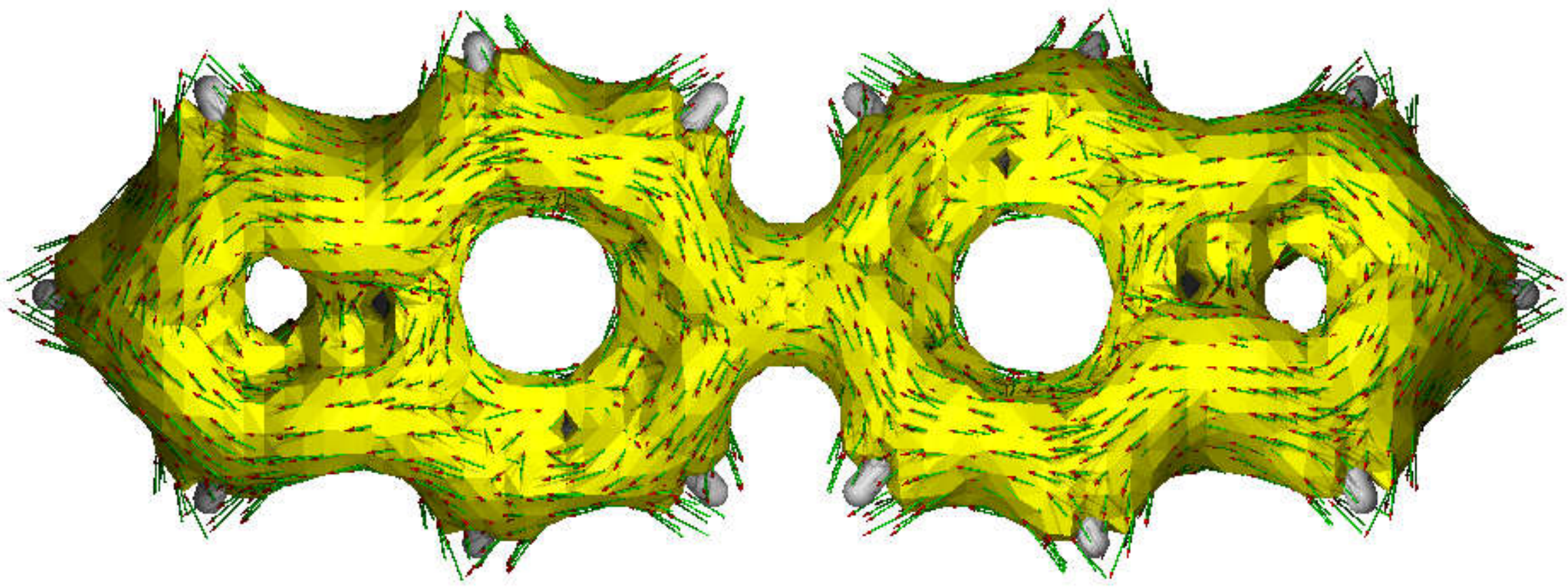

Figure S60. AICD plot of 6,6’-biazulene at minimum energy geometry, calculated at B3LYP/6-311+G** level of theory, plotted at iso = 0.04.

S8.3.6 Isomerization stabilization energy (ISE)

We calculated the isomerization stabilization energy (ISE), applying the methyl-methylene protocol.[34] to a single azulene subunit of 2,2’- and 6,6’-biazulene. For this purpose, all reactants and products (Figures S57–S58) were optimized at B3LYP/def2-TZVP level of theory and their minimum energy geometries were verified by an analytical hessian calculation, at the same level. The listed ISE values are the resulting electronic energies rather than Gibbs free energies. The isomerization reactions were numbered so that they can be directly compared to those of [6]CPA.

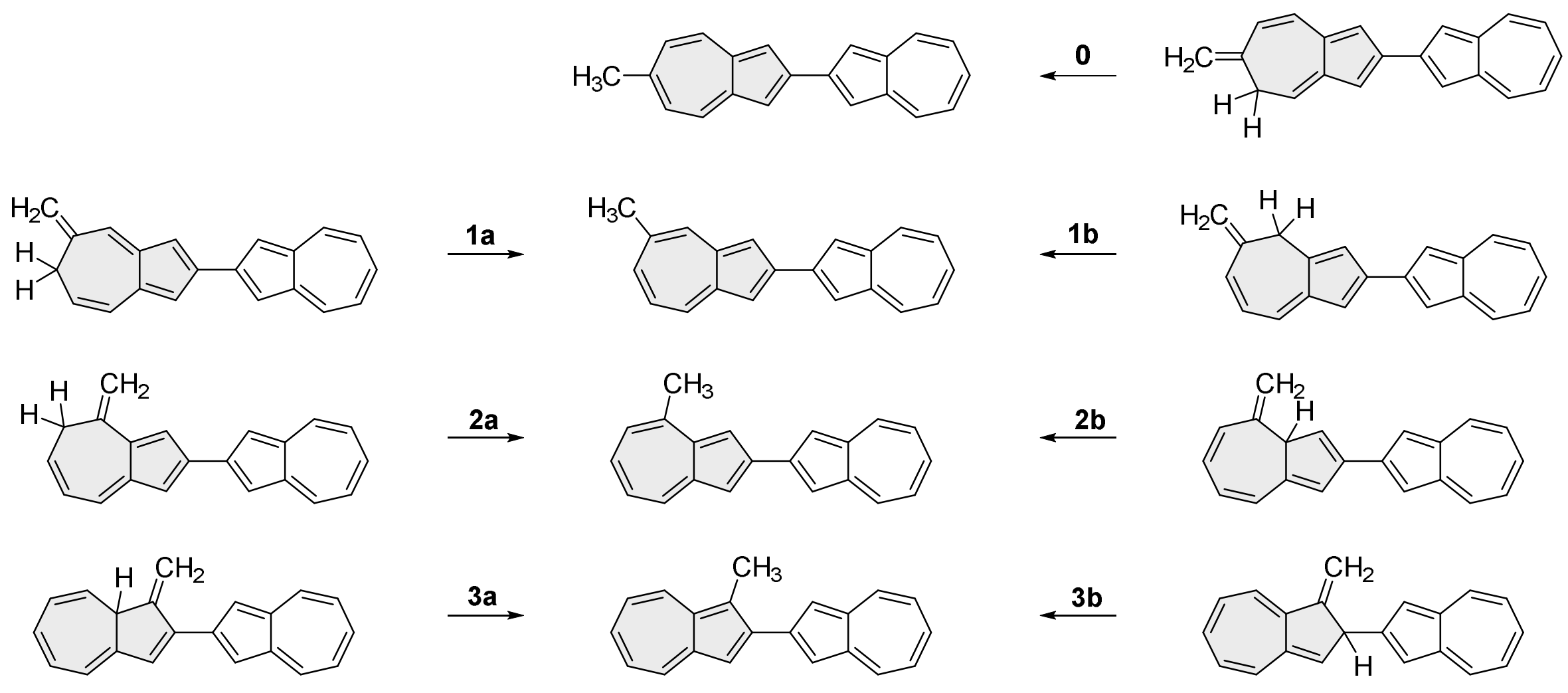

Figure S61. Isomerization reactions used for calculation of ISE of 2,2’-biazulene.

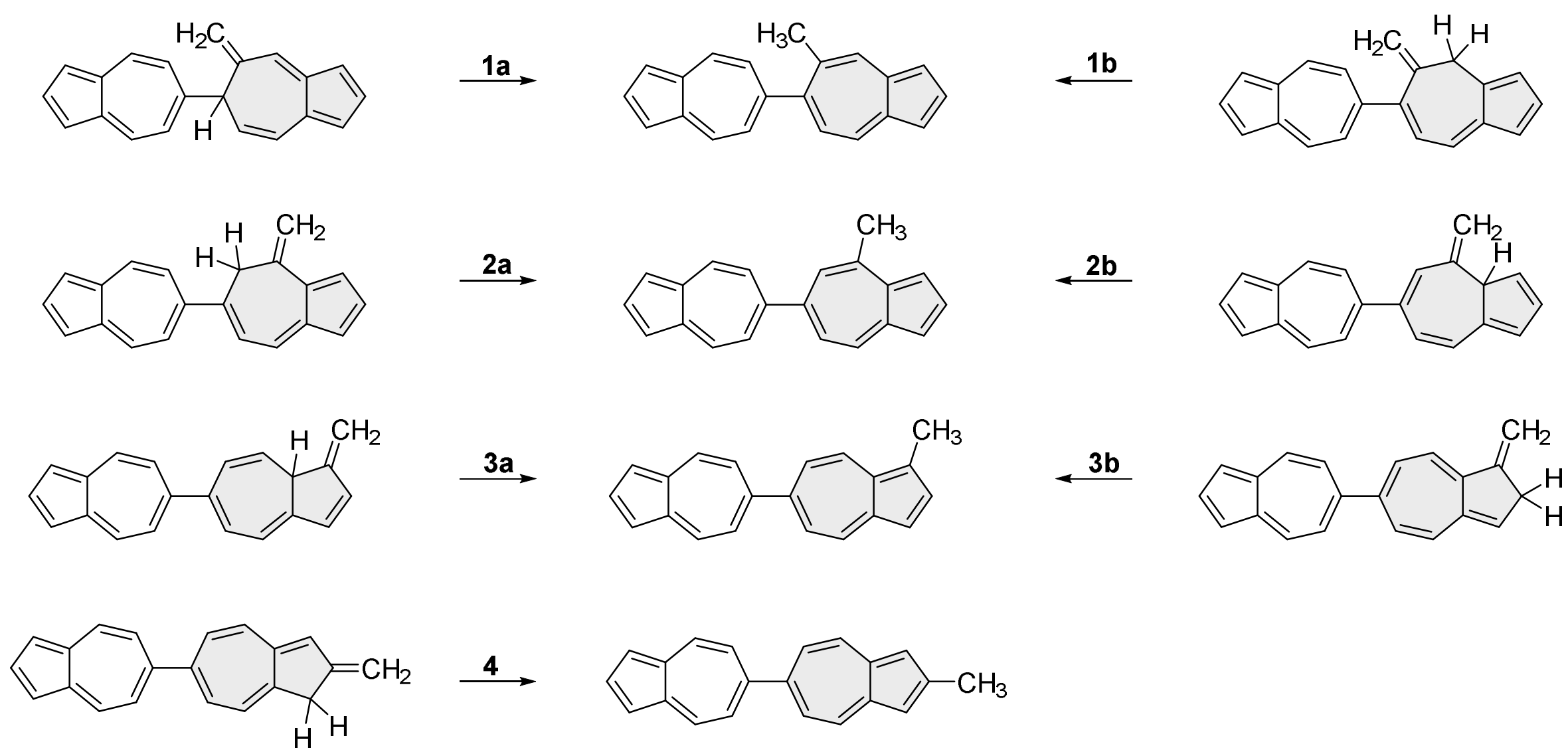

Figure S62. Isomerization reactions used for calculation of ISE of 6,6’-biazulene.

Table S16. Calculated ISE of 2,2'-biazulene (2,2') and 6,6'-biazulene (6,6'), following the energies of reactant and products of reactions in Figure S61–S62. Isomers were optimized and their single-point energies were calculated at B3LYP/def2-TZVP level of theory.

**Reaction**	**ISE (2,2')** kcal/mol	**ISE (6,6')** kcal/mol
**0**	-23.4	-
**1a**	-22.5	-23.6
**1b**	-22.5	-20.7
**2a**	-24.6	-22.8
**2b**	-27.1	-25.7
**3a**	-21.7	-20.8
**3b**	-26.8	-20.1
**4**	-	-16.6

S8.4 Extended Discussion TD-DFT

S8.4.1 Azulene

Assignment of UV-vis spectra of azulene is well established and can be qualitatively reproduced by a TD-DFT calculation at TD-B3LYP/def2-TZVP level of theory, using only the first two electronic transitions. Azulene's UV-vis absorption spectra (here shown in cyclohexane, Figure S63)[35] are dominated by two absorption bands: a broad, weak S_0–S_1 transition with a maximum around 600 nm^{-1} and sharp, vibrationally resolved S_0–S_2 transition with a maximum at 338 nm.

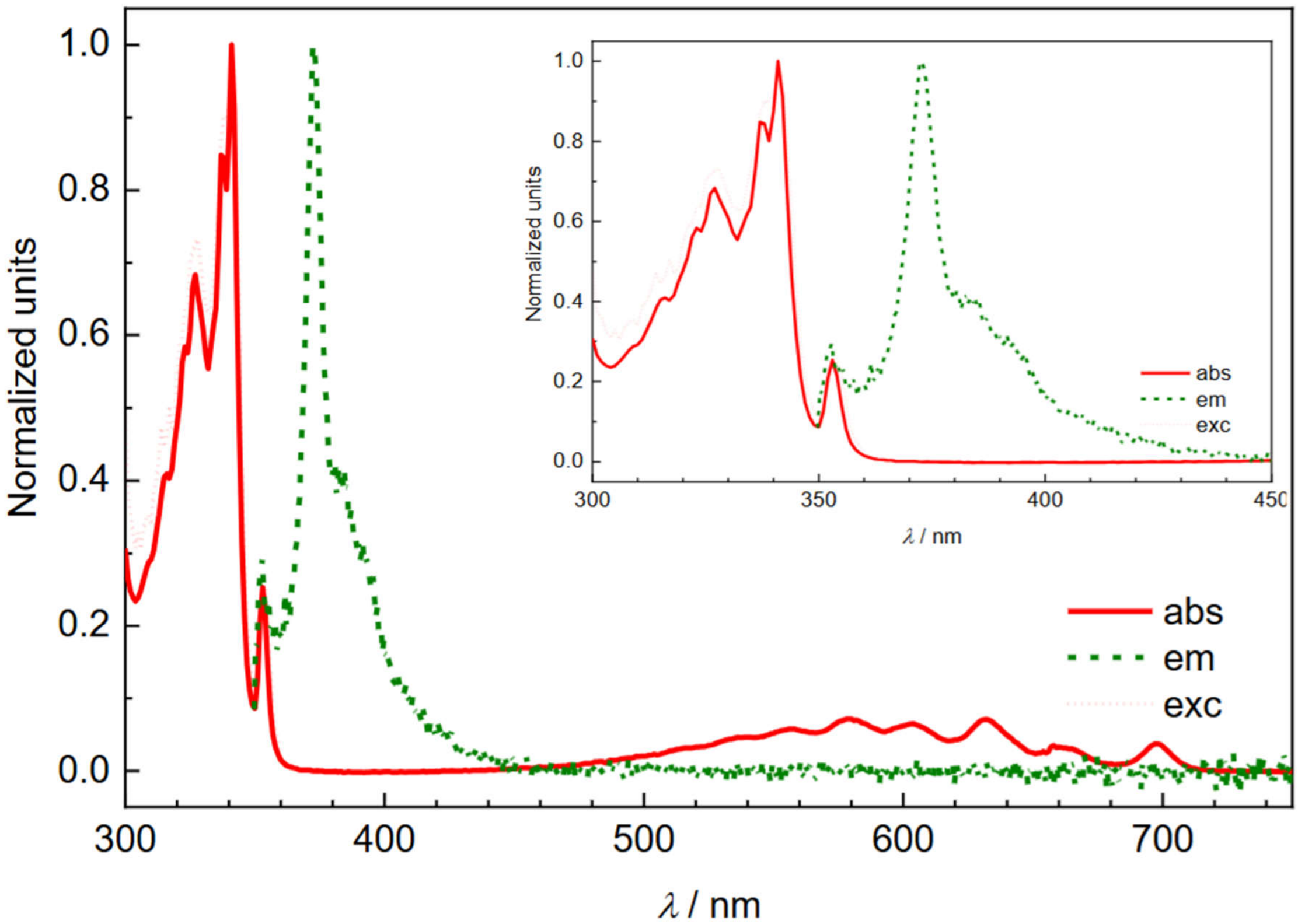

Figure S63. UV-Vis Absorption, Emission and Excitation spectrum of pristine azulene, recorded in cyclohexane.[35]

Azulene's first UV-vis absorption band is assigned to the first, HOMO→LUMO electronic transition. Owing to the *nonalternant* topology of azulene, these two frontier orbitals are non-symmetrically distributed in space and have a very low spatial overlap (Figure S64), thereby leading to a significant charge transfer character. In turn, the Coulombic repulsion in S_1 is lower than in π–π^* transitions of similarly large *alternant* hydrocarbons, e.g. naphthalene, leading to an anomalously red-shifted first transition energy.[36]However, the low spatial overlap of HOMO and LUMO also results in a low transition dipole moment, thereby rendering the intensity of the first absorption band very weak.

Azulene's second UV-vis absorption band is assigned to its second electronic transition. This transition is, in the basis of ground state molecular orbitals, assigned to a near-degenerate linear combination of HOMO→LUMO+1 and HOMO–1→LUMO. Because these orbitals have a large spatial overlap and are more evenly distributed in space, the intensity of the second UV-vis absorption band is higher and, moreover, the band has a sharp, well-resolved vibrational structure.

Thus, we can distinguish two *types* of absorption bands in azulene, *i*) weak, red-shifted band attributed to electronic transitions with low orbital overlap (***red band***), and *ii*) strong, blue-shifted band attributed to electronic transitions with high orbital overlap (***blue band)***.

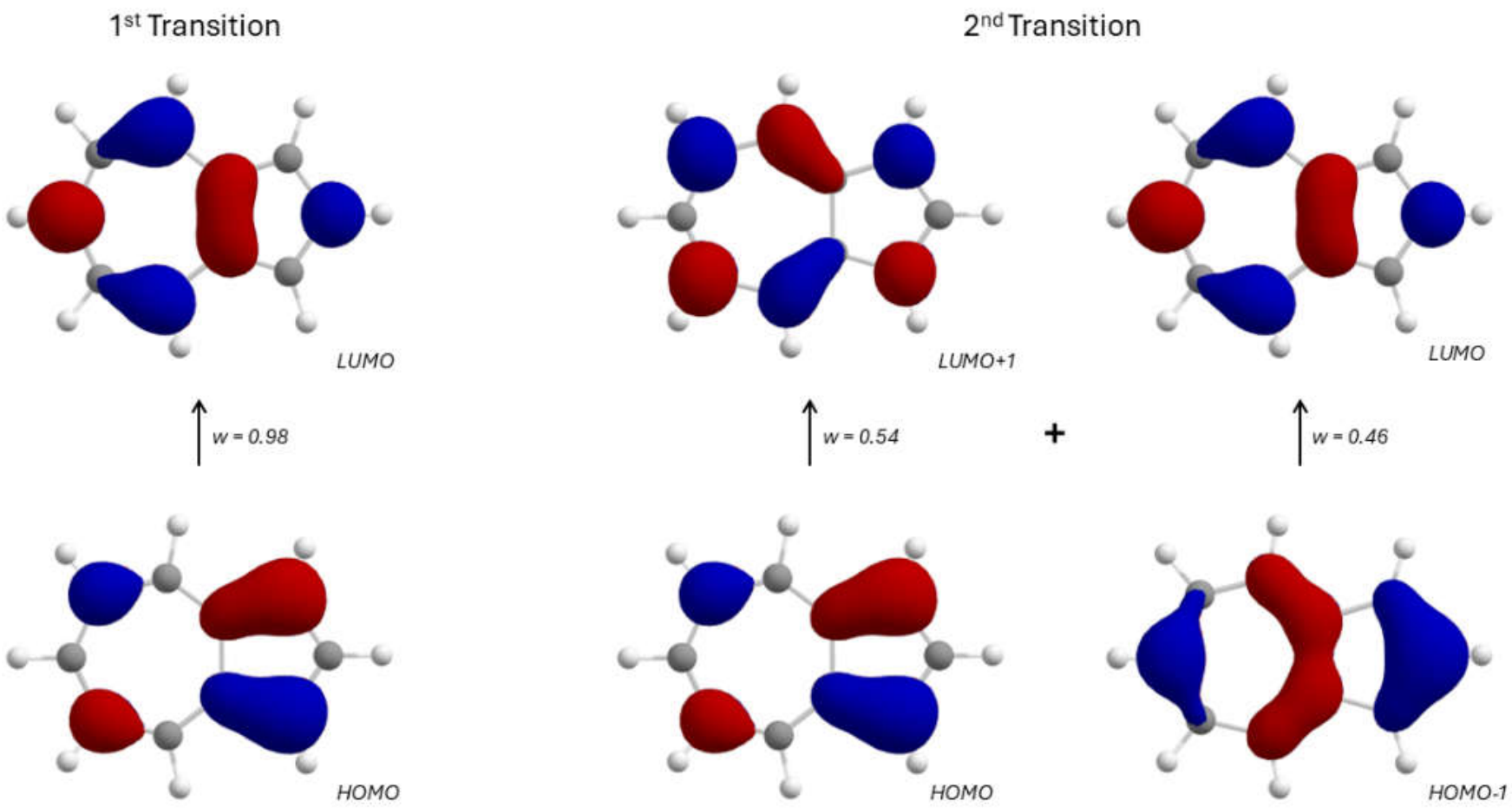

Figure S64. TD-DFT orbital assignment of azulene's 1st and 2nd electronic transitions, calculated at TD-B3LYP/def2-TZVP level of theory, rendered at iso = 0.03.

S8.4.2 2,2'-Biazulene and 6,6'-Biazulene

Similarly to azulene, UV-vis spectra of 2,2'- and 6,6'-biazulene also feature distinct absorption bands of two types; **blue bands -** strong, well-resolved bands in the blue/UV region of the spectrum, and a single **red band** - a weak band in the vis region ranging from 500-700 nm (Figure S65). However, the UV-vis absorption spectra of 2,2'- and 6,6'-biazulene were, to the best of our knowledge, not assigned computationally. Thus, we conducted the TD-DFT study and assigned the UV-vis absorption bands herein. Assigning all measured UV-vis bands required considering the first 7 TD-DFT transitions.

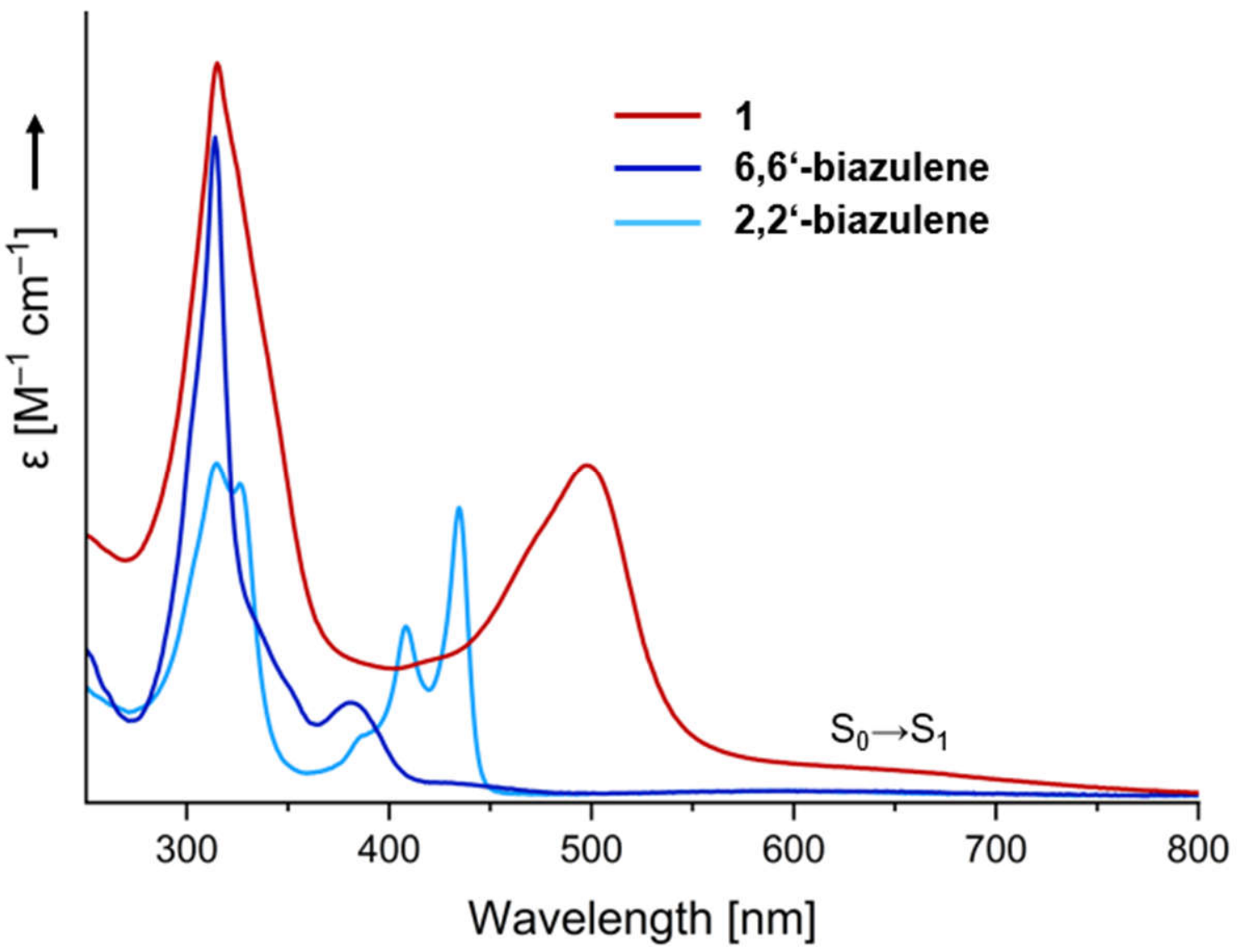

Figure S65. UV-Vis Absorption spectra of [6]CPA (**1**), 2,2'-biazulene and 6,6'-biazulene, recorded in chloroform.[37]

The **first electronic transition** of **both 2,2'- and 6,6'- azulene**, assigned as a HOMO→LUMO transition, lacks any CT character. Instead, its red-shifted position in the visible spectrum is dictated by the extensive π-conjugation of the biazulenes and, importantly, low spatial overlap of HOMO and LUMO (stemming from non-alternant topology of azulene), thereby low Coulombic repulsion between the unpaired electrons in the S_1 state.

Even though the first electronic transition has no CT character, the low spatial overlap of HOMO and LUMO also results in low oscillator strength of the first transition in both biazulenes. Moreover, 2,2'- and 6,6'- biazulene are centrosymmetric in their (co)planar conformation. Therefore, because HOMO and LUMO share the same symmetry (towards *i*), the first transition is symmetrically forbidden by Laporte's rule, in planar conformation – the minimum energy geometry of 2,2'-biazulene.

The **second transition**, while not symmetrically forbidden in either biazulene, is spatially equivalent to the first transition. Therefore, the second transition has similar, red-shifted energy to the first transition. Moreover, the second transition suffers from the same low spatial overlap and, consequently, low intensity.

Thus, we can conclude that the **first and second electronic transition** of both 2,2'- and 6,6'- azulene share their character with the first electronic transition in azulene. Consequently, in the UV-vis absorption spectra, the first and second electronic transitions of biazulenes manifest as azulene-type **red bands** (Figure S65). Notably, the red bands in azulenes and biazulenes share the same position, showing that the **extent of π-conjugation does not significantly influence the position of azulene-type red bands as they are, instead, more strongly influenced by the low S_1 Coloumbic repulsion stemming from the non-alternant topology of azulene.**

Higher transitions, with significantly higher oscillator strength are, in turn, classified as azulene type **blue bands**. They are typical π–π* transitions with high spatial overlap. Thus, they have higher intensity, sharper/vibrationally resolved, and are influenced by the extent of π-conjugation. Because π-conjugation can be perturbed by conformation, and 2,2'- and 6,6'-biazulene do not share the same conformation, they must be discussed separately.

In 2,2' biazulene, with a (co)planar minimum energy geometry, we found that only 2nd, 3rd, and 7th transitions significantly contribute to the measured UV-Vis absorption spectra. These transitions are responsible for bands around ca. 550, 430, and 330 nm, thereby assigning all bands in the UV-vis spectra.

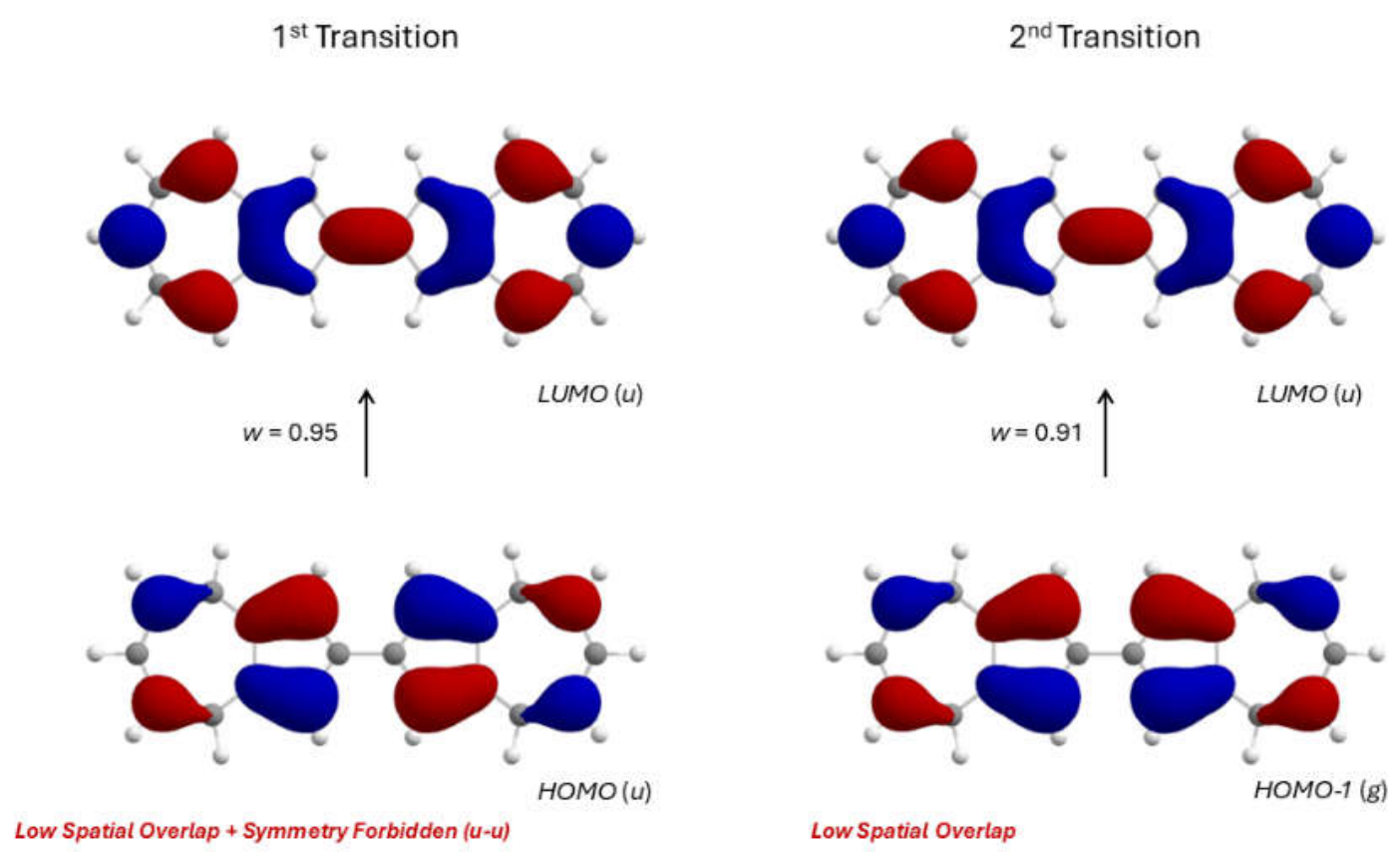

Figure S66. Orbital assignment of the 1st and 2nd electronic transitions of 2,2'-biazulene, calculated at TD-B3LYP/def2-TZVP level of theory, rendered at iso = 0.03.

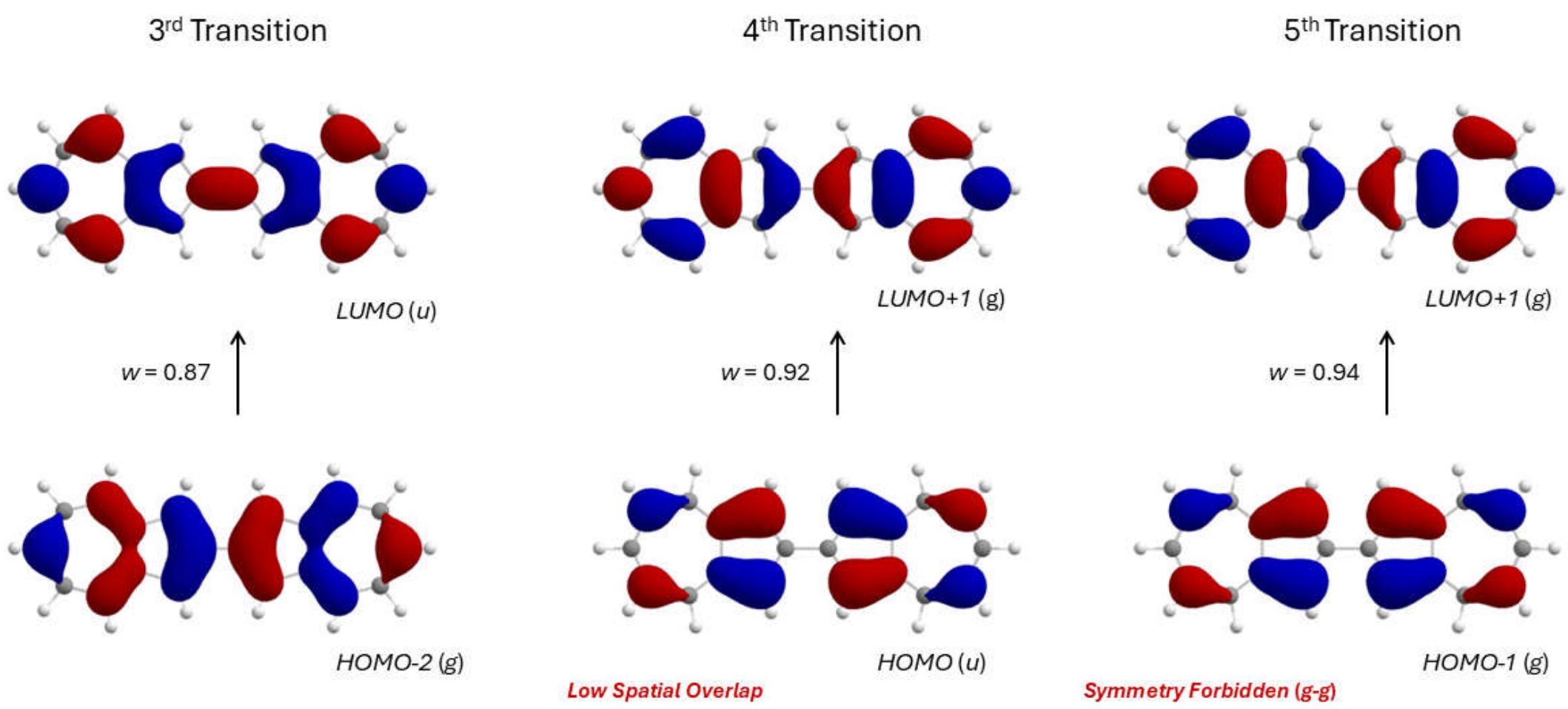

Figure S67. Orbital assignment of the 3rd-5th electronic transitions of 2,2'-biazulene, calculated at TD-B3LYP/def2-TZVP level of theory, rendered at iso = 0.03.

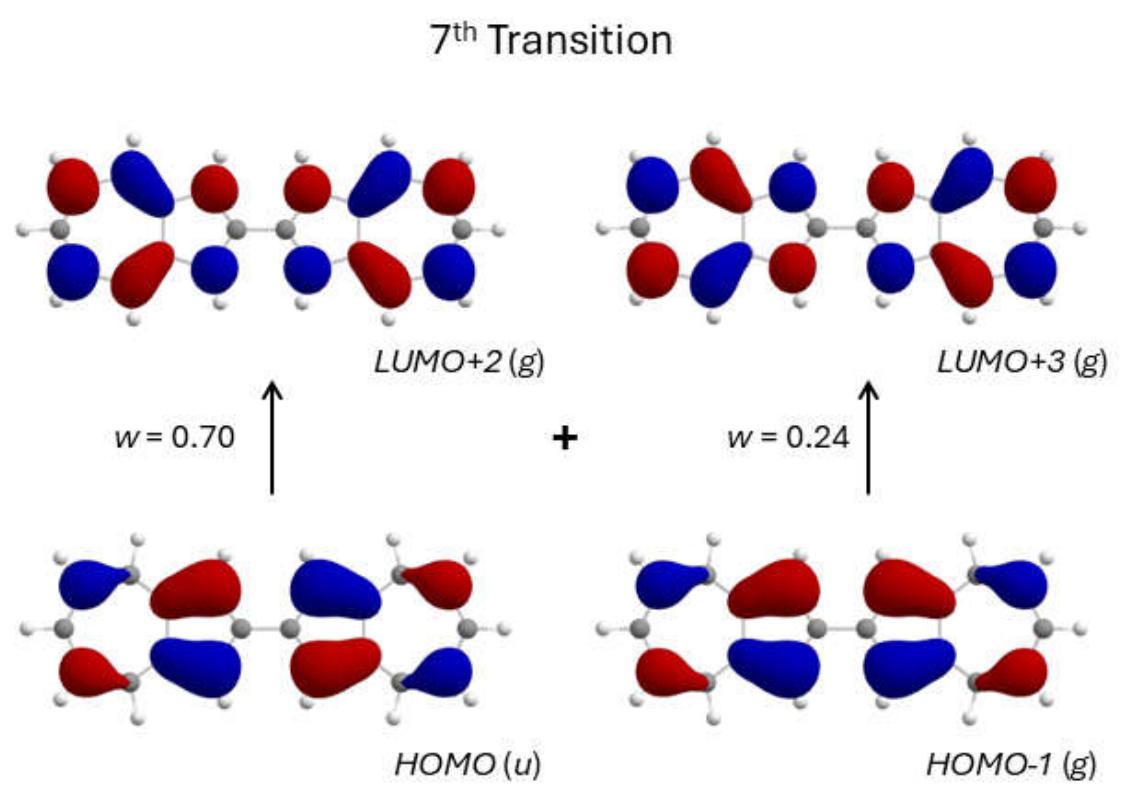

Figure S68. Orbital assignment of the 7th electronic transitions of 2,2'-biazulene, calculated at TD-B3LYP/def2-TZVP level of theory, rendered at iso = 0.03.

In 6,6'-biazulene, which has a single minimum energy geometry in a skewed conformation, with a dihedral angle of 52° (between two azulene subunits), we found that the 1st, 2nd, 4th, 5th and 7th transition significantly contribute to the measured UV-Vis absorption spectra at the minimum energy geometry. These transitions are responsible for bands around ca. 570, 560, 400, 380 and 330 nm, thereby assigning all bands in the UV-vis spectra. Moreover, owing to the relative conformational flexibility of 6,6'-biazulene ($\Delta E_{0°}$ = 7.6 kcal/mol, $\Delta E_{90°}$ = 1.4 kcal/mol), non-equilibrium rotamers may contribute to the UV-Vis absorption spectra, particularly through the 3rd transition at ca. 400 nm.

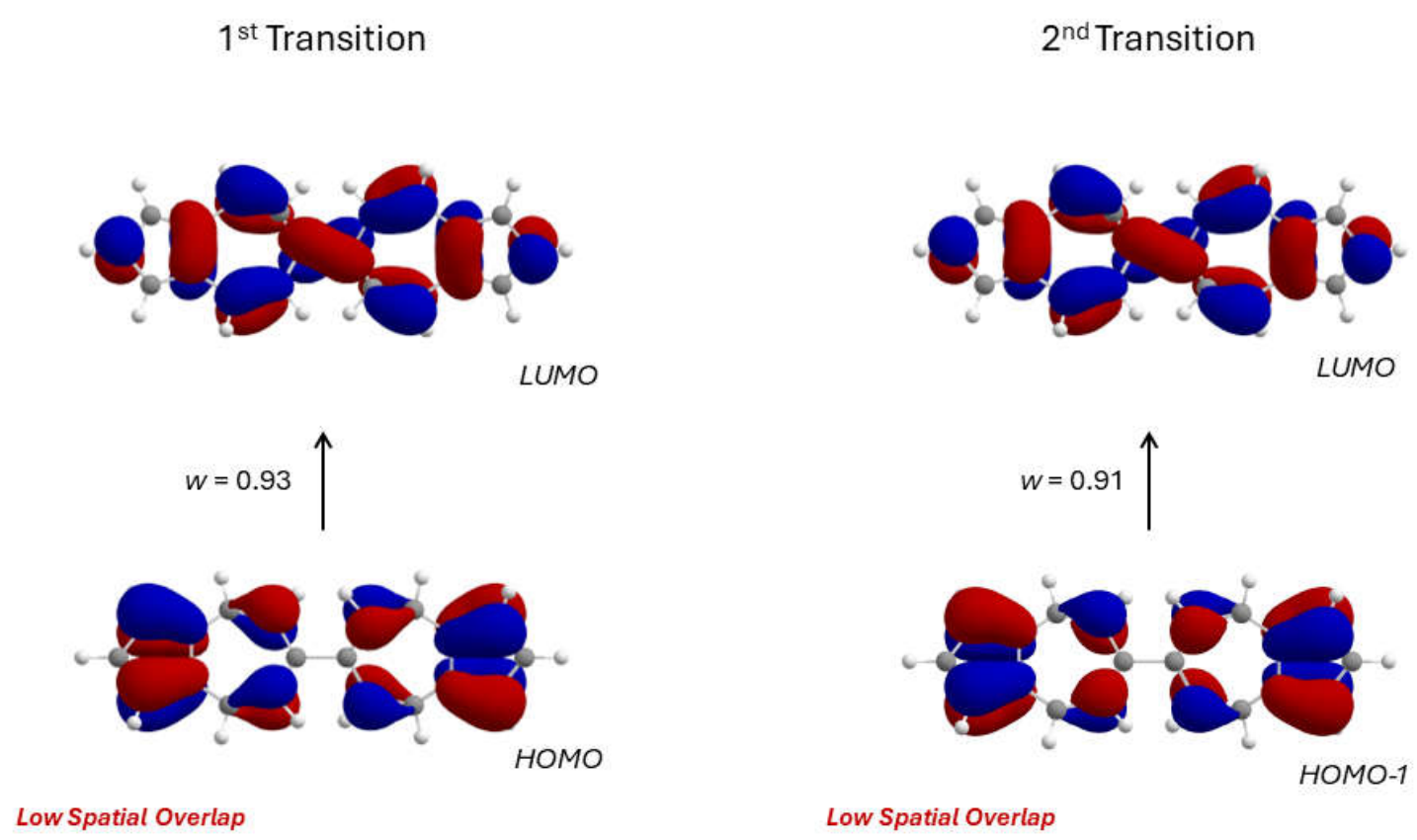

Figure S69. Orbital assignment of the 1st and 2nd electronic transitions of 6,6'-biazulene, calculated at TD-B3LYP/def2-TZVP level of theory, rendered at iso = 0.03.

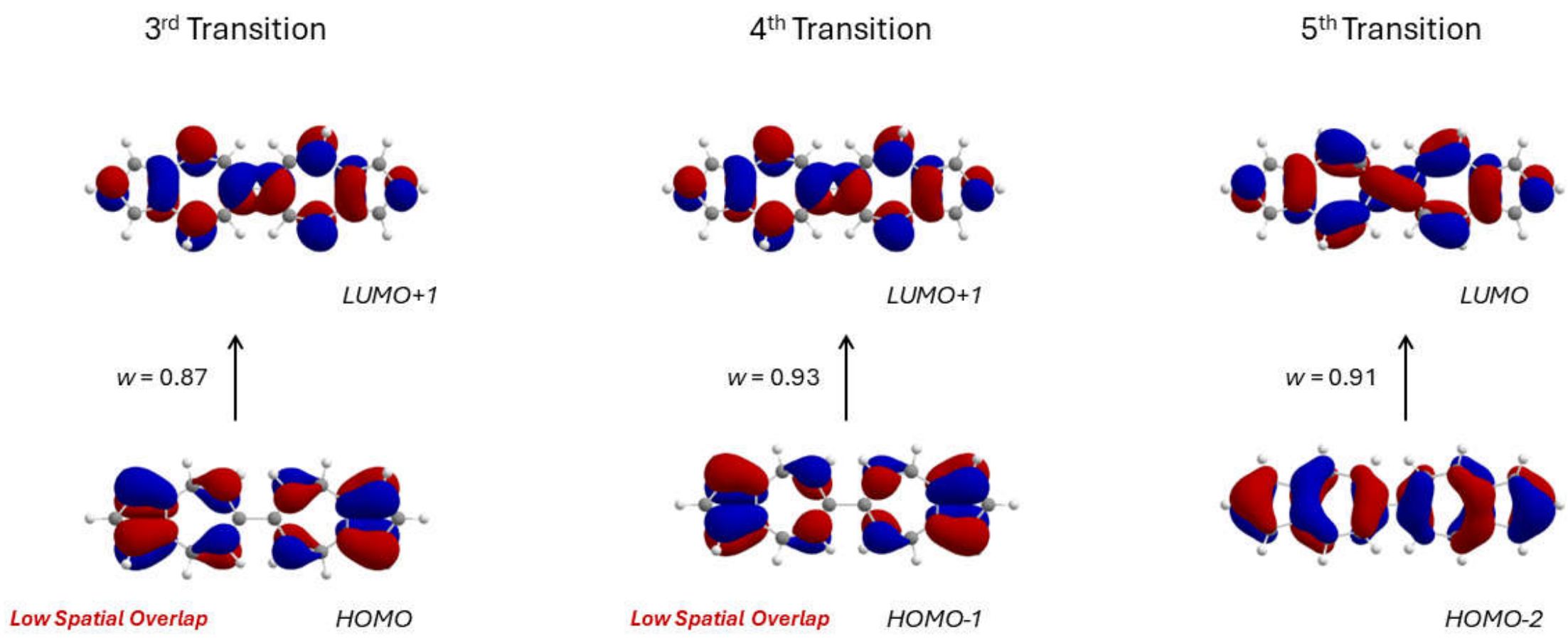

Figure S70. Orbital assignment of the 3rd-5th electronic transitions of 6,6'-biazulene, calculated at TD-B3LYP/def2-TZVP level of theory, rendered at iso = 0.03.

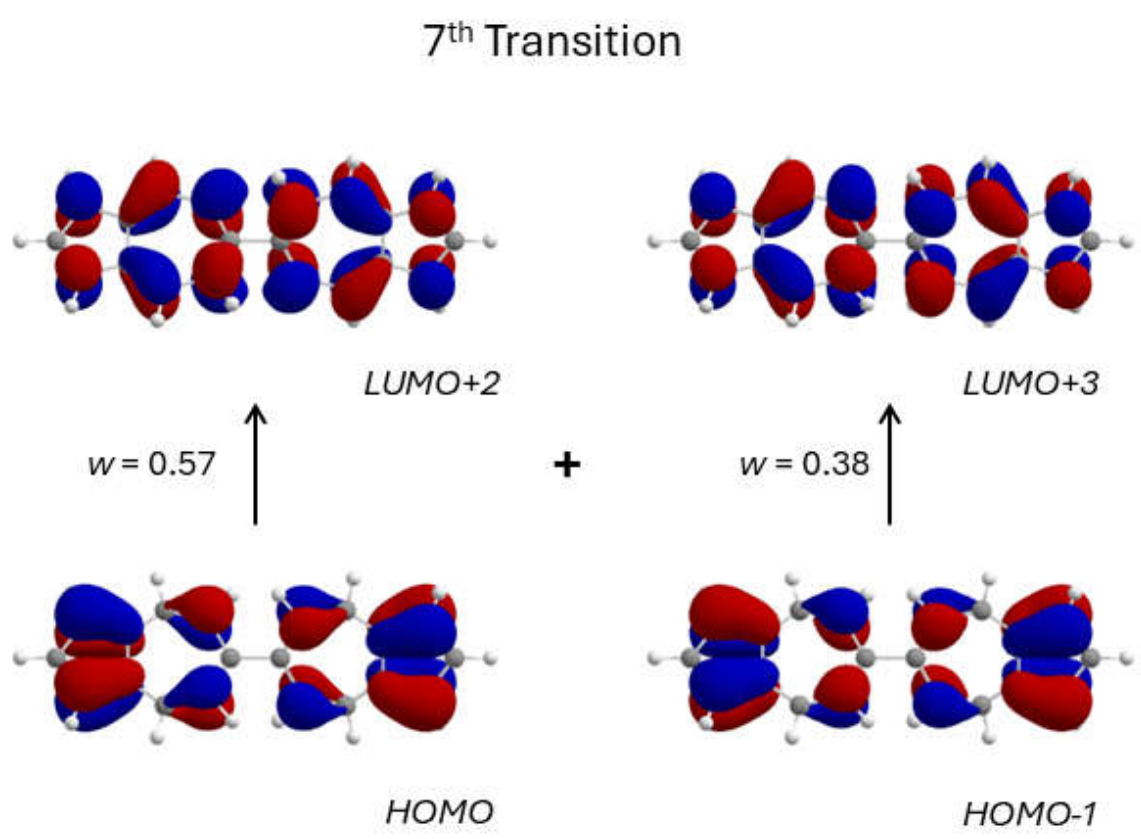

Figure S71. Orbital assignment of the 7th electronic transition of 6,6'-biazulene, calculated at TD-B3LYP/def2-TZVP level of theory, rendered at iso = 0.03.

The UV-vis absorption spectra of 2,2'- and 6,6'-biazulene are determined by the dihedral angle between the two azulene subunits. This can be particularly well illustrated by calculating TD-DFT transitions along the NEB isomerization pathways (Figure S72 and S73), and plotting the energy of 3rd, 4th and 5th transitions (as wavelength) against the dihedral angle in the biazulenes. Doing so reveals that, at the same conformation, 2,2'- and 6,6'-biazulene share the same transition energies. Thus, differences in the measured absorption spectra can be attributed to crossing of the 3rd and 5th excited state in the skewed, 6,6'-biazulene.

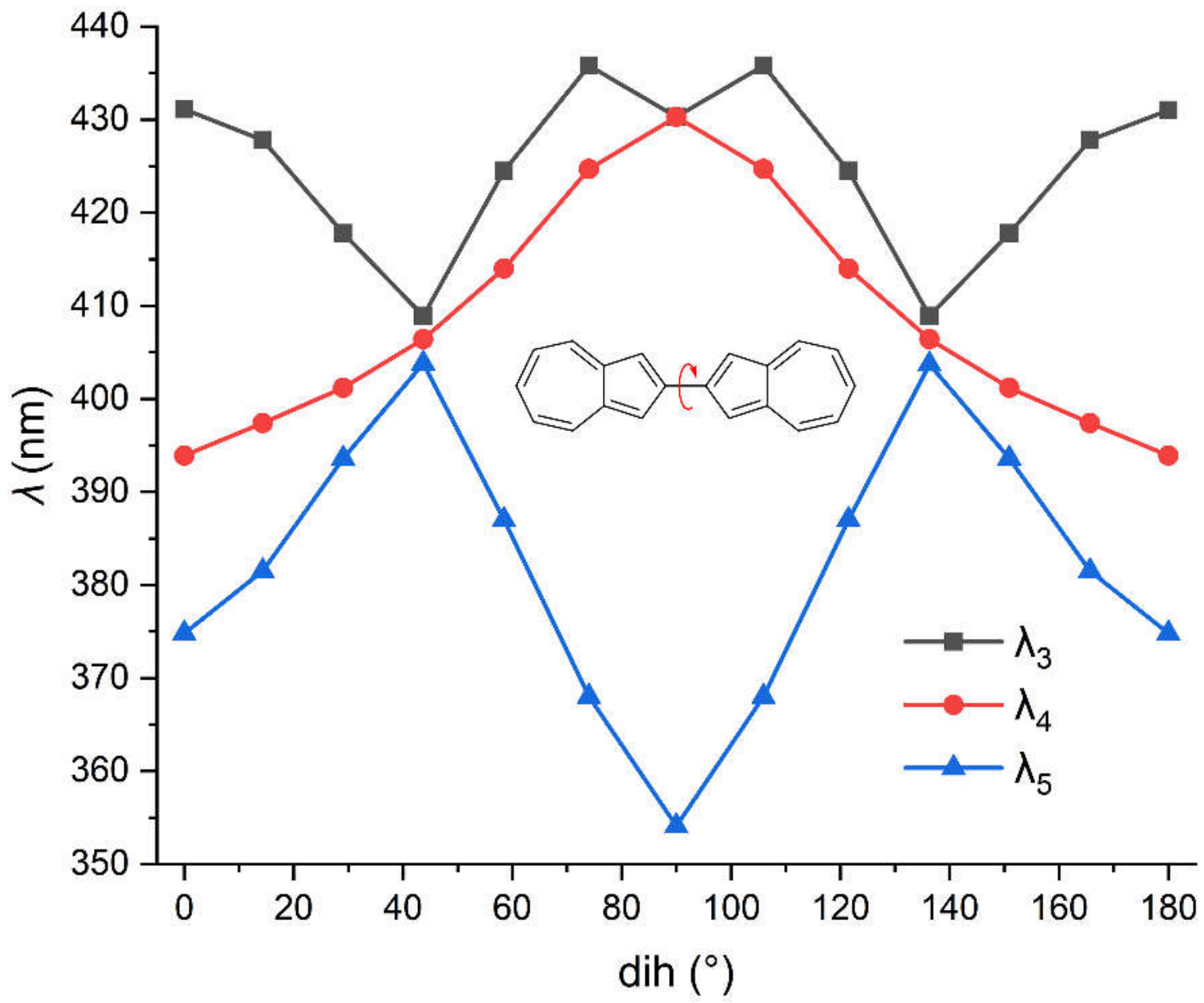

Figure S72. Calculated wavelengths of the 3rd, 4th and 5th electronic transitions (λ_3, λ_4 and λ_5, respectively) of each image of the NEB isomerization pathway of **2,2'-biazulene** (see Chapter S8.3.1), at TD-B3LYP/def2-TZVP level of theory, plotted against the dihedral angle between its two azulene subunits.

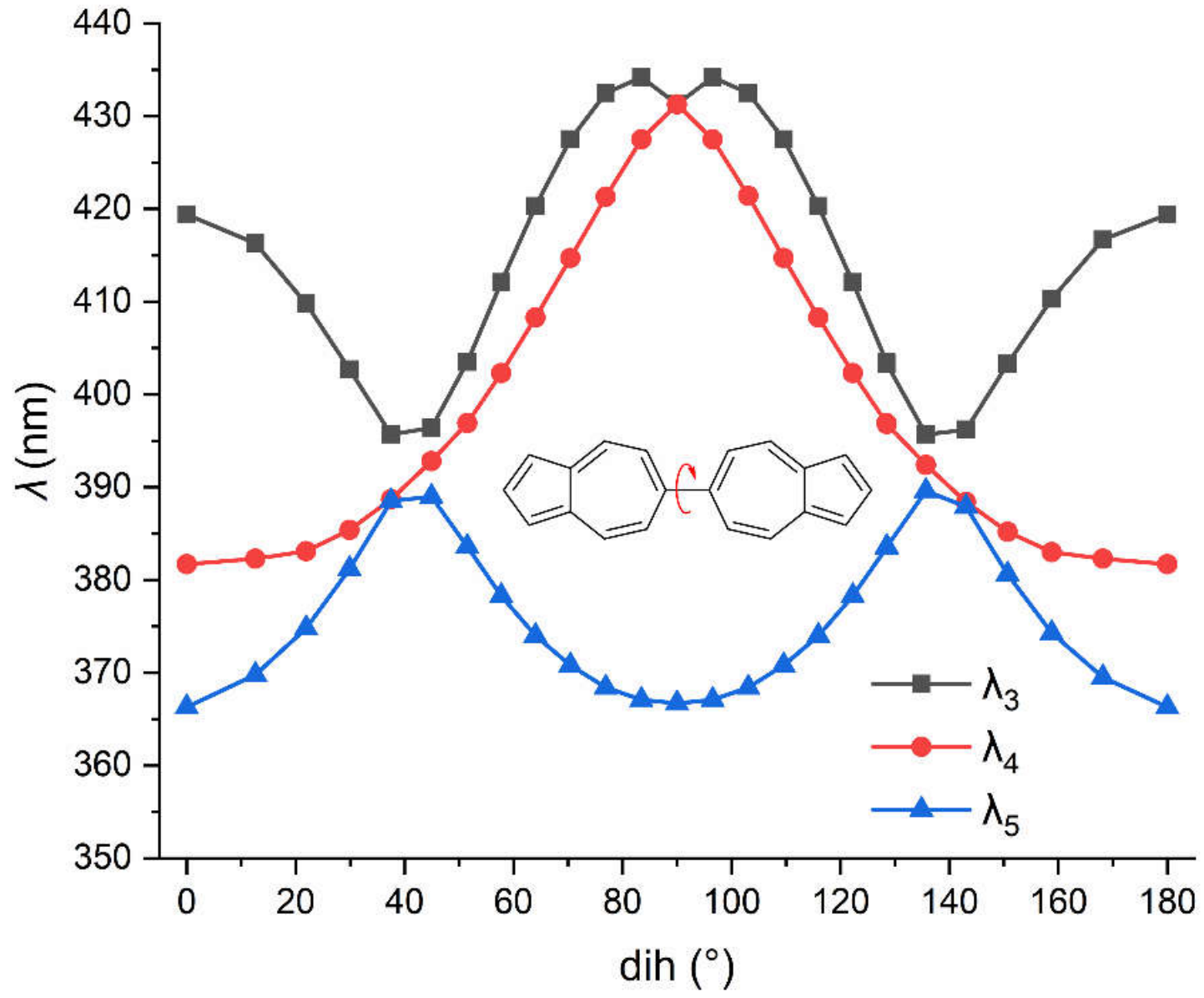

Figure S73. Calculated wavelengths of the 3rd, 4th and 5th electronic transitions (λ_3, λ_4 and λ_5, respectively) of each image of the NEB isomerization pathways of **6,6'-biazulene** (composite image of two pathways, see Chapter S8.3.1), at TD-B3LYP/def2-TZVP level of theory, plotted against the dihedral angle between its two azulene subunits.

S8.4.3 [6]CPA

By extension, we can apply the same red-band/blue-band framework to [6]CPA, as it is yet another oligomer of azulene. We note that for [6]CPA, we applied a CAM-B3LYP functional, which more accurately describes long-distance interelectron interactions.[38]

UV-vis absorption spectra of [6]CPA feature a now characteristic, broad azulene-type **red band**, centered at roughly 600 nm. Notably, unlike e.g. [6]CPP, [6]CPA is not centrosymmetric, thereby it is not limited by Laporte's rule. Thus, the low intensity of this band is likely caused by a low spatial overlap of the associated, first six electronic transitions of [6]CPA.

The first intense UV-vis absorption band at 500 nm is the first π–π* transition not limited by low orbital overlap, and thus, is classified as a now characteristic azulene-type **blue band**, analogous to the 2nd transition in azulene, or to the 3rd transition in 2,2'-biazulene. Its red-shift compared to azulene and biazulene is caused by extended π-conjugation. Compared to azulene and 2,2'-biazulene, the red-shift is likely proportionally lower due to poorer conjugation of azulene subunits in [6]CPA, caused by skewed conformation (strain) of the biazulene subunits. This spectral region is satisfyingly described by the 8th and 9th transition.

Similarly to 2,2'-biazulene, many higher transitions are of low consequence, as they have much lower oscillator strength and, thus, do not result in any discernible spectral feature in the UV-vis absorption spectra. The last absorption measured band, with a maximum of ca. 320 nm, is associated with the 33rd and 34th transition.

S8.5 Atomic Coordinates of Calculated Structures

1 (B3LYP/def2-TZVP)

C	4.85650000	3.92500000	–1.23940000
C	4.56890000	4.63980000	–0.06060000
C	5.30880000	4.05930000	1.01030000
C	5.92270000	2.90280000	0.55100000
C	5.61070000	2.79550000	–0.89820000
C	5.80630000	1.68840000	–1.70610000
C	6.12770000	0.38550000	–1.33560000
C	6.49920000	–0.13750000	–0.09250000
C	6.87820000	0.63360000	1.03250000

C	6.62870000	1.96160000	1.31090000
H	4.40100000	4.09430000	–2.20250000
H	5.31850000	4.41150000	2.03050000
H	6.91970000	2.29100000	2.30430000
H	7.34730000	0.07940000	1.83580000
H	5.94400000	–0.34460000	–2.11160000
H	5.50890000	1.81180000	–2.74320000
C	–1.70260000	6.17000000	0.08760000
C	–1.10970000	5.91040000	1.32850000
C	0.30980000	6.82200000	–1.29910000
C	0.23050000	5.90330000	1.70280000
C	1.35670000	6.00850000	0.90370000
C	3.41960000	5.50750000	0.07580000
C	1.39370000	6.36990000	–0.53770000
C	2.67630000	6.09030000	–0.99020000
C	–1.04110000	6.73180000	–1.02920000
C	2.63930000	5.56660000	1.24750000
H	2.91300000	5.15170000	2.20490000
H	–1.77890000	5.54650000	2.09590000
H	–1.68870000	7.05780000	–1.83360000
H	0.56160000	7.19670000	–2.28720000
H	0.41880000	5.62270000	2.73490000
H	3.02440000	6.21570000	–2.00420000
H	–3.30830000	–4.91950000	–2.09770000
C	–2.75580000	–5.43370000	–1.32360000
C	–3.39140000	–5.50810000	–0.07910000
H	–1.21080000	–5.57930000	–2.73420000
C	–1.46130000	–5.78290000	–1.69740000
C	–2.89750000	–6.21260000	1.04440000
C	–0.39760000	–6.15810000	–0.89430000
H	1.32410000	–5.74830000	–2.20510000
C	0.95530000	–6.06320000	–1.24140000

H	–3.60660000	–6.35210000	1.85080000
C	–0.45230000	–6.49250000	0.55290000
C	–1.61530000	–6.64380000	1.31760000
H	3.84740000	–5.64350000	–2.02940000
C	1.72590000	–6.18420000	–0.06850000
C	3.93180000	–5.29430000	–1.01160000
C	0.85920000	–6.54070000	1.00470000
H	5.97090000	–4.05230000	–2.31070000
H	–1.46660000	–7.05930000	2.31020000
C	3.06110000	–5.64370000	0.06070000
C	5.78670000	–3.65130000	–1.31810000
C	4.84200000	–4.35020000	–0.55680000
H	1.16490000	–6.73420000	2.02150000
H	7.02270000	–2.06100000	–1.84420000
C	6.41160000	–2.45260000	–1.04070000
C	3.53040000	–5.02370000	1.23520000
C	4.56880000	–4.15030000	0.89050000
C	6.27540000	–1.60340000	0.08300000
H	3.04580000	–5.05610000	2.19850000
C	5.07060000	–3.14180000	1.69560000
C	5.76090000	–1.99110000	1.32520000
H	4.74590000	–3.17000000	2.73150000
H	5.79780000	–1.23730000	2.09950000
H	–3.69850000	6.25310000	1.82960000
C	–3.94390000	5.57040000	1.02570000
C	–3.07980000	5.62130000	–0.09330000
H	–5.41840000	4.79550000	2.28780000
C	–4.98040000	4.70070000	1.29820000
C	–3.33490000	5.02940000	–1.33500000
C	–5.44950000	3.62330000	0.53730000
H	–6.49810000	2.37150000	2.00880000
C	–6.17060000	2.52790000	0.99230000

H	–2.60190000	5.22970000	–2.10440000
C	–5.18210000	3.39390000	–0.90680000
C	–4.30220000	4.10100000	–1.70880000
H	–5.99620000	–0.09830000	2.20170000
C	–6.30460000	1.59400000	–0.07520000
C	–6.21020000	–0.54000000	1.24100000
C	–5.79830000	2.18460000	–1.24960000
H	–5.19640000	–2.50570000	2.74460000
H	–4.24930000	3.77220000	–2.74240000
C	–6.51410000	0.16950000	0.06280000
C	–5.32920000	–2.80450000	1.70910000
C	–5.96370000	–1.87580000	0.90170000
H	–5.71250000	1.69890000	–2.20910000
H	–4.01590000	–4.36280000	2.11440000
C	–4.65790000	–3.96690000	1.33970000
C	–6.64360000	–0.76510000	–1.00510000
C	–6.27180000	–2.02050000	–0.54510000
C	–4.56800000	–4.60570000	0.09820000
H	–6.90370000	–0.52310000	–2.02430000
C	–6.12490000	–3.18880000	–1.30300000
C	–5.37980000	–4.31620000	–1.02450000
H	–6.56640000	–3.15690000	–2.29500000
H	–5.33460000	–5.04230000	–1.82650000

2,2’-Biazulene (B3LYP/def2-TZVP)

C	3.98920000	–1.59030000	0.00000000
H	3.76050000	–2.65210000	0.00000000
C	5.34010000	–1.26120000	0.00000000
H	6.02740000	–2.09950000	0.00000000
C	1.54880000	–1.14600000	0.00000000
H	1.20990000	–2.17040000	0.00000000
C	2.88560000	–0.74730000	0.00000000
C	5.93070000	0.00000000	0.00000000

C	0.72260000	0.00000000	0.00000000
H	7.01570000	0.00000000	0.00000000
C	2.88560000	0.74730000	0.00000000
C	5.34010000	1.26120000	0.00000000
C	1.54880000	1.14600000	0.00000000
C	3.98920000	1.59030000	0.00000000
H	6.02740000	2.09950000	0.00000000
H	1.20990000	2.17040000	0.00000000
H	3.76050000	2.65210000	0.00000000
C	–3.98920000	–1.59030000	–0.00010000
H	–3.76050000	–2.65210000	–0.00010000
C	–5.34010000	–1.26120000	0.00000000
H	–6.02740000	–2.09950000	–0.00010000
C	–1.54880000	–1.14600000	0.00000000
H	–1.20990000	–2.17040000	–0.00010000
C	–2.88560000	–0.74730000	0.00000000
C	–5.93070000	0.00000000	0.00000000
C	–0.72260000	0.00000000	0.00000000
H	–7.01570000	0.00000000	0.00000000
C	–2.88560000	0.74730000	0.00000000
C	–5.34010000	1.26120000	0.00000000
C	–1.54880000	1.14600000	0.00000000
C	–3.98920000	1.59030000	0.00000000
H	–6.02740000	2.09950000	0.00000000
H	–1.20990000	2.17040000	0.00000000
H	–3.76050000	2.65210000	0.00000000

6,6’-Biazulene (B3LYP/def2-TZVP)

C	–0.74880000	0.00000000	–0.00010000
C	–1.37100000	–1.12690000	0.55120000
C	–2.72280000	1.41500000	–0.69770000
C	–2.72290000	–1.41480000	0.69770000
C	–3.83000000	–0.66840000	0.33000000

C	–5.17300000	–1.02740000	0.50890000
C	–3.82990000	0.66870000	–0.33000000
C	–5.17290000	1.02780000	–0.50890000
C	–1.37090000	1.12700000	–0.55130000
C	–5.97820000	0.00020000	0.00000000
H	–7.05890000	0.00030000	0.00000000
H	–0.69690000	1.87780000	–0.94500000
H	–0.69700000	–1.87770000	0.94490000
H	–2.94430000	–2.36440000	1.17670000
H	–2.94420000	2.36460000	–1.17670000
H	–5.51660000	1.94490000	–0.96280000
H	–5.51670000	–1.94450000	0.96280000
C	0.74880000	–0.00010000	–0.00010000
C	1.37090000	–1.12700000	–0.55130000
C	2.72290000	1.41470000	0.69760000
C	2.72280000	–1.41500000	–0.69780000
C	3.82990000	–0.66870000	–0.33010000
C	5.17290000	–1.02790000	–0.50900000
C	3.83000000	0.66830000	0.32990000
C	5.17300000	1.02740000	0.50880000
C	1.37100000	1.12690000	0.55120000
C	5.97820000	–0.00030000	–0.00010000
H	7.05890000	–0.00030000	–0.00010000
H	0.69710000	1.87770000	0.94490000
H	0.69690000	–1.87780000	–0.94500000
H	2.94410000	–2.36460000	–1.17670000
H	2.94440000	2.36440000	1.17660000
H	5.51670000	1.94450000	0.96270000
H	5.51660000	–1.94500000	–0.96290000

S9 Crystallographic Data

Crystal structure of compound **S1**.

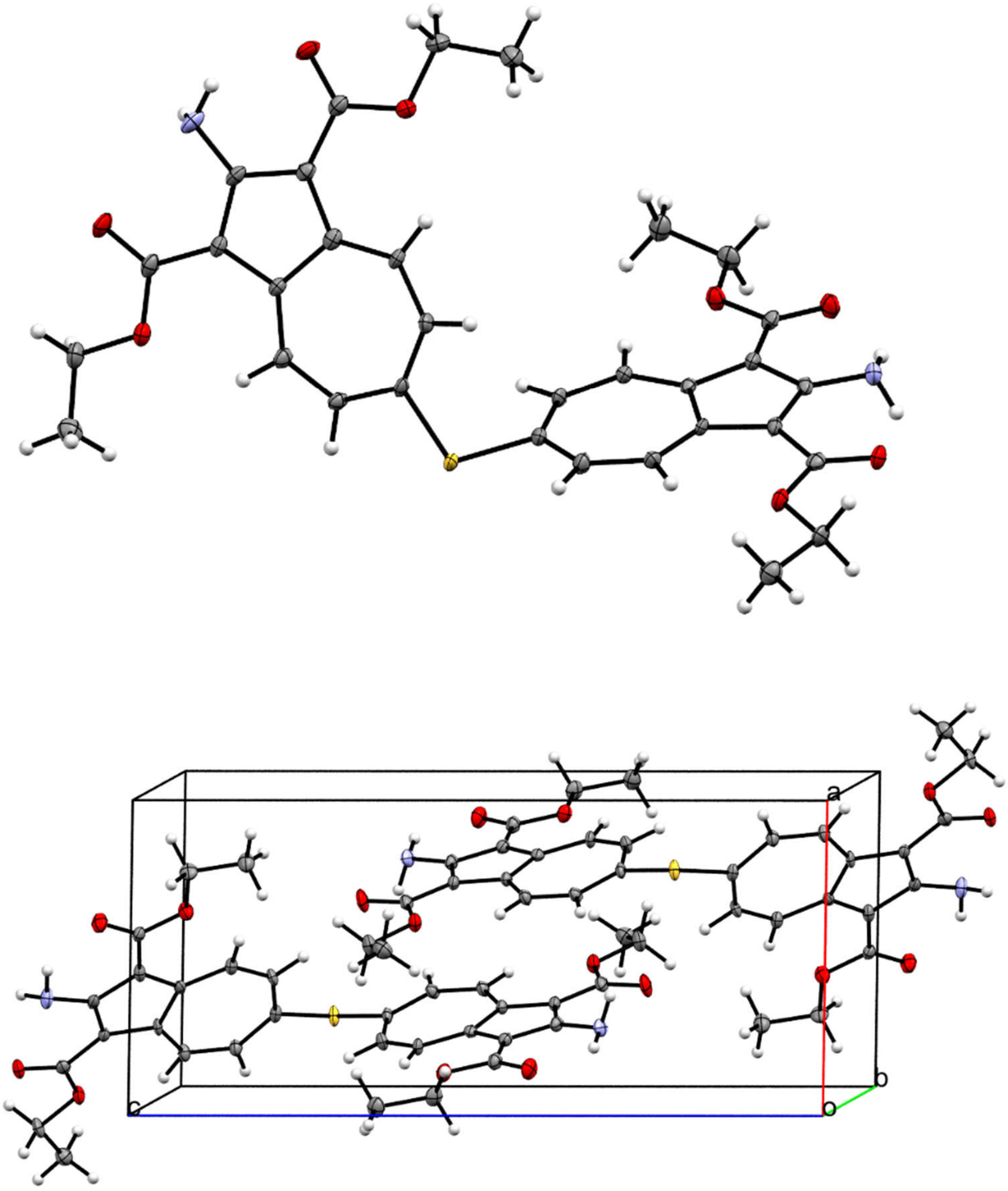

Figure S74. Crystal structure and packing of compound **S1**. The data was obtained by X-ray diffraction measurements at 100 K. The ellipsoids are displayed at 50% probability level. The crystals were obtained by slow evaporation of a cyclohexane/ethyl acetate solution at 25 °C. Color code: C, gray; O, red; N, blue; S, yellow; H, white.

Table S17. Single-Crystal X-ray data and structure refinement of **S1**.

CCDC number	2389465
Empirical formula	$C_{32}H_{32}N_2O_8S$
Formula weight	604.65
Temperature/K	100.00
Crystal system	triclinic
Space group	*P*–1
a/Å	8.1281(13)
b/Å	10.1673(15)
c/Å	17.808(3)
α/°	89.794(5)
β/°	89.400(6)
γ/°	75.712(6)
Volume/Å^3	1426.1(4)
Z	2
ρ_{calc} / g/cm^3	1.408
μ/mm^{-1}	0.171
F(000)	636.0
Crystal size/mm^3	0.659 × 0.367 × 0.121
Radiation	MoKα (λ = 0.71073)
2Θ range for data collection/°	4.134 to 59.368
Index ranges	–11 ≤ h ≤ 10, –14 ≤ k ≤ 14, –24 ≤ l ≤ 24
Reflections collected	22438
Independent reflections	7622 [R_{int} = 0.0583, R_{sigma} = 0.0772]
Data/restraints/parameters	7622/0/394
Goodness-of-fit on F^2	1.103
Final R indexes [$I \geq 2\sigma(I)$]	R_1 = 0.1359, wR_2 = 0.4087
Final R indexes [all data]	R_1 = 0.1569, wR_2 = 0.4202
Largest diff. peak/hole / e Å^{-3}	2.09/–0.91

Crystal structure of compound **3a.**

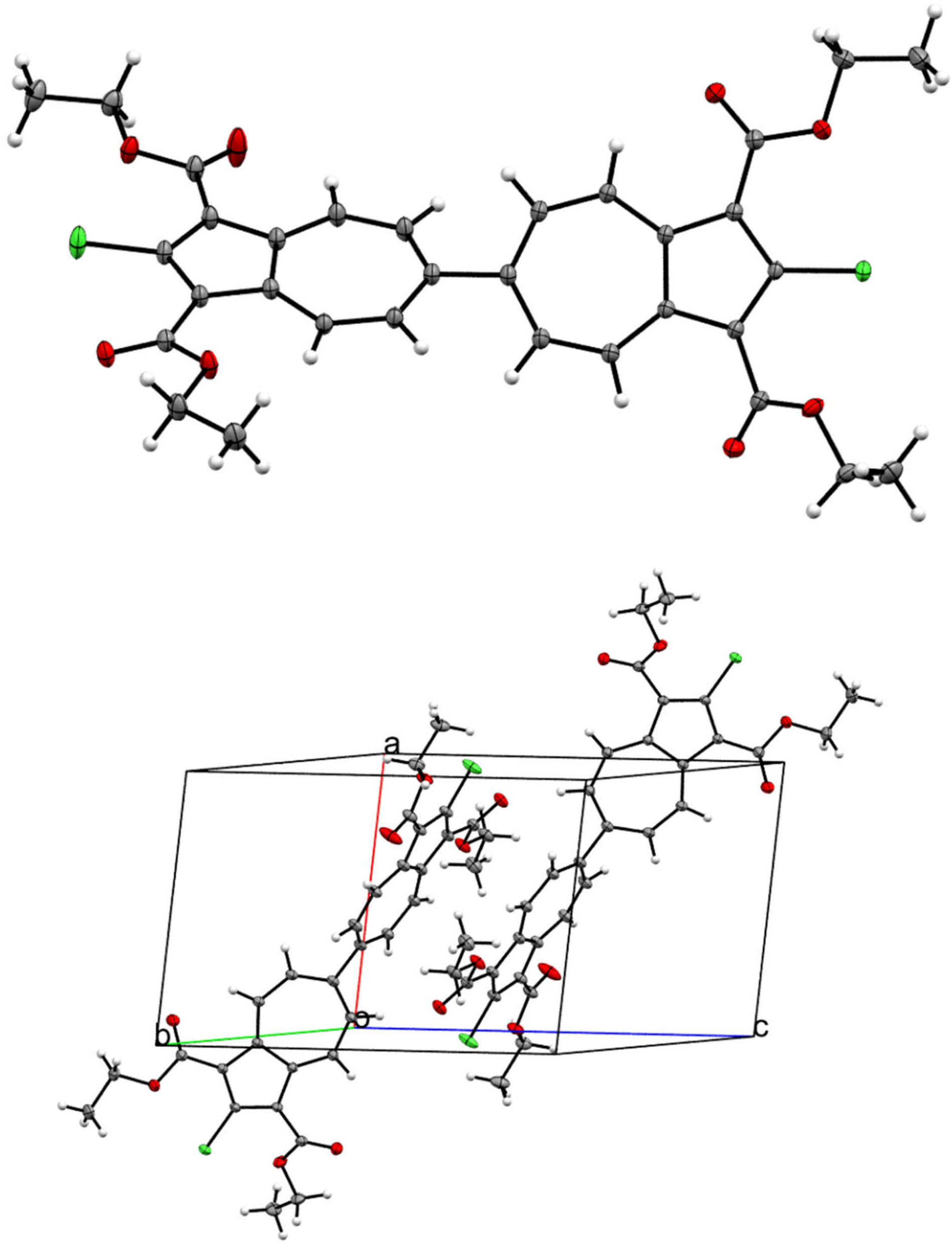

Figure S75. Crystal structure and packing of compound 3a. The data was obtained by X-ray diffraction measurements at 100 K. The ellipsoids are displayed at 50% probability level. The crystals were obtained by slow evaporation of a $CHCl_3$ solution at 25 °C. Color code: C, gray; O, red; Cl, green; H, white.

Table S18. Single-Crystal X-ray data and structure refinement of 3a.

CCDC number	2263372
Empirical formula	$C_{32}H_{28}Cl_2O_8$
Formula weight	611.44
Temperature/K	100.00
Crystal system	triclinic
Space group	*P*–1
a/Å	10.1275(5)
b/Å	10.7697(6)
c/Å	15.1734(7)
α/°	96.577(2)
β/°	99.227(2)
γ/°	117.812(2)
Volume/Å^3	1410.45(13)
Z	2
ρ_{calc} / g/cm^3	1.440
μ/mm^{-1}	0.284
F(000)	636.0
Crystal size/mm^3	0.266 × 0.169 × 0.163
Radiation	MoKα (λ = 0.71073)
2Θ range for data collection/°	4.38 to 73.042
Index ranges	–16 ≤ h ≤ 16, –17 ≤ k ≤ 18, –25 ≤ l ≤ 25
Reflections collected	133028
Independent reflections	13773 [R_{int} = 0.0664, R_{sigma} = 0.0382]
Data/restraints/parameters	13773/0/383
Goodness-of-fit on F^2	1.029
Final R indexes [$I \geq 2\sigma(I)$]	R_1 = 0.0441, wR_2 = 0.1010
Final R indexes [all data]	R_1 = 0.0683, wR_2 = 0.1139
Largest diff. peak/hole / e Å^{-3}	0.54/–0.47

Crystal structure of compound **1.**

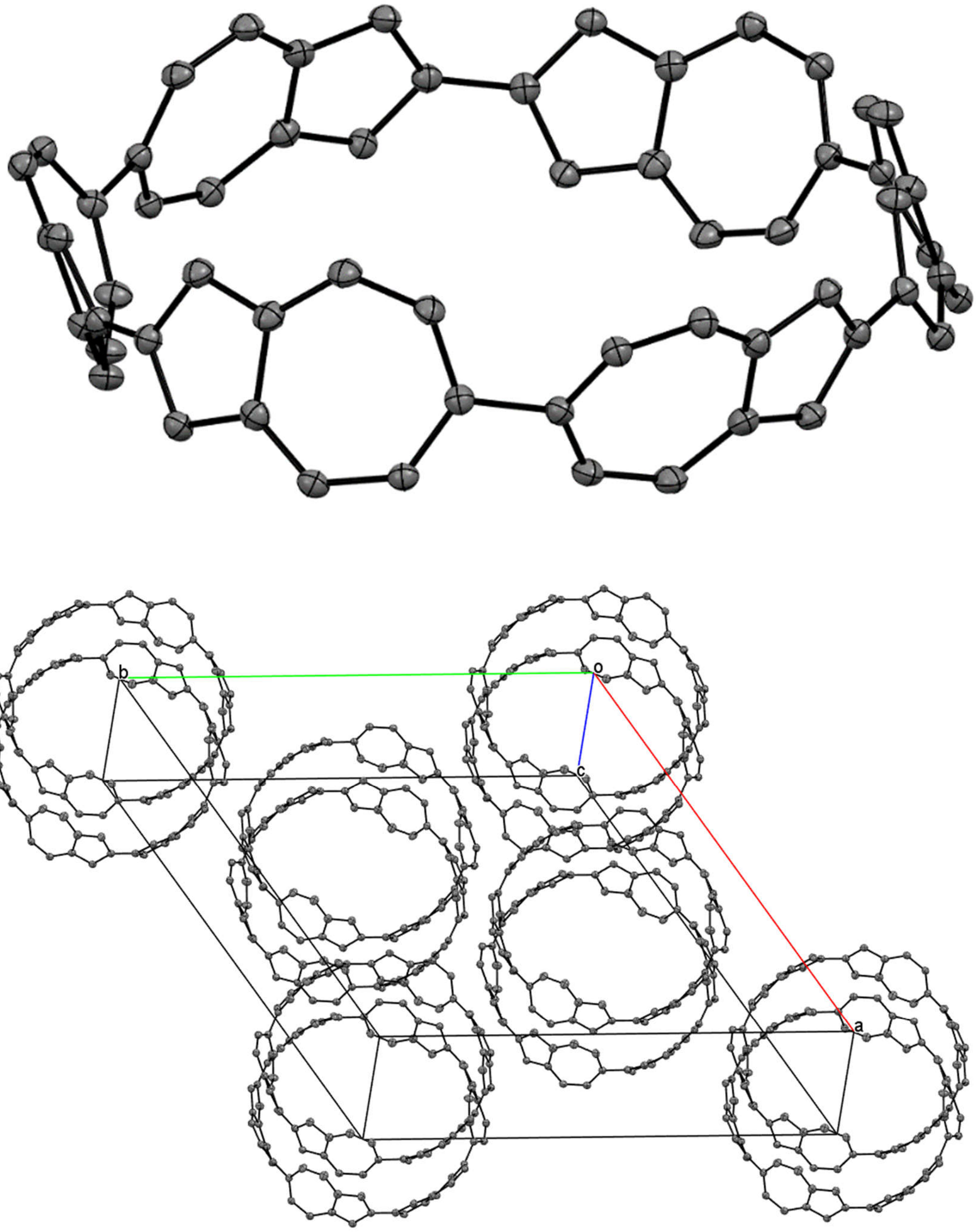

Figure S76. Crystal structure and packing of compound **1**. The data was obtained by X-ray diffraction measurements at 100 K. The ellipsoids are displayed at 50% probability level. The crystals were obtained by slow diffusion of cyclohexane into a CH_2Cl_2 solution of 1 at 25 °C. Color code: C, gray; H, white.

Table S19. Single-Crystal X-ray data and structure refinement of **1**.

CCDC number	2530569
Empirical formula	$C_{60}H_{36}$
Formula weight	756.89
Temperature/K	100.00
Crystal system	trigonal
Space group	*R*–3
a/Å	27.4226(6)
b/Å	27.4226(6)
c/Å	12.2978(4)
α/°	90
β/°	90
γ/°	120
Volume/Å^3	8008.9(4)
Z	6
ρ_{calc} / g/cm^3	0.942
μ/mm^{-1}	0.406
F(000)	2376.0
Crystal size/mm^3	0.05 × 0.02 × 0.02
Radiation	CuKα (λ = 1.54178)
2Θ range for data collection/°	46.446 to 144.34
Index ranges	$-33 \le h \le 33$, $-33 \le k \le 33$, $-13 \le l \le 15$
Reflections collected	49046
Independent reflections	3509 [R_{int} = 0.0777, R_{sigma} = 0.0339]
Data/restraints/parameters	3509/0/181
Goodness-of-fit on F^2	1.046
Final R indexes [$I \ge 2\sigma$ (I)]	R_1 = 0.0462, wR_2 = 0.1296
Final R indexes [all data]	R_1 = 0.0535, wR_2 = 0.1358
Largest diff. peak/hole / e Å^{-3}	0.24/–0.26

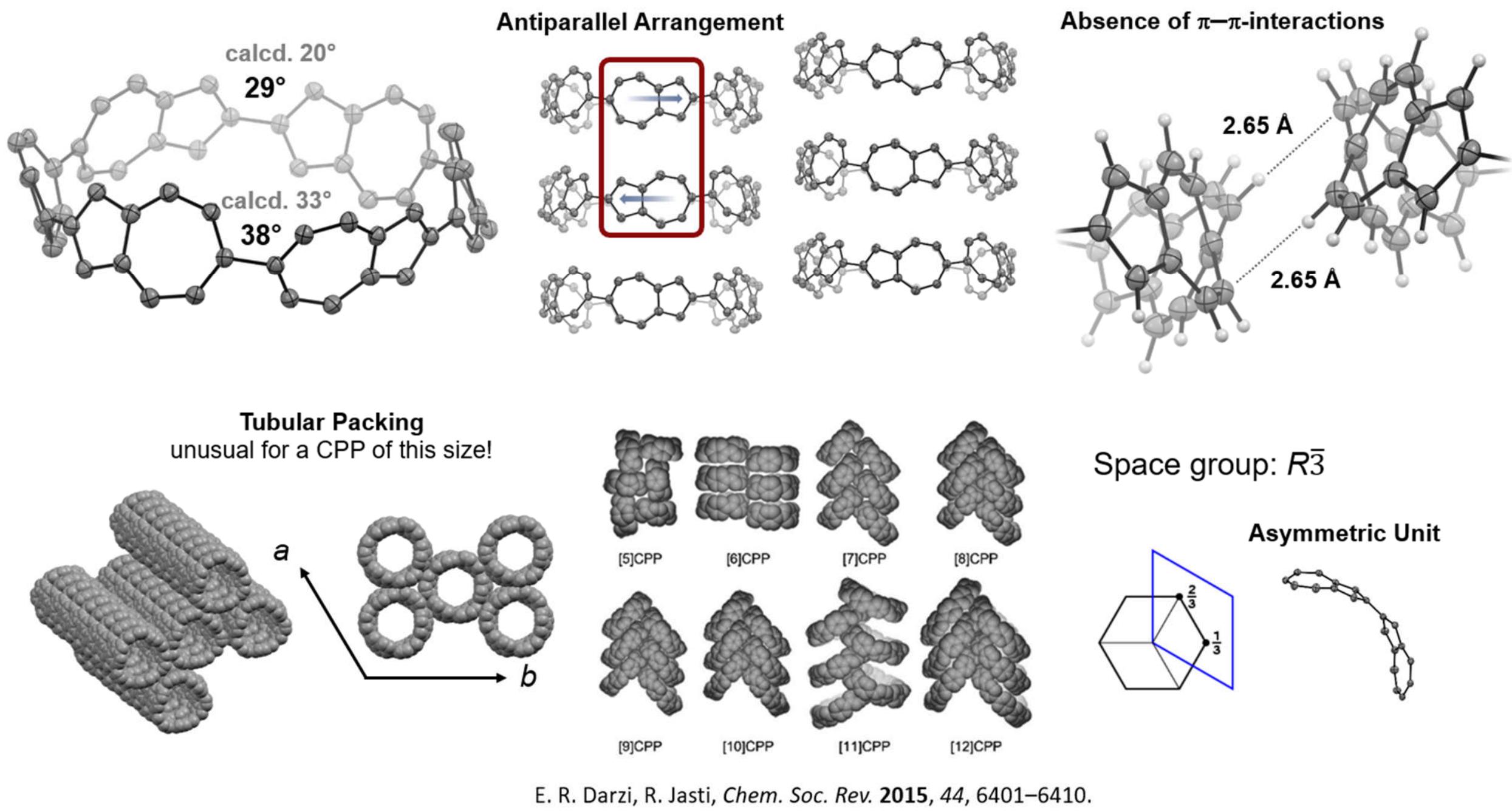

Figure S77. Crystal structure and packing analysis of compound **1**. Packing of [*n*]CPPs reprinted from Jasti and coworkers.[39]

Notes on the crystal structure of **1**

The crystal structure of **1** exhibits some unexpected features, the details of which are shown in Figure S77. The $R\bar{3}$ space group that **1** crystallizes in is highly symmetric, belonging to a trigonal crystal system and a rhombohedral lattice system. The molecular structure of **1** is generally consistent with the calculated minimum energy geometry structure, exhibiting the same geometry as the conformer found by CREST conformer analysis at the GFN2-xTB level of theory. The main structural difference in the crystal structure is higher torsion along the 2,2'- and 6,6'-connectivity compared to the calculated structure (θ_{XRD} = 29° vs. θ_{calc} = 20° for the 2,2'-connectivity and θ_{XRD} = 38° vs. θ_{calc} = 33° for the 6,6'-connectivity). The tubular solid-state packing of **1** is also notable, especially compared to similar-sized [9]CPP, which favors herringbone-type packing. Indeed, of [5-12]CPP, only [6]CPP exhibits tubular packing in the solid state, with all other ring sizes showing offset herringbone packing or, in the case of [5]CPP, brick wall packing.[39] Within the tubes, the dipoles of **1**'s azulene units are oriented antiparallel to the neighboring macrocycle, with each macrocycle being rotated by 1/6 compared to its direct neighbor. There is a notable absence of π–π-interactions between the tubes, with only weak inter-tube C—H···C interactions being observed. Despite the ~30% cavity volume in the crystal lattice, in which residual electron density is observable, the crystals of **1** are stable in perfluorinated oil at ambient conditions, with the crystal quality remaining high for about 2 weeks after the initial measurement. In comparison, amorphous **1** decomposes within several days when stored under Argon at –20 °C.

S10 References

[1] Y. Tsuchido, R. Abe, T. Ide, K. Osakada, *Angew. Chem. Int. Ed.* **2020**, *59*, 22928–22932.

[2] G. M. Sheldrick, *Acta Crystallogr. Sect. C Struct. Chem.* **2015**, *71*, 3–8.

[3] O. V Dolomanov, L. J. Bourhis, R. J. Gildea, J. A. K. Howard, H. Puschmann, *J. Appl. Crystallogr.* **2009**, *42*, 339–341.

[4] T. Nozoe, S. Seto, S. Matsumura, *Bull. Chem. Soc. Jpn.* **1962**, *35*, 1990–1998.

[5] L. C. Murfin, M. Weber, S. J. Park, W. T. Kim, C. M. Lopez-Alled, C. L. McMullin, F. Pradaux-Caggiano, C. L. Lyall, G. Kociok-Köhn, J. Wenk, S. D. Bull, J. Yoon, H. M. Kim, T. D. James, S. E. Lewis, *J. Am. Chem. Soc.* **2019**, *141*, 19389–19396.

[6] K. Kurotobi, H. Tabata, M. Miyauchi, T. Murafuji, Y. Sugihara, *Synthesis* **2002**, *8*, 1013–1016.

[7] T. R. Maher, A. D. Spaeth, B. M. Neal, C. L. Berrie, W. H. Thompson, V. W. Day, M. V Barybin, *J. Am. Chem. Soc.* **2010**, *132*, 15924–15926.

[8] B. Hou, Z. Zhou, C. Yu, X. S. Xue, J. Zhang, X. Yang, J. Li, C. Ge, J. Wang, X. Gao, *ACS Macro Lett.* **2022**, *11*, 680–686.

[9] S. Hitosugi, D. Tanimoto, W. Nakanishi, H. Isobe, *Chem. Lett.* **2012**, *41*, 972–973.

[10] J. R. Long, R. S. Drago, *J. Chem. Educ.* **1982**, *59*, 1037.

[11] P. Thordarson, *Chem. Soc. Rev.* **2011**, *40*, 1305–1323.

[12] F. Neese, *WIREs Comput. Mol. Sci.* **2012**, *2*, 73–78.

[13] F. Neese, *WIREs Comput. Mol. Sci.* **2018**, *8*, e1327.

[14] F. Neese, F. Wennmohs, U. Becker, C. Riplinger, *J. Chem. Phys.* **2020**, *152*, 224108.

[15] M. J. Frisch, G. W. Trucks, H. B. Schlegel, G. E. Scuseria, M. A. Robb, J. R. Cheeseman, G. Scalmani, V. Barone, B. Mennucci, G. A. Petersson, H. Nakatsuji, M. Caricato, X. Li, H. P. Hratchian, A. F. Izmaylov, J. Bloino, G. Zheng, J. L. Sonnenberg, M. Hada, M. Ehara, K. Toyota, R. Fukuda, J. Hasegawa, M. Ishida, T. Nakajima, Y. Honda, O. Kitao, H. Nakai, T. Vreven, J. A. Montgomery, J. E. Peralta, F. Ogliaro, M. Bearpark, J. J. Heyd, E. Brothers, K. N. Kudin, V. N. Staroverov, R. Kobayashi, J. Normand, K. Raghavachari, A. Rendell, J. C. Burant, S. S. Iyengar, J. Tomasi, M. Cossi, N. Rega, J. M. Millam, M. Klene, J. E. Knox, J. B. Cross, V. Bakken, C. Adamo, J. Jaramillo, R. Gomperts, R. E. Stratmann, O. Yazyev, A. J. Austin, R. Cammi, C. Pomelli, J. W. Ochterski, R. L. Martin, K. Morokuma, V. G. Zakrzewski, G. A. Voth, P. Salvador, J. J. Dannenberg, S. Dapprich, A. D. Daniels, Ö. Farkas, J. B. Foresman, J. V Ortiz, J. Cioslowski, D. J. Fox, *Wallingford CT* **2009**, Gaussian 09, Revision E.01.

[16] P. Pracht, F. Bohle, S. Grimme, *Phys. Chem. Chem. Phys.* **2020**, *22*, 7169–7192.

[17] D. W. Szczepanik, *Comput. Theor. Chem.* **2016**, *1080*, 33–37.

[18] D. W. Szczepanik, M. Andrzejak, J. Dominikowska, B. Pawełek, T. M. Krygowski, H. Szatylowicz, M. Solà, *Phys. Chem. Chem. Phys.* **2017**, *19*, 28970–28981.

[19] E. D. Glendening, J. Badenhoop, A. E. Reed, J. E. Carpenter, J. A. Bohmann, C. M. Morales, P. Karafiloglou, C. R. Landis, F. Weinhold, *Theor. Chem. Institute, Univ. Wisconsin, Madison* **2018**, NBO 7.0.

[20] J. Grèbol-Tomàs, E. Matito, P. Salvador, *Chem. – A Eur. J.* **2024**, *30*, e202401282.

[21] F. Weigend, R. Ahlrichs, *Phys. Chem. Chem. Phys.* **2005**, *7*, 3297–3305.

[22] S. Grimme, J. Antony, S. Ehrlich, H. Krieg, *J. Chem. Phys.* **2010**, *132*, 154104.

[23] S. Grimme, S. Ehrlich, L. Goerigk, *J. Comput. Chem.* **2011**, *32*, 1456–1465.

[24] R. Izsák, F. Neese, *J. Chem. Phys.* **2011**, *135*, 144105.

[25] A. D. Becke, *J. Chem. Phys.* **1993**, *98*, 5648–5652.

[26] C. Bannwarth, S. Ehlert, S. Grimme, *J. Chem. Theory Comput.* **2019**, *15*, 1652–1671.

[27] M. Garcia-Ratés, F. Neese, *J. Comput. Chem.* **2019**, *40*, 1816–1828.

[28] V. Ásgeirsson, B. O. Birgisson, R. Bjornsson, U. Becker, F. Neese, C. Riplinger, H. Jónsson, *J. Chem. Theory Comput.* **2021**, *17*, 4929–4945.

[29] M. Garcia-Ratés, F. Neese, *J. Comput. Chem.* **2020**, *41*, 922–939.

[30] G. Acke, S. Van Damme, R. W. A. Havenith, P. Bultinck, *J. Comput. Chem.* **2018**, *39*, 511–519.

[31] R. Ditchfield, *Mol. Phys.* **1974**, *27*, 789–807.

[32] R. Herges, D. Geuenich, *J. Phys. Chem. A* **2001**, *105*, 3214–3220.

[33] C. E. Colwell, T. W. Price, T. Stauch, R. Jasti, *Chem. Sci.* **2020**, *11*, 3923–3930.

[34] J. Zhu, K. An, P. von R. Schleyer, *Org. Lett.* **2013**, *15*, 2442–2445.

[35] D. Dunlop, L. Ludvíková, A. Banerjee, H. Ottosson, T. Slanina, *J. Am. Chem. Soc.* **2023**, *145*, 21569–21575.

[36] J. Michl, E. W. Thulstrup, *Tetrahedron* **1976**, *32*, 205–209.

[37] Y. Shibuya, K. Aonuma, T. Kimura, T. Kaneko, W. Fujiwara, Y. Yamaguchi, D. Kumaki, S. Tokito, H. Katagiri, *J. Phys. Chem. C* **2020**, *124*, 4738–4746.

[38] T. Yanai, D. P. Tew, N. C. Handy, *Chem. Phys. Lett.* **2004**, *393*, 51–57.

[39] E. R. Darzi, R. Jasti, *Chem. Soc. Rev.* **2015**, *44*, 6401–6410.
